# Supplementary material for: Interpreting population- and family-based genome-wide association studies in the presence of confounding
Source: PLoS Biol. 2024 Apr 11;22(4):e3002511. doi: 10.1371/journal.pbio.3002511 (PMC11008796; doi:10.1371/journal.pbio.3002511)
Supplement: S1 Text — (PDF) [file pbio.3002511.s001.pdf]

Supplementary information for:

Interpreting population- and family-based genome-wide association studies in the presence of confounding

Carl Veller, Graham Coop

S1 Genetic confounding in population and family-based GWAS designs

S1.1 The model

Under the general additive model we have studied, an individual's value for trait Y is

$$Y = Y^* + \sum_{l \in L} \alpha_l^{\mathrm{d}} g_l + \sum_{l \in L} \alpha_l^{\mathrm{i,m}} g_l^{\mathrm{m}} + \sum_{l \in L} \alpha_l^{\mathrm{i,f}} g_l^{\mathrm{f}} + \epsilon, \tag{S.1}$$

where g_l is the number of focal alleles at locus l carried by the individual, α_l^{d} is the direct genetic effect on the trait value of the focal allele at l (which we assume to be positive, without loss of generality), g_l^{m} and g_l^{f} are the numbers of copies of the focal allele at locus l carried by the individual's mother and father respectively, and $\alpha_l^{\mathrm{i,m}}$ and $\alpha_l^{\mathrm{i,f}}$ are the indirect genetic effects of the focal allele at l via the mother's and father's genotype respectively. ϵ is the environmental disturbance, with mean zero, and Y^* is the expected trait value of the offspring of parents who carry only trait-decreasing alleles.

It will be useful to expand Eq. (S.1) in terms of the individual's and the individual's parents' maternally and paternally inherited genotypes:

$$Y = Y^* + \sum_{l \in L} \alpha_l^{\mathrm{d}} \left(g_l^{\mathrm{mat}} + g_l^{\mathrm{pat}} \right) + \sum_{l \in L} \alpha_l^{\mathrm{i,m}} \left(g_l^{\mathrm{m,mat}} + g_l^{\mathrm{m,pat}} \right) + \sum_{l \in L} \alpha_l^{\mathrm{i,f}} \left(g_l^{\mathrm{f,mat}} + g_l^{\mathrm{f,pat}} \right) + \epsilon, \tag{S.2}$$

where g_l^{mat} is the number of focal alleles at locus l that the individual inherited maternally, $g_l^{\mathrm{m,mat}}$ is the number of focal alleles at l that the individual's mother inherited maternally, etc.

S1.2 Population GWAS

If we perform a standard population GWAS at a genotyped locus λ, the estimated effect of the focal allele at λ on the trait Y is

$$\hat{\alpha}_\lambda^{\mathrm{pop}} = \frac{\mathrm{Cov}(g_\lambda, Y)}{\mathrm{Var}(g_\lambda)}. \tag{S.3}$$

Here, $\mathrm{Var}(g_\lambda)$ is the genotypic variance at λ among sampled individuals, equal to $2p_\lambda(1-p_\lambda)(1+F_\lambda)$, where p_λ is the frequency of the focal allele at λ and F_λ is the coefficient of inbreeding at λ. For example, if λ is at Hardy-Weinberg equilibrium, then $\mathrm{Var}(g_\lambda) = 2p_\lambda(1-p_\lambda)$; if, instead, the population is divided into several populations, in each of which Hardy-Weinberg equilibrium obtains at λ but between which the frequency of the focal variant differs, then $\mathrm{Var}(g_\lambda) = 2p_\lambda(1-p_\lambda)(1+F_{ST,\lambda})$, where $F_{ST,\lambda}$ is the value of F_{ST} at locus λ.

Note that, here and throughout, we use hat notation ($\hat{\alpha}$) to denote estimates produced by regression, rather than to denote estimators (as is more usual); in all cases, the estimates are to be interpreted asymptotically or as expectations.

The covariance term in Eq. (S.3) expands out to

$$
\begin{aligned}
\mathrm{Cov}(g_\lambda, Y) &= \mathrm{Cov}\left(g_\lambda^{\mathrm{mat}} + g_\lambda^{\mathrm{pat}}\ ,\ Y^* + \sum_{l\in L} \alpha_l^{\mathrm{d}} \left(g_l^{\mathrm{mat}} + g_l^{\mathrm{pat}}\right)\right. \\
&\qquad\qquad \left. + \sum_{l\in L} \alpha_l^{\mathrm{i,m}} \left(g_l^{\mathrm{m,mat}} + g_l^{\mathrm{m,pat}}\right) + \sum_{l\in L} \alpha_l^{\mathrm{i,f}} \left(g_l^{\mathrm{f,mat}} + g_l^{\mathrm{f,pat}}\right) + \epsilon\right) \\
&= \mathrm{Cov}\left(g_\lambda^{\mathrm{mat}} + g_\lambda^{\mathrm{pat}}\ ,\ \sum_{l\in L} \alpha_l^{\mathrm{d}} \left(g_l^{\mathrm{mat}} + g_l^{\mathrm{pat}}\right)\right) \\
&\quad + \mathrm{Cov}\left(g_\lambda^{\mathrm{mat}}\ ,\ \sum_{l\in L} \alpha_l^{\mathrm{i,m}} \left(g_l^{\mathrm{m,mat}} + g_l^{\mathrm{m,pat}}\right) + \sum_{l\in L} \alpha_l^{\mathrm{i,f}} \left(g_l^{\mathrm{f,mat}} + g_l^{\mathrm{f,pat}}\right)\right) \\
&\quad + \mathrm{Cov}\left(g_\lambda^{\mathrm{pat}}\ ,\ \sum_{l\in L} \alpha_l^{\mathrm{i,m}} \left(g_l^{\mathrm{m,mat}} + g_l^{\mathrm{m,pat}}\right) + \sum_{l\in L} \alpha_l^{\mathrm{i,f}} \left(g_l^{\mathrm{f,mat}} + g_l^{\mathrm{f,pat}}\right)\right) + \mathrm{Cov}(g_\lambda, \epsilon) \\
&= \sum_{l\in L} \left(\left[\mathrm{Cov}\left(g_\lambda^{\mathrm{mat}}, g_l^{\mathrm{mat}}\right) + \mathrm{Cov}\left(g_\lambda^{\mathrm{mat}}, g_l^{\mathrm{pat}}\right) + \mathrm{Cov}\left(g_\lambda^{\mathrm{pat}}, g_l^{\mathrm{mat}}\right) + \mathrm{Cov}\left(g_\lambda^{\mathrm{pat}}, g_l^{\mathrm{pat}}\right)\right] \alpha_l^{\mathrm{d}} \right. \\
&\qquad + \left[\mathrm{Cov}\left(g_\lambda^{\mathrm{mat}}\ ,\ g_l^{\mathrm{m,mat}} + g_l^{\mathrm{m,pat}}\right)\right] \alpha_l^{\mathrm{i,m}} + \left[\mathrm{Cov}\left(g_\lambda^{\mathrm{pat}}\ ,\ g_l^{\mathrm{f,mat}} + g_l^{\mathrm{f,pat}}\right)\right] \alpha_l^{\mathrm{i,f}} \\
&\qquad \left. + \left[\mathrm{Cov}\left(g_\lambda^{\mathrm{mat}}, g_l^{\mathrm{f}}\right)\right] \alpha_l^{\mathrm{i,f}} + \left[\mathrm{Cov}\left(g_\lambda^{\mathrm{pat}}, g_l^{\mathrm{m}}\right)\right] \alpha_l^{\mathrm{i,m}} \right) + \mathrm{Cov}(g_\lambda, \epsilon) \\
&= \sum_{l\in L} \left(2\left(D_{\lambda l} + \tilde{D}_{\lambda l}\right) \alpha_l^{\mathrm{d}} \right. \\
&\qquad + \left[\mathrm{Cov}\left(g_\lambda^{\mathrm{mat}}\ ,\ g_l^{\mathrm{m,mat}} + g_l^{\mathrm{m,pat}}\right)\right] \alpha_l^{\mathrm{i,m}} + \left[\mathrm{Cov}\left(g_\lambda^{\mathrm{pat}}\ ,\ g_l^{\mathrm{f,mat}} + g_l^{\mathrm{f,pat}}\right)\right] \alpha_l^{\mathrm{i,f}} \\
&\qquad \left. + \left[\mathrm{Cov}\left(g_\lambda^{\mathrm{mat}}, g_l^{\mathrm{f}}\right)\right] \alpha_l^{\mathrm{i,f}} + \left[\mathrm{Cov}\left(g_\lambda^{\mathrm{pat}}, g_l^{\mathrm{m}}\right)\right] \alpha_l^{\mathrm{i,m}} \right) + \mathrm{Cov}(g_\lambda, \epsilon),
\end{aligned} \tag{S.4}
$$

where $D_{\lambda l}$ and $\tilde{D}_{\lambda l}$ are the degrees of cis- and trans-linkage disequilibrium between the focal alleles at loci λ and l in the GWAS sample. Since g_λ^{mat} equals $g_\lambda^{\mathrm{m,mat}}$ or $g_\lambda^{\mathrm{m,pat}}$ with equal probability, $\mathrm{Cov}\left(g_\lambda^{\mathrm{mat}}\ ,\ g_l^{\mathrm{m,mat}} + g_l^{\mathrm{m,pat}}\right) = D'_{\lambda l} + \tilde{D}'_{\lambda l}$, and similarly, $\mathrm{Cov}\left(g_\lambda^{\mathrm{pat}}\ ,\ g_l^{\mathrm{f,mat}} + g_l^{\mathrm{f,pat}}\right) = D'_{\lambda l} + \tilde{D}'_{\lambda l}$ (here, $D'_{\lambda l}$ and $\tilde{D}'_{\lambda l}$ are the LDs in the parents of the sample, assumed to be equal across mothers and fathers). Since maternal transmission is independent of paternal genotype, and vice versa, $\mathrm{Cov}\left(g_\lambda^{\mathrm{mat}}, g_l^{\mathrm{f}}\right) = \mathrm{Cov}\left(g_\lambda^{\mathrm{m}}, g_l^{\mathrm{f}}\right)/2$ and $\mathrm{Cov}\left(g_\lambda^{\mathrm{pat}}, g_l^{\mathrm{m}}\right) = \mathrm{Cov}\left(g_\lambda^{\mathrm{f}}, g_l^{\mathrm{m}}\right)/2$. So

$$
\begin{aligned}
\mathrm{Cov}(g_\lambda, Y) = \sum_{l\in L} &\left(2\left(D_{\lambda l} + \tilde{D}_{\lambda l}\right) \alpha_l^{\mathrm{d}} + \left(D'_{\lambda l} + \tilde{D}'_{\lambda l}\right)\left(\alpha_l^{\mathrm{i,m}} + \alpha_l^{\mathrm{i,f}}\right) \right. \\
&\qquad \left. + \frac{1}{2}\left[\mathrm{Cov}\left(g_\lambda^{\mathrm{m}}, g_l^{\mathrm{f}}\right)\right] \alpha_l^{\mathrm{i,f}} + \frac{1}{2}\left[\mathrm{Cov}\left(g_\lambda^{\mathrm{f}}, g_l^{\mathrm{m}}\right)\right] \alpha_l^{\mathrm{i,m}} \right) + \mathrm{Cov}(g_\lambda, \epsilon).
\end{aligned} \tag{S.5}
$$

If $\alpha_l^{\mathrm{i,m}} = \alpha_l^{\mathrm{i,f}} = \alpha_l^{\mathrm{i}}$, then

$$\begin{aligned}
\mathrm{Cov}(g_\lambda, Y) &= \sum_{l \in L} \left(2\left(D_{\lambda l} + \tilde{D}_{\lambda l}\right)\alpha_l^{\mathrm{d}} + \left(2\left(D'_{\lambda l} + \tilde{D}'_{\lambda l}\right) + \frac{1}{2}\left[\mathrm{Cov}\left(g_\lambda^{\mathrm{m}}, g_l^{\mathrm{f}}\right) + \mathrm{Cov}\left(g_\lambda^{\mathrm{f}}, g_l^{\mathrm{m}}\right)\right]\right)\alpha_l^{\mathrm{i}} \right) + \mathrm{Cov}(g_\lambda, \epsilon) \\
&= \sum_{l \in L} \left(2\left(D_{\lambda l} + \tilde{D}_{\lambda l}\right)\alpha_l^{\mathrm{d}} + \left(2\left(D'_{\lambda l} + \tilde{D}'_{\lambda l}\right) + \frac{1}{2}\left[8\tilde{D}_{\lambda l}\right]\right)\alpha_l^{\mathrm{i}} \right) + \mathrm{Cov}(g_\lambda, \epsilon) \\
&= 2\sum_{l \in L} \left(\left(D_{\lambda l} + \tilde{D}_{\lambda l}\right)\alpha_l^{\mathrm{d}} + \left(D'_{\lambda l} + \tilde{D}'_{\lambda l} + 2\tilde{D}_{\lambda l}\right)\alpha_l^{\mathrm{i}} \right) + \mathrm{Cov}(g_\lambda, \epsilon). \qquad \text{(S.6)}
\end{aligned}$$

In the second line of Eq. (S.6), we have used the fact that covariances across parents translate to covariances across maternal and paternal genomes in the offspring. Note, however, that $\mathrm{Cov}\left(g_\lambda^{\mathrm{m}}, g_l^{\mathrm{f}}\right)$ and $\mathrm{Cov}\left(g_\lambda^{\mathrm{f}}, g_l^{\mathrm{m}}\right)$ need not, in general be equal—e.g., they will not be so under sex-based cross-trait assortative mating—which is why we could not apply a similar simplification to Eq. (S.5).

Dividing Eq. (S.6) by $\mathrm{Var}(g_\lambda)$, and recognizing that, for $l \in L_{\mathrm{local}}$, $c_{\lambda l} \approx 0$, we recover Eq. (3) in the Main Text.

S1.3 Sibling GWAS

Consider two full siblings. Let $g_l^{\mathrm{mat},1}$ and $g_l^{\mathrm{mat},2}$ indicate whether sib 1 and sib 2 respectively inherited the focal (trait-increasing) allele from their mother at locus l. Let $g_l^{\mathrm{pat},1}$ and $g_l^{\mathrm{pat},2}$ be analogous indicators for paternal transmission. Write $\Delta g_l^{\mathrm{mat}} = g_l^{\mathrm{mat},1} - g_l^{\mathrm{mat},2}$ and $\Delta g_l^{\mathrm{pat}} = g_l^{\mathrm{pat},1} - g_l^{\mathrm{pat},2}$. Since maternal and paternal transmission are independent, $\Delta g_l^{\mathrm{mat}}$ and $\Delta g_{l'}^{\mathrm{pat}}$ are independent for all pairs of loci l and l' (including $l = l'$). The difference in the two siblings' genotypic values at locus l is $\Delta g_l = \Delta g_l^{\mathrm{mat}} + \Delta g_l^{\mathrm{pat}}$. From Eq. (S.1), the difference in their trait values is

$$\Delta Y = \sum_{l \in L} \Delta g_l \alpha_l^{\mathrm{d}} + \Delta\epsilon, \qquad \text{(S.7)}$$

where $\Delta\epsilon$ is the difference in the environmental disturbances experienced by the two siblings. Notice that the indirect effects cancel out of Eq. (S.7), since the parental genotypes are the same for the two siblings. So, in a sib-GWAS for trait Y, the estimated effect size at λ is

$$\begin{aligned}
\hat{\alpha}_\lambda^{\mathrm{sib}} &= \frac{\mathrm{Cov}\left(\Delta g_\lambda, \Delta Y\right)}{\mathrm{Var}\left(\Delta g_\lambda\right)} = \frac{\mathrm{Cov}\left(\Delta g_\lambda, \sum_{l \in L} \Delta g_l \alpha_l^{\mathrm{d}} + \Delta\epsilon\right)}{\mathrm{Var}\left(\Delta g_\lambda\right)} \\
&= \frac{\mathrm{Cov}\Big(\Delta g_\lambda^{\mathrm{mat}} + \Delta g_\lambda^{\mathrm{pat}} \ \ , \ \sum_{l \in L}\left(\Delta g_l^{\mathrm{mat}} + \Delta g_l^{\mathrm{pat}}\right)\alpha_l^{\mathrm{d}}\Big) + \mathrm{Cov}(\Delta g_\lambda, \Delta\epsilon)}{\mathrm{Var}\left(\Delta g_\lambda^{\mathrm{mat}} + \Delta g_\lambda^{\mathrm{pat}}\right)} \\
&= \frac{\sum_{l \in L}\left[\mathrm{Cov}\left(\Delta g_\lambda^{\mathrm{mat}}, \Delta g_l^{\mathrm{mat}}\right) + \mathrm{Cov}\left(\Delta g_\lambda^{\mathrm{pat}}, \Delta g_l^{\mathrm{pat}}\right)\right]\alpha_l^{\mathrm{d}}}{\mathrm{Var}\left(\Delta g_\lambda^{\mathrm{mat}}\right) + \mathrm{Var}\left(\Delta g_\lambda^{\mathrm{pat}}\right)} \\
&= \frac{\sum_{l \in L}\left(\mathbb{E}\left[\Delta g_\lambda^{\mathrm{mat}}\Delta g_l^{\mathrm{mat}}\right] + \mathbb{E}\left[\Delta g_\lambda^{\mathrm{pat}}\Delta g_l^{\mathrm{pat}}\right]\right)\alpha_l^{\mathrm{d}}}{\mathbb{E}\left[\left(\Delta g_\lambda^{\mathrm{mat}}\right)^2\right] + \mathbb{E}\left[\left(\Delta g_\lambda^{\mathrm{pat}}\right)^2\right]},
\end{aligned}$$

since $\mathrm{Cov}(\Delta g_\lambda, \Delta\epsilon) = 0$ (line 3), $\mathrm{Cov}\left(\Delta g_\lambda^{\mathrm{mat}}, \Delta g_l^{\mathrm{pat}}\right) = \mathrm{Cov}\left(\Delta g_l^{\mathrm{mat}}, \Delta g_\lambda^{\mathrm{pat}}\right) = 0$ (line 3) and $\mathbb{E}\left[\Delta g_k^{\mathrm{mat}}\right] = \mathbb{E}\left[\Delta g_k^{\mathrm{pat}}\right] = 0$ for all loci k (line 4). The denominator $\mathbb{E}\left[\left(\Delta g_\lambda^{\mathrm{mat}}\right)^2\right] + \mathbb{E}\left[\left(\Delta g_\lambda^{\mathrm{pat}}\right)^2\right] = H_\lambda$, the fraction of parents in the family GWAS sample who are heterozygous at locus λ. The only non-zero contributions to $\mathbb{E}\left[\Delta g_\lambda^{\mathrm{mat}} \Delta g_l^{\mathrm{mat}}\right]$ and $\mathbb{E}\left[\Delta g_\lambda^{\mathrm{pat}} \Delta g_l^{\mathrm{pat}}\right]$ come from parents who are heterozygous at both λ and l. Such parents are either 'coupling' double-heterozygotes carrying the focal alleles at λ and l in coupling phase (i.e., inherited from the same parent), or 'repulsion' double-heterozygotes carrying the focal alleles at λ and l in repulsion phase (inherited from different parents). Among parents, let the fractions of coupling and repulsion double-hets for loci λ and l be $H_{\lambda l}^{\mathrm{coup}}$ and $H_{\lambda l}^{\mathrm{rep}}$ respectively. If the recombination rate between the loci is $c_{\lambda l}^{♀}$ in females and $c_{\lambda l}^{♂}$ in males, then

$$\begin{aligned}\mathbb{E}\left[\Delta g_\lambda^{\mathrm{mat}} \Delta g_l^{\mathrm{mat}}\right] = \mathbb{E}&\left[\Delta g_\lambda^{\mathrm{mat}} \Delta g_l^{\mathrm{mat}} \,|\, \text{mother is coupling double-het}\right] H_{\lambda l}^{\mathrm{coup}} \\ &+ \mathbb{E}\left[\Delta g_\lambda^{\mathrm{mat}} \Delta g_l^{\mathrm{mat}} \,|\, \text{mother is repulsion double-het}\right] H_{\lambda l}^{\mathrm{rep}}.\end{aligned}$$

If the mother is a coupling double-heterozygote, then, for $\Delta g_\lambda^{\mathrm{mat}} \Delta g_l^{\mathrm{mat}}$ to be non-zero, either (i) both gametes must be non-recombinant [probability $(1 - c_{\lambda l}^{♀})^2$], in which case $\Delta g_\lambda^{\mathrm{mat}} \Delta g_l^{\mathrm{mat}} = +1$ with probability 1/2 and $= 0$ with probability 1/2, or (ii) both gametes must be recombinant [probability $(c_{\lambda l}^{♀})^2$], in which case $\Delta g_\lambda^{\mathrm{mat}} \Delta g_l^{\mathrm{mat}} = -1$ with probability 1/2 and $= 0$ with probability 1/2. Similarly, if the mother is a repulsion double-heterozygote, for $\Delta g_\lambda^{\mathrm{mat}} \Delta g_l^{\mathrm{mat}}$ to be non-zero, either (i) both gametes must be non-recombinant [probability $(1 - c_{\lambda l}^{♀})^2$], in which case $\Delta g_\lambda^{\mathrm{mat}} \Delta g_l^{\mathrm{mat}} = -1$ with probability 1/2 and $= 0$ with probability 1/2, or (ii) both gametes must be recombinant [probability $(c_{\lambda l}^{♀})^2$], in which case $\Delta g_\lambda^{\mathrm{mat}} \Delta g_l^{\mathrm{mat}} = +1$ with probability 1/2 and $= 0$ with probability 1/2. Therefore,

$$\begin{aligned}\mathbb{E}\left[\Delta g_\lambda^{\mathrm{mat}} \Delta g_l^{\mathrm{mat}}\right] &= \left((1 - c_{\lambda l}^{♀})^2 \times (+1/2) + (c_{\lambda l}^{♀})^2 \times (-1/2)\right) H_{\lambda l}^{\mathrm{coup}} \\ &\quad + \left((1 - c_{\lambda l}^{♀})^2 \times (-1/2) + (c_{\lambda l}^{♀})^2 \times (+1/2)\right) H_{\lambda l}^{\mathrm{rep}} \\ &= \frac{1}{2}\left(1 - 2c_{\lambda l}^{♀}\right) H_{\lambda l}^{\mathrm{coup}} - \frac{1}{2}\left(1 - 2c_{\lambda l}^{♀}\right) H_{\lambda l}^{\mathrm{rep}} \\ &= \left(\frac{1}{2} - c_{\lambda l}^{♀}\right)\left(H_{\lambda l}^{\mathrm{coup}} - H_{\lambda l}^{\mathrm{rep}}\right) \\ &= \left(1 - 2c_{\lambda l}^{♀}\right)\left(D'_{\lambda l} - \tilde{D}'_{\lambda l}\right),\end{aligned}$$

since $H_{\lambda l}^{\mathrm{coup}} - H_{\lambda l}^{\mathrm{rep}} = 2(D'_{\lambda l} - \tilde{D}'_{\lambda l})$, where $D'_{\lambda l}$ and $\tilde{D}'_{\lambda l}$ are the cis- and trans-LD between the focal/trait-increasing alleles at λ and l among parents. Similarly,

$$\mathbb{E}\left[\Delta g_\lambda^{\mathrm{pat}} \Delta g_l^{\mathrm{pat}}\right] = \left(1 - 2c_{\lambda l}^{♂}\right)\left(D'_{\lambda l} - \tilde{D}'_{\lambda l}\right),$$

So

$$\hat{\alpha}_\lambda^{\mathrm{d,sib}} = \frac{2\sum_{l \in L}\left(1 - 2c_{\lambda l}\right)\left(D'_{\lambda l} - \tilde{D}'_{\lambda l}\right)\alpha_l^{\mathrm{d}}}{H_\lambda}, \tag{S.8}$$

where $c_{\lambda l}$ is the sex-averaged recombination fraction between λ and l. Recognizing that, for $l \in L_{\mathrm{local}}$, $c_{\lambda l} \approx 0$ and $|\tilde{D}_{\lambda l}| \ll |D_{\lambda l}|$ in expectation, we recover Eq. (7) in the Main Text:

$$\hat{\alpha}_\lambda^{\mathrm{d,sib}} \approx \frac{2}{H_\lambda}\left(\sum_{l \in L_{\mathrm{local}}} D'_{\lambda l}\alpha_l^{\mathrm{d}} + \sum_{l \in L \setminus L_{\mathrm{local}}}\left(1 - 2c_{\lambda l}\right)\left(D'_{\lambda l} - \tilde{D}'_{\lambda l}\right)\alpha_l^{\mathrm{d}}\right). \tag{S.9}$$

S1.4 Transmitted–untransmitted GWAS

In Eq. (S.2), g_l^{mat} represents the allele that was transmitted maternally from among the set of maternal alleles $\left\{g_l^{\text{m,mat}}, g_l^{\text{m,pat}}\right\}$. Thus, if the maternally transmitted allele was the grandmaternal allele (with probability 1/2, and in which case $g_l^{\text{mat}} = g_l^{\text{m,mat}}$), then the untransmitted allele at locus l is the grandpaternal allele, with genotypic value $g_l^{\text{m,pat}}$. To make this distinction clear, we write g_l^{matT} for the genotypic value of the maternally transmitted allele at locus l, and g_l^{matU} for the maternally untransmitted allele at locus l. Similarly, g_l^{patT} and g_l^{patU} represent the paternally transmitted and untransmitted alleles at l. The transmitted and untransmitted genotypes are $g_l^{\text{T}} = g_l^{\text{matT}} + g_l^{\text{patT}}$ and $g_l^{\text{U}} = g_l^{\text{matU}} + g_l^{\text{patU}}$ respectively.

Estimating direct effects

We are interested in the coefficients produced by the joint regression of the trait value on the transmitted and untransmitted genotype at locus λ, $\hat{\alpha}_\lambda^{(\text{T})}$ and $\hat{\alpha}_\lambda^{(\text{U})}$.

It is straightforward to show that the difference between these coefficients—the estimate of the direct effect at λ in the transmitted-untransmitted study design—is the same in expectation as the sibling-based estimate of the direct effect calculated above (e.g., [1]). Noting that $g_\lambda^{\text{T}} = g_\lambda$, the offspring's genotype at λ, and $g_\lambda^{\text{T}} + g_\lambda^{\text{U}} = g_\lambda^m + g_\lambda^f$, the sum of the maternal and paternal genotypes, the regression that we wish to estimate,

$$Y = k + \alpha_\lambda^{(\text{T})} g_\lambda^{\text{T}} + \alpha_\lambda^{(\text{U})} g_\lambda^{\text{U}} + \varepsilon, \tag{S.10}$$

can be rewritten

$$\begin{aligned}
Y &= k + \alpha_\lambda^{(\text{T})} g_\lambda^{\text{T}} + \alpha_\lambda^{(\text{U})} g_\lambda^{\text{U}} + \varepsilon \\
&= k + \alpha_\lambda^{(\text{T})} g_\lambda^{\text{T}} + \alpha_\lambda^{(\text{U})} \left(g_\lambda^m + g_\lambda^f - g_\lambda^{\text{T}}\right) + \varepsilon \\
&= k + \left(\alpha_\lambda^{(\text{T})} - \alpha_\lambda^{(\text{U})}\right) g_\lambda^{\text{T}} + \alpha_\lambda^{(\text{U})} \left(g_\lambda^m + g_\lambda^f\right) + \varepsilon \\
&= k + \left(\alpha_\lambda^{(\text{T})} - \alpha_\lambda^{(\text{U})}\right) g_\lambda + 2\alpha_\lambda^{(\text{U})} \frac{g_\lambda^m + g_\lambda^f}{2} + \varepsilon, \qquad (\text{S.11})
\end{aligned}$$

i.e., the regression of offspring trait value on offspring genotype at λ, controlling for the midparent genotype. Therefore, the estimate of the direct effect $\hat{\alpha}_\lambda^{(\text{T})} - \hat{\alpha}_\lambda^{(\text{U})}$ obtained by OLS estimation of Eq. (S.10) is the same as the coefficient on g_λ obtained by OLS estimation of Eq. (S.11).

By the Frisch–Waugh–Lovell theorem, the OLS estimate of the coefficient on g_λ in Eq. (S.11) is the same as would be obtained if we regress g_λ on the midparent genotype $\left(g_\lambda^m + g_\lambda^f\right)/2$, obtain the residuals, and regress the offspring trait values Y on these residuals. But the first regression is just

$$g_\lambda = \frac{g_\lambda^m + g_\lambda^f}{2} + \varsigma_\lambda,$$

where ς_λ is the deviation of the offspring's genotype from the midparent value due to the randomness of segregation at λ. Therefore, the residuals in the first-stage regression are the ς_λ in expectation, and so the coefficient on g_λ obtained by OLS estimation of (S.11)—and therefore the estimate of the direct effect in the transmitted-untransmitted association study at λ—is the same as that obtained by OLS estimation of the regression of offspring trait value on the genotypic deviation ς_λ, i.e.,

$$\hat{\alpha}_\lambda^{\text{T-U}} = \frac{\text{Cov}(\varsigma_\lambda, Y)}{\text{Var}(\varsigma_\lambda)}. \tag{S.12}$$

To see that this is the same, in expectation, as the estimate of the direct effect produced by the sibling association study, note that the difference between the genotypes of two siblings 1 and 2 at locus λ is $\Delta g_\lambda = \varsigma_\lambda^1 - \varsigma_\lambda^2$, and, also, that the segregation deviations ς_λ^1 and ς_λ^2 are uncorrelated. The estimate of the direct effect in the sibling-based regression can therefore be written

$$\begin{aligned}
\hat{\alpha}_\lambda^{\text{sib}} = \frac{\text{Cov}(\Delta g_\lambda, \Delta Y)}{\text{Var}(\Delta g_\lambda)} &= \frac{\text{Cov}(\varsigma_\lambda^1 - \varsigma_\lambda^2, Y^1 - Y^2)}{\text{Var}(\varsigma_\lambda^1 - \varsigma_\lambda^2)} \\
&= \frac{\text{Cov}(\varsigma_\lambda^1 - \varsigma_\lambda^2, Y^1 - Y^2)}{2\text{Var}(\varsigma_\lambda)} \qquad \text{(linear independence of } \varsigma_\lambda^1, \varsigma_\lambda^2\text{)} \\
&= \frac{\text{Cov}(\varsigma_\lambda^1, Y^1) + \text{Cov}(\varsigma_\lambda^2, Y^2) - \text{Cov}(\varsigma_\lambda^1, Y^2) - \text{Cov}(\varsigma_\lambda^2, Y^1)}{2\text{Var}(\varsigma_\lambda)} \\
&= \frac{\text{Cov}(\varsigma_\lambda^1, Y^1) + \text{Cov}(\varsigma_\lambda^2, Y^2)}{2\text{Var}(\varsigma_\lambda)} \qquad \text{(no sibling indirect effects)} \\
&= \frac{2\text{Cov}(\varsigma_\lambda, Y)}{2\text{Var}(\varsigma_\lambda)} = \frac{\text{Cov}(\varsigma_\lambda, Y)}{\text{Var}(\varsigma_\lambda)},
\end{aligned} \tag{S.13}$$

which is the same as Eq. (S.12).

Therefore, under the assumption of no sibling indirect effects (line 4 in Eq. S.13 above), the sibling and the transmitted-untransmitted estimates of the direct effect of the focal variant at λ are equal in expectation, and so

$$\begin{aligned}
\hat{\alpha}_\lambda^{\text{d,T-U}} = \hat{\alpha}_\lambda^{\text{sib}} &= \frac{2\sum_{l\in L}(1-2c_{\lambda l})\left(D'_{\lambda l} - \tilde{D}'_{\lambda l}\right)\alpha_l^{\text{d}}}{H_\lambda} \\
&\approx \frac{2}{H_\lambda}\left(\sum_{l\in L_{\text{local}}} D'_{\lambda l}\alpha_l^{\text{d}} + \sum_{l\in L\setminus L_{\text{local}}}(1-2c_{\lambda l})\left(D'_{\lambda l} - \tilde{D}'_{\lambda l}\right)\alpha_l^{\text{d}}\right).
\end{aligned} \tag{S.14}$$

Estimating indirect effects

The estimated coefficient on the untransmitted genotype g_λ^{U} in the regression (S.10) has sometimes been interpreted as an estimate of the indirect 'family' effect of the focal variant at λ: $\hat{\alpha}_\lambda^{\text{i}} = \hat{\alpha}_\lambda^{\text{U}}$. Here, we calculate the value of this estimate under the phenotypic model (S.1).

Let Y_i, $g_{\lambda,i}^{\text{T}}$, and $g_{\lambda,i}^{\text{U}}$ be the trait value, transmitted genotype, and untransmitted genotype of offspring i, and let $\boldsymbol{Y}$, $\boldsymbol{g_\lambda^{\text{T}}}$, and $\boldsymbol{g_\lambda^{\text{U}}}$ be the corresponding $n \times 1$ vectors of these values across all n offspring. Define the $n \times 3$ matrix $\boldsymbol{X} = [\boldsymbol{1}, \boldsymbol{g_\lambda^{\text{T}}}, \boldsymbol{g_\lambda^{\text{U}}}]$ where $\boldsymbol{1}$ is an $n \times 1$ vectors of 1s. Then the coefficients produced by OLS estimation of Eq. (S.10) are

$$\hat{\boldsymbol{\alpha}} = \begin{pmatrix} \hat{k} \\ \hat{\alpha}_\lambda^{\text{T}} \\ \hat{\alpha}_\lambda^{\text{U}} \end{pmatrix} = (\boldsymbol{X}'\boldsymbol{X})^{-1}\boldsymbol{X}'\boldsymbol{Y}, \tag{S.15}$$

where $\boldsymbol{X}'$ is the transpose of $\boldsymbol{X}$. To calculate Eq. (S.15), we first calculate that

$$\begin{aligned}
\boldsymbol{X}'\boldsymbol{X} &= n\begin{pmatrix} 1 & \frac{1}{n}\sum_{i=1}^{n} g_{\lambda,i}^{\mathrm{T}} & \frac{1}{n}\sum_{i=1}^{n} g_{\lambda,i}^{\mathrm{U}} \\ \frac{1}{n}\sum_{i=1}^{n} g_{\lambda,i}^{\mathrm{T}} & \frac{1}{n}\sum_{i=1}^{n}(g_{\lambda,i}^{\mathrm{T}})^2 & \frac{1}{n}\sum_{i=1}^{n} g_{\lambda,i}^{\mathrm{T}} g_{\lambda,i}^{\mathrm{U}} \\ \frac{1}{n}\sum_{i=1}^{n} g_{\lambda,i}^{\mathrm{U}} & \frac{1}{n}\sum_{i=1}^{n} g_{\lambda,i}^{\mathrm{T}} g_{\lambda,i}^{\mathrm{U}} & \frac{1}{n}\sum_{i=1}^{n}(g_{\lambda,i}^{\mathrm{U}})^2 \end{pmatrix} \\
&= n\begin{pmatrix} 1 & \mathbb{E}[g_\lambda^{\mathrm{T}}] & \mathbb{E}[g_\lambda^{\mathrm{U}}] \\ \mathbb{E}[g_\lambda^{\mathrm{T}}] & \mathrm{Var}(g_\lambda^{\mathrm{T}}) + (\mathbb{E}[g_\lambda^{\mathrm{T}}])^2 & \mathrm{Cov}(g_\lambda^{\mathrm{T}}, g_\lambda^{\mathrm{U}}) + \mathbb{E}[g_\lambda^{\mathrm{T}}]\mathbb{E}[g_\lambda^{\mathrm{U}}] \\ \mathbb{E}[g_\lambda^{\mathrm{U}}] & \mathrm{Cov}(g_\lambda^{\mathrm{T}}, g_\lambda^{\mathrm{U}}) + \mathbb{E}[g_\lambda^{\mathrm{T}}]\mathbb{E}[g_\lambda^{\mathrm{U}}] & \mathrm{Var}(g_\lambda^{\mathrm{U}}) + (\mathbb{E}[g_\lambda^{\mathrm{U}}])^2 \end{pmatrix}, \qquad \text{(S.16)}
\end{aligned}$$

where the expectations, variances, and covariances in Eq. (S.16) are treated as sample averages across the entire sample of offspring. If p is the frequency of the focal allele at λ among offspring in the sample (and therefore also among their parents), and F and F' are the inbreeding coefficients amongst offspring and parents respectively at the locus, then we have $\mathbb{E}[g_\lambda^{\mathrm{T}}] = \mathbb{E}[g_\lambda^{\mathrm{U}}] = 2p$ and $\mathrm{Var}(g_\lambda^{\mathrm{T}}) = \mathrm{Var}(g_\lambda^{\mathrm{U}}) = 2p(1-p)(1+F)$. For $\mathrm{Cov}(g_\lambda^{\mathrm{T}}, g_\lambda^{\mathrm{U}})$, write $g_\lambda^{\mathrm{T}} = g_\lambda^{\mathrm{matT}} + g_\lambda^{\mathrm{patT}}$ and $g_\lambda^{\mathrm{U}} = g_\lambda^{\mathrm{matU}} + g_\lambda^{\mathrm{patU}}$ as before. Since which allele was transmitted from each parent to the offspring is random, $\mathrm{Cov}(g_\lambda^{\mathrm{matT}}, g_\lambda^{\mathrm{patU}}) = \mathrm{Cov}(g_\lambda^{\mathrm{matU}}, g_\lambda^{\mathrm{patT}}) = \mathrm{Cov}(g_\lambda^{\mathrm{matT}}, g_\lambda^{\mathrm{patT}})$. Therefore,

$$\begin{aligned}
\mathrm{Cov}(g_\lambda^{\mathrm{T}}, g_\lambda^{\mathrm{U}}) &= \mathrm{Cov}(g_\lambda^{\mathrm{matT}} + g_\lambda^{\mathrm{patT}}, g_\lambda^{\mathrm{matU}} + g_\lambda^{\mathrm{patU}}) \\
&= \mathrm{Cov}(g_\lambda^{\mathrm{matT}}, g_\lambda^{\mathrm{matU}}) + \mathrm{Cov}(g_\lambda^{\mathrm{patT}}, g_\lambda^{\mathrm{patU}}) + \mathrm{Cov}(g_\lambda^{\mathrm{matT}}, g_\lambda^{\mathrm{patU}}) + \mathrm{Cov}(g_\lambda^{\mathrm{matU}}, g_\lambda^{\mathrm{patT}}) \\
&= \mathrm{Cov}(g_\lambda^{\mathrm{matT}}, g_\lambda^{\mathrm{matU}}) + \mathrm{Cov}(g_\lambda^{\mathrm{patT}}, g_\lambda^{\mathrm{patU}}) + \mathrm{Cov}(g_\lambda^{\mathrm{matT}}, g_\lambda^{\mathrm{patT}}) + \mathrm{Cov}(g_\lambda^{\mathrm{matT}}, g_\lambda^{\mathrm{patT}}) \\
&= p(1-p)F' + p(1-p)F' + p(1-p)F + p(1-p)F \\
&= 4p(1-p)F,
\end{aligned}$$

where the penultimate line follows from the fact that $\mathrm{Cov}(g_\lambda^{\mathrm{matT}}, g_\lambda^{\mathrm{matU}})$ and $\mathrm{Cov}(g_\lambda^{\mathrm{patT}}, g_\lambda^{\mathrm{patU}})$ are allelic covariances in parents while $\mathrm{Cov}(g_\lambda^{\mathrm{matT}}, g_\lambda^{\mathrm{patT}})$ is the allelic covariance in offspring. In the last line, we have made the assumption that the inbreeding coefficients are the same in parents and offspring.

Substituting all of these values into Eq. (S.16),

$$\begin{aligned}
\boldsymbol{X}'\boldsymbol{X} &= n\begin{pmatrix} 1 & 2p & 2p \\ 2p & 2p(1-p)(1+F) + 4p^2 & 4p(1-p)F + 4p^2 \\ 2p & 4p(1-p)F + 4p^2 & 2p(1-p)(1+F) + 4p^2 \end{pmatrix} \\
&= n\begin{pmatrix} 1 & 2p & 2p \\ 2p & 2p[1+p(1-p)F] & 4p[p+(1-p)F] \\ 2p & 4p[p+(1-p)F] & 2p[1+p(1-p)F] \end{pmatrix}. \qquad \text{(S.17)}
\end{aligned}$$

From standard linear algebra, we calculate that

$$(\boldsymbol{X}'\boldsymbol{X})^{-1} = \frac{1}{n} \cdot \frac{1}{2p(1-p)(1+3F)(1-F)} \begin{pmatrix} \cdots & -2p(1-F) & -2p(1-F) \\ -2p(1-F) & 1+F & -2F \\ -2p(1-F) & -2F & 1+F \end{pmatrix}, \qquad \text{(S.18)}$$

where the term denoted by ellipses is $2p[1+3p+3(1-p)F](1-F)$ and will not matter in what follows.

Turning to the other term in Eq. (S.15),

$$\boldsymbol{X}'\boldsymbol{Y} = n\begin{pmatrix} \frac{1}{n}\sum_{i=1}^{n} Y_i \\ \frac{1}{n}\sum_{i=1}^{n} g_{\lambda,i}^{\mathrm{T}} Y_i \\ \frac{1}{n}\sum_{i=1}^{n} g_{\lambda,i}^{\mathrm{U}} Y_i \end{pmatrix} = n\begin{pmatrix} \mathbb{E}[Y] \\ \mathrm{Cov}(g_\lambda^{\mathrm{T}}, Y) + \mathbb{E}[g_\lambda^{\mathrm{T}}]\mathbb{E}[Y] \\ \mathrm{Cov}(g_\lambda^{\mathrm{U}}, Y) + \mathbb{E}[g_\lambda^{\mathrm{U}}]\mathbb{E}[Y] \end{pmatrix} = n\begin{pmatrix} \bar{Y} \\ \mathrm{Cov}(g_\lambda^{\mathrm{T}}, Y) + 2p\bar{Y} \\ \mathrm{Cov}(g_\lambda^{\mathrm{U}}, Y) + 2p\bar{Y} \end{pmatrix}. \qquad \text{(S.19)}$$

Therefore,

$$\hat{\boldsymbol{\alpha}} = \begin{pmatrix} \hat{k} \\ \hat{\alpha}_\lambda^{\mathrm{T}} \\ \hat{\alpha}_\lambda^{\mathrm{U}} \end{pmatrix} = (\boldsymbol{X}'\boldsymbol{X})^{-1}\boldsymbol{X}'\boldsymbol{Y}$$
$$= \frac{1}{2p(1-p)(1+3F)(1-F)} \begin{pmatrix} \cdots & -2p(1-F) & -2p(1-F) \\ -2p(1-F) & 1+F & -2F \\ -2p(1-F) & -2F & 1+F \end{pmatrix} \begin{pmatrix} \bar{Y} \\ \mathrm{Cov}(g_\lambda^{\mathrm{T}}, Y) + 2p\bar{Y} \\ \mathrm{Cov}(g_\lambda^{\mathrm{U}}, Y) + 2p\bar{Y} \end{pmatrix}. \tag{S.20}$$

So, to calculate the OLS estimates of the coefficients $\hat{\boldsymbol{\alpha}}$, it remains to calculate $\mathrm{Cov}\left(g_\lambda^{\mathrm{T}}, Y\right)$ and $\mathrm{Cov}\left(g_\lambda^{\mathrm{U}}, Y\right)$.

We have

$$\begin{aligned}
\mathrm{Cov}\left(g_\lambda^{\mathrm{matT}}, Y\right) &= \mathrm{Cov}\Bigg(g_\lambda^{\mathrm{matT}},\ \ Y^* + \sum_{l\in L}\left(g_l^{\mathrm{matT}} + g_l^{\mathrm{patT}}\right)\alpha_l^{\mathrm{d}} \\
&\qquad + \sum_{l\in L}\left(g_l^{\mathrm{m,mat}} + g_l^{\mathrm{m,pat}}\right)\alpha_l^{\mathrm{i,m}} + \sum_{l\in L}\left(g_l^{\mathrm{f,mat}} + g_l^{\mathrm{f,pat}}\right)\alpha_l^{\mathrm{i,f}} + \epsilon\Bigg) \\
&= \sum_{l\in L}\left[\mathrm{Cov}\left(g_\lambda^{\mathrm{matT}}, g_l^{\mathrm{matT}}\right) + \mathrm{Cov}\left(g_\lambda^{\mathrm{matT}}, g_l^{\mathrm{patT}}\right)\right]\alpha_l^{\mathrm{d}} \\
&\quad + \sum_{l\in L}\left[\mathrm{Cov}\left(g_\lambda^{\mathrm{matT}}, g_l^{\mathrm{m,mat}}\right) + \mathrm{Cov}\left(g_\lambda^{\mathrm{matT}}, g_l^{\mathrm{m,pat}}\right)\right]\alpha_l^{\mathrm{i,m}} \\
&\quad + \sum_{l\in L}\left[\mathrm{Cov}\left(g_\lambda^{\mathrm{matT}}, g_l^{\mathrm{f,mat}} + g_l^{\mathrm{f,pat}}\right)\right]\alpha_l^{\mathrm{i,f}} + \mathrm{Cov}\left(g_\lambda^{\mathrm{matT}}, \epsilon\right) \\
&= \sum_{l\in L}\left[D'_{\lambda l}\left(1 - c_{\lambda l}^{♀}\right) + \tilde{D}'_{\lambda l}c_{\lambda l}^{♀} + \mathrm{Cov}\left(g_\lambda^{\mathrm{matT}}, g_l^{\mathrm{patT}}\right)\right]\alpha_l^{\mathrm{d}} \\
&\quad + \sum_{l\in L}\left(D'_{\lambda l} + \tilde{D}'_{\lambda l}\right)\alpha_l^{\mathrm{i,m}} + \sum_{l\in L}\mathrm{Cov}\left(g_\lambda^{\mathrm{matT}}, g_l^{\mathrm{f}}\right)\alpha_l^{\mathrm{i,f}} + \mathrm{Cov}\left(g_\lambda^{\mathrm{matT}}, \epsilon\right),
\end{aligned}$$

and, similarly,

$$\begin{aligned}
\mathrm{Cov}\left(g_\lambda^{\mathrm{patT}}, Y\right) &= \sum_{l\in L}\left[D'_{\lambda l}\left(1 - c_{\lambda l}^{♂}\right) + \tilde{D}'_{\lambda l}c_{\lambda l}^{♂} + \mathrm{Cov}\left(g_\lambda^{\mathrm{patT}}, g_l^{\mathrm{matT}}\right)\right]\alpha_l^{\mathrm{d}} \\
&\quad + \sum_{l\in L}\left(D'_{\lambda l} + \tilde{D}'_{\lambda l}\right)\alpha_l^{\mathrm{i,f}} + \sum_{l\in L}\mathrm{Cov}\left(g_\lambda^{\mathrm{patT}}, g_l^{\mathrm{m}}\right)\alpha_l^{\mathrm{i,m}} + \mathrm{Cov}\left(g_\lambda^{\mathrm{patT}}, \epsilon\right).
\end{aligned}$$

Note that

$$\mathrm{Cov}\left(g_\lambda^{\mathrm{matT}}, g_l^{\mathrm{patT}}\right) + \mathrm{Cov}\left(g_\lambda^{\mathrm{patT}}, g_l^{\mathrm{matT}}\right) = 2\tilde{D}_{\lambda l} \tag{S.21}$$

and, because $\mathrm{Cov}\left(g_\lambda^{\mathrm{matT}}, g_l^{\mathrm{patT}}\right) = \mathrm{Cov}\left(g_\lambda^{\mathrm{matU}}, g_l^{\mathrm{patT}}\right)$ and $\mathrm{Cov}\left(g_\lambda^{\mathrm{patT}}, g_l^{\mathrm{matT}}\right) = \mathrm{Cov}\left(g_\lambda^{\mathrm{patU}}, g_l^{\mathrm{matT}}\right)$,

$$\begin{aligned}
\mathrm{Cov}\left(g_\lambda^{\mathrm{matT}}, g_l^{\mathrm{f}}\right) + \mathrm{Cov}\left(g_\lambda^{\mathrm{patT}}, g_l^{\mathrm{m}}\right) &= \mathrm{Cov}\left(g_\lambda^{\mathrm{matT}}, g_l^{\mathrm{patT}} + g_l^{\mathrm{patU}}\right) + \mathrm{Cov}\left(g_\lambda^{\mathrm{patT}}, g_l^{\mathrm{matT}} + g_l^{\mathrm{matU}}\right) \\
&= 2\mathrm{Cov}\left(g_\lambda^{\mathrm{matT}}, g_l^{\mathrm{patT}}\right) + \mathrm{Cov}\left(g_\lambda^{\mathrm{patT}}, g_l^{\mathrm{matT}}\right) = 4\tilde{D}_{\lambda l}.
\end{aligned} \tag{S.22}$$

The identities in Eqs. (S.21) and (S.22) allow for asymmetry across locus pairs in the maternal/paternal contributions to trans-LD—i.e., we need not assume that $\mathrm{Cov}\left(g_\lambda^{\mathrm{matT}}, g_l^{\mathrm{patT}}\right) = \mathrm{Cov}\left(g_\lambda^{\mathrm{patT}}, g_l^{\mathrm{matT}}\right)$. (Such an asymmetry could arise, for example, under sex-asymmetric models of mate choice, where one sex displays a mating preference for some phenotype in the other sex.)

Therefore,

$$\begin{aligned}\mathrm{Cov}\left(g_\lambda^{\mathrm{T}}, Y\right) &= \mathrm{Cov}\left(g_\lambda^{\mathrm{matT}} + g_\lambda^{\mathrm{patT}}, Y\right) = \mathrm{Cov}\left(g_\lambda^{\mathrm{matT}}, Y\right) + \mathrm{Cov}\left(g_\lambda^{\mathrm{patT}}, Y\right) \\ &= 2\sum_{l\in L}\left[D'_{\lambda l}\left(1-c_{\lambda l}\right) + \tilde{D}'_{\lambda l}c_{\lambda l} + \tilde{D}_{\lambda l}\right]\alpha_l^{\mathrm{d}} + \sum_{l\in L}\left(D'_{\lambda l} + \tilde{D}'_{\lambda l}\right)\left(\alpha_l^{\mathrm{i,f}} + \alpha_l^{\mathrm{i,m}}\right) \\ &\quad + \sum_{l\in L}\left[\mathrm{Cov}\left(g_\lambda^{\mathrm{matT}}, g_l^{\mathrm{f}}\right)\right]\alpha_l^{\mathrm{i,f}} + \sum_{l\in L}\left[\mathrm{Cov}\left(g_\lambda^{\mathrm{patT}}, g_l^{\mathrm{m}}\right)\right]\alpha_l^{\mathrm{i,m}} + \mathrm{Cov}\left(g_\lambda, \epsilon\right),\end{aligned} \tag{S.23}$$

where $c_{\lambda l}$ is the sex-averaged recombination fraction between λ and l. If we further assume that the indirect effects of the maternal and paternal genotype are equal ($\alpha_l^{\mathrm{i,m}} = \alpha_l^{\mathrm{i,f}} = \alpha_l^{\mathrm{i}}$), this expression simplifies to

$$\mathrm{Cov}\left(g_\lambda^{\mathrm{T}}, Y\right) = 2\sum_{l\in L}\left[D'_{\lambda l}\left(1-c_{\lambda l}\right) + \tilde{D}'_{\lambda l}c_{\lambda l} + \tilde{D}_{\lambda l}\right]\alpha_l^{\mathrm{d}} + 2\sum_{l\in L}\left(D'_{\lambda l} + \tilde{D}'_{\lambda l} + 2\tilde{D}_{\lambda l}\right)\alpha_l^{\mathrm{i}} + \mathrm{Cov}\left(g_\lambda, \epsilon\right). \tag{S.24}$$

We can perform similar calculations for the untransmitted genotype, again using the facts that $\mathrm{Cov}\left(g_\lambda^{\mathrm{matT}}, g_l^{\mathrm{patT}}\right) = \mathrm{Cov}\left(g_\lambda^{\mathrm{matU}}, g_l^{\mathrm{patT}}\right)$, $\mathrm{Cov}\left(g_\lambda^{\mathrm{patT}}, g_l^{\mathrm{matT}}\right) = \mathrm{Cov}\left(g_\lambda^{\mathrm{patU}}, g_l^{\mathrm{matT}}\right)$, $\mathrm{Cov}\left(g_\lambda^{\mathrm{matT}}, g_l^{\mathrm{f}}\right) = \mathrm{Cov}\left(g_\lambda^{\mathrm{matU}}, g_l^{\mathrm{f}}\right)$, and $\mathrm{Cov}\left(g_\lambda^{\mathrm{patT}}, g_l^{\mathrm{m}}\right) = \mathrm{Cov}\left(g_\lambda^{\mathrm{patU}}, g_l^{\mathrm{m}}\right)$. Assuming equal indirect effects from maternal and paternal genotype, we obtain

$$\mathrm{Cov}\left(g_\lambda^{\mathrm{U}}, Y\right) = 2\sum_{l\in L}\left[D'_{\lambda l}c_{\lambda l} + \tilde{D}'_{\lambda l}\left(1-c_{\lambda l}\right) + \tilde{D}_{\lambda l}\right]\alpha_l^{\mathrm{d}} + 2\sum_{l\in L}\left(D'_{\lambda l} + \tilde{D}'_{\lambda l} + 2\tilde{D}_{\lambda l}\right)\alpha_l^{\mathrm{i}} + \mathrm{Cov}\left(g_\lambda^{\mathrm{U}}, \epsilon\right). \tag{S.25}$$

Before we use these expressions to calculate the parent-offspring estimate of the indirect effect, $\hat{\alpha}_\lambda^{\mathrm{i,T\text{-}U}}$, it is worth quickly checking that we can use them to recover the same value for the parent-offspring estimate of the direct effect, $\hat{\alpha}_\lambda^{\mathrm{d,T\text{-}U}}$, that we calculated in Eq. (S.14). From Eqs. (S.20), (S.24), and

(S.25),

$$
\begin{aligned}
\hat{\alpha}_\lambda^{\mathrm{T}} - \hat{\alpha}_\lambda^{\mathrm{U}} &= \frac{\begin{pmatrix} -2p(1-F) & 1+F & -2F \end{pmatrix} - \begin{pmatrix} -2p(1-F) & -2F & 1+F \end{pmatrix}}{2p(1-p)(1+3F)(1-F)} \begin{pmatrix} \bar{Y} \\ \mathrm{Cov}(g_\lambda^{\mathrm{T}}, Y) + 2p\bar{Y} \\ \mathrm{Cov}(g_\lambda^{\mathrm{U}}, Y) + 2p\bar{Y} \end{pmatrix} \\
&= \frac{\begin{pmatrix} 0 & 1+3F & -(1+3F) \end{pmatrix}}{2p(1-p)(1+3F)(1-F)} \begin{pmatrix} \bar{Y} \\ \mathrm{Cov}(g_\lambda^{\mathrm{T}}, Y) + 2p\bar{Y} \\ \mathrm{Cov}(g_\lambda^{\mathrm{U}}, Y) + 2p\bar{Y} \end{pmatrix} \\
&= \frac{\mathrm{Cov}(g_\lambda^{\mathrm{T}}, Y) - \mathrm{Cov}(g_\lambda^{\mathrm{U}}, Y)}{2p(1-p)(1-F)} \\
&= \frac{\mathrm{Cov}(g_\lambda^{\mathrm{T}}, Y) - \mathrm{Cov}(g_\lambda^{\mathrm{U}}, Y)}{H_\lambda} \\
&= \frac{1}{H_\lambda}\left(2\sum_{l\in L}\left[D'_{\lambda l}(1-2c_{\lambda l}) + \tilde{D}'_{\lambda l}(2c_{\lambda l}-1)\right]\alpha_l^{\mathrm{d}} + \cancel{\mathrm{Cov}(g_\lambda, \epsilon) - \mathrm{Cov}(g_\lambda^{\mathrm{U}}, \epsilon)}\right) \\
&= \frac{2}{H_\lambda}\sum_{l\in L}\left(D'_{\lambda l} - \tilde{D}'_{\lambda l}\right)(1-2c_{\lambda l})\alpha_l^{\mathrm{d}}, \qquad \text{(S.26)}
\end{aligned}
$$

which is the same as Eq. (S.14).

We now calculate the estimate of the indirect effect from Eqs. (S.20), (S.24), and (S.25):

$$
\begin{aligned}
\hat{\alpha}_\lambda^{\mathrm{i}} = \hat{\alpha}_\lambda^{\mathrm{U}} &= \frac{\begin{pmatrix} -2p(1-F) & -2F & 1+F \end{pmatrix}}{2p(1-p)(1+3F)(1-F)} \begin{pmatrix} \bar{Y} \\ \mathrm{Cov}(g_\lambda^{\mathrm{T}}, Y) + 2p\bar{Y} \\ \mathrm{Cov}(g_\lambda^{\mathrm{U}}, Y) + 2p\bar{Y} \end{pmatrix} \\
&= \frac{(1+F)\mathrm{Cov}(g_\lambda^{\mathrm{U}}, Y) - 2F\mathrm{Cov}(g_\lambda^{\mathrm{T}}, Y)}{2p(1-p)(1-F)(1+3F)} \\
&= \frac{1}{H_\lambda(1+3F)}\left(2\sum_{l\in L}\left[D'_{\lambda l}[(1+3F)c_{\lambda l} - 2F] + \tilde{D}'_{\lambda l}[-(1+3F)c_{\lambda l} + 1 + F] + (1-F)\tilde{D}_{\lambda l}\right]\alpha_l^{\mathrm{d}}\right. \\
&\qquad \left. + 2(1-F)\sum_{l\in L}\left[D'_{\lambda l} + \tilde{D}'_{\lambda l} + 2\tilde{D}_{\lambda l}\right]\alpha_l^{\mathrm{i}} + (1+F)\mathrm{Cov}(g_\lambda^{\mathrm{U}}, \epsilon) - 2F\mathrm{Cov}(g_\lambda, \epsilon)\right). \qquad \text{(S.27)}
\end{aligned}
$$

This expression includes direct and indirect genetic confounds, as well as environmental confounding. It is easiest to interpret when $F = 0$, in which case

$$
\hat{\alpha}_\lambda^{\mathrm{i}} = \frac{1}{H_\lambda}\left(2\sum_{l\in L}\left[D'_{\lambda l}c_{\lambda l} + \tilde{D}'_{\lambda l}(1-c_{\lambda l}) + \tilde{D}_{\lambda l}\right]\alpha_l^{\mathrm{d}} + 2\sum_{l\in L}\left[D'_{\lambda l} + \tilde{D}'_{\lambda l} + 2\tilde{D}_{\lambda l}\right]\alpha_l^{\mathrm{i}} + \mathrm{Cov}(g_\lambda^{\mathrm{U}}, \epsilon)\right). \qquad \text{(S.28)}
$$

By our definition of L_{local}, for $l \in L_{\mathrm{local}}$, $c_{\lambda l} \approx 0$ and $F = 0 \Rightarrow \tilde{D}_{\lambda l} = \tilde{D}'_{\lambda l} = 0$, so Eq. (S.28) simplifies to

$$
\hat{\alpha}_\lambda^{\mathrm{i}} = \frac{2}{H_\lambda}\left(\sum_{l\in L_{\mathrm{local}}} D'_{\lambda l}\alpha_l^{\mathrm{i}} + \sum_{l\in L\setminus L_{\mathrm{local}}}\left[D'_{\lambda l}c_{\lambda l} + \tilde{D}'_{\lambda l}(1-c_{\lambda l}) + \tilde{D}_{\lambda l}\right]\alpha_l^{\mathrm{d}} + \sum_{l\in L\setminus L_{\mathrm{local}}}\left[D'_{\lambda l} + \tilde{D}'_{\lambda l} + 2\tilde{D}_{\lambda l}\right]\alpha_l^{\mathrm{i}} + \mathrm{Cov}(g_\lambda^{\mathrm{U}}, \epsilon)\right) \qquad \text{(S.29)}
$$

which is Eq. (9) in the Main Text.

S2 Polygenic scores and their phenotypic correlations

Suppose that we have estimated effect sizes $\hat{\alpha}_\lambda$ at a set of genotyped loci $\lambda \in \Lambda$ using a population GWAS for trait 1. For each individual, we can then compute a polygenic score:

$$PGS_1 = \sum_{\lambda \in \Lambda} g_\lambda \hat{\alpha}_\lambda^{\text{pop}}. \tag{S.30}$$

PGSs are often treated as predictions of individuals' genetic values for traits. In this regard, we might therefore be interested in the covariance across the population between the PGS for a trait and individuals' values for that trait: $\text{Cov}(PGS_1, Y_1)$. Additionally, if PGSs are treated as predictions of genetic values of traits, then we might be interested in how the PGS calculated for one trait covaries with the value of another trait: $\text{Cov}(PGS_1, Y_2)$. Such covariances might be informative of genetic correlations between traits, or pleiotropy of the alleles underlying genetic variation in the traits. We focus on the two-trait covariance, since it nests the single-trait covariance as a special case. If the total set of loci causally underlying variation in traits 1 and 2 is L, then the population covariance between the PGS for trait 1 and the value of trait 2 is

$$\begin{aligned}\text{Cov}\left(PGS_1, Y_2\right) &= \text{Cov}\left(\sum_{\lambda \in \Lambda} g_\lambda \hat{\alpha}_\lambda^{\text{pop}}, \sum_{l \in L} g_l \beta_l\right) \\ &= \text{Cov}\left(\sum_{\lambda \in \Lambda} \left(g_\lambda^{\text{m}} + g_\lambda^{\text{p}}\right) \hat{\alpha}_\lambda^{\text{pop}}, \sum_{l \in L} \left(g_l^{\text{m}} + g_l^{\text{p}}\right) \beta_l\right) \\ &= 2 \sum_{\lambda \in \Lambda} \sum_{l \in L} \left(D_{\lambda l} + \tilde{D}_{\lambda l}\right) \hat{\alpha}_\lambda^{\text{pop}} \beta_l. \end{aligned} \tag{S.31}$$

The effect-size estimates from the population GWAS for trait 1 are

$$\hat{\alpha}_\lambda^{\text{pop}} = \frac{2}{V_\lambda} \sum_{l' \in L} (D_{\lambda l'} + \tilde{D}_{\lambda l'}) \alpha_{l'} \approx \alpha_\lambda + \frac{2}{V_\lambda} \sum_{\substack{l' \in L \\ l' \neq \lambda}} (D_{\lambda l'} + \tilde{D}_{\lambda l'}) \alpha_{l'},$$

and so Eq. (S.31) is, in general,

$$\text{Cov}\left(PGS_1, Y_2\right) = \sum_{\lambda \in \Lambda} 2 p_\lambda (1 - p_\lambda) \alpha_\lambda \beta_\lambda + 2 \sum_{\lambda \in \Lambda} \sum_{\substack{l \in L \\ l \neq \lambda}} \left(D_{\lambda l} + \tilde{D}_{\lambda l}\right) \alpha_\lambda \beta_l \tag{S.32}$$

$$+ 4 \sum_{\lambda \in \Lambda} \sum_{\substack{l' \in L \\ l' \neq \lambda}} \sum_{\substack{l \in L \\ l \neq \lambda}} \frac{1}{V_\lambda} \left(D_{\lambda l'} + \tilde{D}_{\lambda l'}\right) \left(D_{\lambda l} + \tilde{D}_{\lambda l}\right) \alpha_{l'} \beta_l. \tag{S.33}$$

In a family-based study, we might instead be interested in the covariance between siblings' differences in the trait-1 population PGS and their differences in trait 2. We can write this covariance in our model

as

$$\begin{aligned}
\mathrm{Cov}\left(\Delta PGS_1, \Delta Y_2\right) &= \mathrm{Cov}\left(\sum_{\lambda\in\Lambda}\left(\Delta g_\lambda^{\mathrm{m}} + \Delta g_\lambda^{\mathrm{p}}\right)\hat{\alpha}_\lambda^{\mathrm{pop}}, \sum_{l\in L}\left(\Delta g_l^{\mathrm{m}} + \Delta g_l^{\mathrm{p}}\right)\beta_l\right) \\
&= \mathbb{E}\left[\left(\sum_{\lambda\in\Lambda}\left(\Delta g_\lambda^{\mathrm{m}} + \Delta g_\lambda^{\mathrm{p}}\right)\hat{\alpha}_\lambda^{\mathrm{pop}}\right)\left(\sum_{l\in L}\left(\Delta g_l^{\mathrm{m}} + \Delta g_l^{\mathrm{p}}\right)\beta_l\right)\right] \\
&= \sum_{\lambda\in\Lambda}\sum_{l\in L}\mathbb{E}\left[\left(\Delta g_\lambda^{\mathrm{m}} + \Delta g_\lambda^{\mathrm{p}}\right)\left(\Delta g_l^{\mathrm{m}} + \Delta g_l^{\mathrm{p}}\right)\hat{\alpha}_\lambda^{\mathrm{pop}}\beta_l\right] \\
&= \sum_{\lambda\in\Lambda}\sum_{l\in L}\left(\mathbb{E}\left[\Delta g_\lambda^{\mathrm{m}}\Delta g_l^{\mathrm{m}}\hat{\alpha}_\lambda^{\mathrm{pop}}\beta_l\right] + \mathbb{E}\left[\Delta g_\lambda^{\mathrm{p}}\Delta g_l^{\mathrm{p}}\hat{\alpha}_\lambda^{\mathrm{pop}}\beta_l\right]\right), \qquad \text{(S.34)}
\end{aligned}$$

since maternal and paternal transmission are conditionally independent. Focusing on maternal transmission, and writing $h_{\lambda l}^{\mathrm{c,m}}$ and $h_{\lambda l}^{\mathrm{r,m}}$ for the events that the mother is respectively a coupling and a repulsion heterozygote at loci λ and l, with $H_{\lambda l}^{\mathrm{coup}}$ and $H_{\lambda l}^{\mathrm{rep}}$ their associated probabilities (which are assumed to be the same for mothers and fathers),

$$\begin{aligned}
\mathbb{E}\left[\Delta g_\lambda^{\mathrm{m}}\Delta g_l^{\mathrm{m}}\hat{\alpha}_\lambda^{\mathrm{pop}}\beta_l\right] &= \mathbb{E}\left[\Delta g_\lambda^{\mathrm{m}}\Delta g_l^{\mathrm{m}}\hat{\alpha}_\lambda^{\mathrm{pop}}\beta_l \mid h_{\lambda l}^{\mathrm{c,m}}\right]H_{\lambda l}^{\mathrm{coup}} + \mathbb{E}\left[\Delta g_\lambda^{\mathrm{m}}\Delta g_l^{\mathrm{m}}\hat{\alpha}_\lambda^{\mathrm{pop}}\beta_l \mid h_{\lambda l}^{\mathrm{r,m}}\right]H_{\lambda l}^{\mathrm{rep}} \\
&= \left(\mathbb{E}\left[\Delta g_\lambda^{\mathrm{m}}\Delta g_l^{\mathrm{m}} \mid h_{\lambda l}^{\mathrm{c,m}}\right]H_{\lambda l}^{\mathrm{coup}} + \mathbb{E}\left[\Delta g_\lambda^{\mathrm{m}}\Delta g_l^{\mathrm{m}} \mid h_{\lambda l}^{\mathrm{r,m}}\right]H_{\lambda l}^{\mathrm{rep}}\right)\hat{\alpha}_\lambda^{\mathrm{pop}}\beta_l \\
&= \left(\frac{1}{2} - c_{\lambda l}^{♀}\right)\left(H_{\lambda l}^{\mathrm{coup}} - H_{\lambda l}^{\mathrm{rep}}\right)\hat{\alpha}_\lambda^{\mathrm{pop}}\beta_l \\
&= \left(1 - 2c_{\lambda l}^{♀}\right)\left(D'_{\lambda l} - \tilde{D}'_{\lambda l}\right)\hat{\alpha}_\lambda^{\mathrm{pop}}\beta_l,
\end{aligned}$$

with $D'_{\lambda l}$ and $\tilde{D}'_{\lambda l}$ measured in the parents. Similarly,

$$\mathbb{E}\left[\Delta g_\lambda^{\mathrm{p}}\Delta g_l^{\mathrm{p}}\hat{\alpha}_\lambda^{\mathrm{pop}}\beta_l\right] = \left(1 - 2c_{\lambda l}^{♂}\right)\left(D'_{\lambda l} - \tilde{D}'_{\lambda l}\right)\hat{\alpha}_\lambda^{\mathrm{pop}}\beta_l,$$

and so Eq. (S.34) becomes

$$\mathrm{Cov}\left(\Delta PGS_1, \Delta Y_2\right) = 2\sum_{\lambda\in\Lambda}\sum_{l\in L}\left(1 - 2c_{\lambda l}\right)\left(D'_{\lambda l} - \tilde{D}'_{\lambda l}\right)\hat{\alpha}_\lambda^{\mathrm{pop}}\beta_l, \qquad \text{(S.35)}$$

where $c_{\lambda l}$ is the sex-averaged recombination fraction between λ and l.

Before we substitute the population GWAS estimates $\hat{\alpha}_\lambda^{\mathrm{pop}}$ into Eq. (S.35), it is worth considering what value this expression would take if direct genetic effects were correctly estimated at every study locus, $\hat{\alpha}_\lambda^{\mathrm{pop}} = \alpha_\lambda$. In this case, Eq. (S.35) becomes

$$\begin{aligned}
\mathrm{Cov}\left(\Delta PGS_1, \Delta Y_2\right) &= 2\sum_{\lambda\in\Lambda}\sum_{l\in L}\left(1 - 2c_{\lambda l}\right)\left(D_{\lambda l} - \tilde{D}_{\lambda l}\right)\alpha_\lambda\beta_l \\
&= \sum_{\lambda\in\Lambda}2p_\lambda(1 - p_\lambda)\alpha_\lambda\beta_\lambda + 2\sum_{\lambda\in\Lambda}\sum_{\substack{l\in L\\ l\neq\lambda}}\left(1 - 2c_{\lambda l}\right)\left(D'_{\lambda l} - \tilde{D}'_{\lambda l}\right)\alpha_\lambda\beta_l. \qquad \text{(S.36)}
\end{aligned}$$

If the two traits are distinct, then the first term in Eq. (S.36) is the genic covariance of traits 1 and 2 across the set of study loci (more precisely, tagged locally by the study loci), and reflects systematic pleiotropy at these loci; this term would, for example, be positive if alleles tend to have same-direction

effects on traits 1 and 2. If we were studying only one trait, then $\alpha_\lambda = \beta_\lambda$, and the first term would be the genic variance of the trait across study loci, $\sum_{\lambda \in \Lambda} 2p_\lambda(1-p_\lambda)\alpha_\lambda^2$. The second term in Eq. (S.36) is an effect of linkage disequilibria between study loci and the loci that are causal for trait 2; these LDs are absorbed by the PGS because the PGS is a sum across loci. In the absence of such LDs, or in cases where the cis- and trans-LDs are equal so that $D'_{\lambda l} - \tilde{D}'_{\lambda l} = 0$, Eq. (S.36) would equal the genic variance in the single-trait case and the genic covariance in the two-trait case.

The effect-size estimates from a population GWAS are in fact

$$\hat{\alpha}_\lambda^{\text{pop}} = \frac{2}{V_\lambda} \sum_{l' \in L} (D_{\lambda l'} + \tilde{D}_{\lambda l'})\alpha_{l'} \approx \alpha_\lambda + \frac{2}{V_\lambda} \sum_{\substack{l' \in L \\ l' \neq \lambda}} (D_{\lambda l'} + \tilde{D}_{\lambda l'})\alpha_{l'},$$

$D_{\lambda l'}$ and $\tilde{D}_{\lambda l'}$ are measured in the sample. We assume these to be equal to the values in parents in the family-based GWAS, $D'_{\lambda l}$ and $\tilde{D}'_{\lambda l}$, and so the value taken by Eq. (S.35) is

$$\begin{aligned}
\text{Cov}\left(\Delta PGS_1, \Delta Y_2\right) &= 2 \sum_{\lambda \in \Lambda} \sum_{l \in L} (1 - 2c_{\lambda l}) \left(D_{\lambda l} - \tilde{D}_{\lambda l}\right) \hat{\alpha}_\lambda^{\text{pop}} \beta_l \\
&= 2 \sum_{\lambda \in \Lambda} \sum_{l \in L} (1 - 2c_{\lambda l}) \left(D_{\lambda l} - \tilde{D}_{\lambda l}\right) \left(\alpha_\lambda + \frac{2}{V_\lambda} \sum_{\substack{l' \in L \\ l' \neq \lambda}} \left(D_{\lambda l'} + \tilde{D}_{\lambda l'}\right) \alpha_{l'}\right) \beta_l \\
&= \underbrace{\sum_{\lambda \in \Lambda} 2p_\lambda(1-p_\lambda)\alpha_\lambda\beta_\lambda}_{\text{pleiotropy}} \ + \ \underbrace{2 \sum_{\lambda \in \Lambda} \sum_{\substack{l \in L \\ l \neq \lambda}} (1 - 2c_{\lambda l}) \left(D_{\lambda l} - \tilde{D}_{\lambda l}\right) \alpha_\lambda \beta_l}_{\substack{\text{covariance from LD absorbed by PGS} \\ \text{because it is a sum across loci}}} \\
&\quad + \ \underbrace{4 \sum_{\lambda \in \Lambda} \sum_{\substack{l \in L \\ l \neq \lambda}} (1 - 2c_{\lambda l}) \left(D_{\lambda l}^2 - \tilde{D}_{\lambda l}^2\right) \alpha_l \beta_l / V_\lambda}_{\substack{\text{covariance from LD absorbed by PGS} \\ \text{because effect-size estimates absorb LD}}} \\
&\quad + \ \underbrace{4 \sum_{\lambda \in \Lambda} \sum_{\substack{l \in L \\ l \neq \lambda}} \sum_{\substack{l' \in L \\ l' \neq \lambda, l}} (1 - 2c_{\lambda l}) \left(D_{\lambda l'} + \tilde{D}_{\lambda l'}\right) \alpha_{l'} \left(D_{\lambda l} - \tilde{D}_{\lambda l}\right) \beta_l / V_\lambda}_{\substack{\text{covariance from systematic LD between variants} \\ \text{with same directional effect on trait}}} .
\end{aligned} \tag{S.37}$$

In the absence of genetic confounding ($D_{\lambda l} = \tilde{D}_{\lambda l} = 0$) or, more generally, if genetic stratification is such that the cis- and trans-LDs are equal ($D_{\lambda l} - \tilde{D}_{\lambda l} = 0$), then Eq. (S.37) simplifies to the SNP-tagged genic covariance between traits 1 and 2:

$$\text{Cov}\left(\Delta PGS_1, \Delta Y_2\right) = \sum_{\lambda \in \Lambda} 2p_\lambda(1-p_\lambda)\alpha_\lambda\beta_\lambda. \tag{S.38}$$

If traits 1 and 2 are the same, then this is simply the SNP-tagged genic variance of the trait: $\text{Cov}\left(\Delta PGS, \Delta Y\right) = \sum_{\lambda \in \Lambda} 2p_\lambda(1-p_\lambda)\alpha_\lambda^2$.

Eq. (S.37) simplifies somewhat if we focus on a single trait ($\alpha_l = \beta_l$) and assume that there is no

trans-LD ($\tilde{D}_{\lambda l} = 0$); in this case,

$$\begin{aligned}\mathrm{Cov}\left(\Delta PGS, \Delta Y\right) = & \underbrace{\sum_{\lambda\in\Lambda} 2p_\lambda(1-p_\lambda)\alpha_\lambda^2}_{\text{SNP-tagged genic variance}} \quad + \quad \underbrace{2\sum_{\lambda\in\Lambda}\sum_{\substack{l\in L\\ l\neq\lambda}}(1-2c_{\lambda l})D_{\lambda l}\alpha_\lambda\alpha_l}_{\substack{\text{variance from LD absorbed by PGS}\\ \text{because it is a sum across loci}}} \\ & + \quad \underbrace{4\sum_{\lambda\in\Lambda}\sum_{\substack{l\in L\\ l\neq\lambda}}(1-2c_{\lambda l})D_{\lambda l}^2\alpha_l^2/V_\lambda}_{\substack{\text{variance from LD absorbed by PGS}\\ \text{because effect-size estimates absorb LD}}} \quad + \quad \underbrace{4\sum_{\lambda\in\Lambda}\sum_{\substack{l\in L\\ l\neq\lambda}}\sum_{\substack{l'\in L\\ l'\neq\lambda,l}}(1-2c_{\lambda l})D_{\lambda l}\alpha_l D_{\lambda l'}\alpha_{l'}/V_\lambda}_{\substack{\text{variance from systematic LD between variants}\\ \text{with same directional effect on trait}}}\,.\end{aligned} \tag{S.39}$$

S3 Sources of genetic confounding

The calculations above reveal that genetic confounds in GWAS designs can depend on long-range LD in the sample and among parents of the sample. Here, we consider several possible sources of long-range LD.

S3.1 Assortative mating

If there is a constant correlation among mates for their values of two traits, then a genetic equilibrium will eventually be achieved. In this equilibrium, for any pair of loci l and l', the trans-LD $\tilde{D}_{ll'}$ will be constant. Call this constant value $D^*_{ll'}$, and suppose that the recombination fraction between the loci is $c_{ll'}$. With $\tilde{D}_{ll'}$ constant across generations, the balance of its conversion into cis-LD (at rate $c_{ll'}$ per generation) and the destruction of cis-LD by recombination (at rate $c_{ll'}$ per generation) will result in an equilibrium level of cis-LD equal to the degree of trans-LD: $D_{ll'} = \tilde{D}_{ll'} = D^*_{ll'}$ (e.g., [2]).

The value of $D^*_{ll'}$ will, in general, depend in a complicated way on the strength of effects of l and l' on the traits upon which assortative mating is based and on the linkage relations of these loci to one another and to other causal loci. However, while it is therefore difficult to calculate the individual equilibrium LD terms $D^*_{ll'}$, we can in some cases calculate weighted sums of these terms across locus pairs. Note that the calculations below assume that the number of loci influencing the traits in question is large.

Let the set of loci that influence one or both traits be L, and let α_l be the effect size of the focal variant at locus l on trait 1 and β_l its effect on trait 2 (the analyses below also apply to same-trait assortative mating, setting $\alpha_l = \beta_l$). Recall the notation $g_l^{\mathrm{m,mat}}$ and $g_l^{\mathrm{m,pat}}$ for a mother's maternally and paternally inherited genotype at locus l, with $g_l^{\mathrm{f,mat}}$ and $g_l^{\mathrm{f,pat}}$ a father's analogs. The mother's breeding value for trait 1 is

$$G_1^{\mathrm{m}} = \sum_{l\in L} g_l^{\mathrm{m}}\alpha_l = \sum_{l\in L}\left(g_l^{\mathrm{m,mat}} + g_l^{\mathrm{m,pat}}\right)\alpha_l = \sum_{l\in L} g_l^{\mathrm{m,mat}}\alpha_l + \sum_{l\in L} g_l^{\mathrm{m,pat}}\alpha_l = G_1^{\mathrm{m,mat}} + G_1^{\mathrm{m,pat}},$$

and, similarly, her breeding value for trait 2 is

$$G_2^{\mathrm{m}} = \sum_{l\in L} g_l^{\mathrm{m,mat}}\beta_l + \sum_{l\in L} g_l^{\mathrm{m,pat}}\beta_l = G_2^{\mathrm{m,mat}} + G_2^{\mathrm{m,pat}}.$$

The father's breeding values for the two traits are

$$G_1^{\mathrm{f}} = \sum_{l\in L} g_l^{\mathrm{f,mat}}\alpha_l + \sum_{l\in L} g_l^{\mathrm{f,pat}}\alpha_l = G_1^{\mathrm{f,mat}} + G_1^{\mathrm{f,pat}}$$

and

$$G_2^{\mathrm{f}} = \sum_{l \in L} g_l^{\mathrm{f,mat}} \beta_l + \sum_{l \in L} g_l^{\mathrm{f,pat}} \beta_l = G_2^{\mathrm{f,mat}} + G_2^{\mathrm{f,pat}}.$$

We assume that individual trait values equal the breeding values plus environmental disturbances that are uncorrelated with the breeding values:

$$Y_1^{\mathrm{m}} = G_1^{\mathrm{m}} + \epsilon_1^{\mathrm{m}}; \;\; Y_2^{\mathrm{m}} = G_2^{\mathrm{m}} + \epsilon_2^{\mathrm{m}}; \;\; Y_1^{\mathrm{f}} = G_1^{\mathrm{f}} + \epsilon_1^{\mathrm{f}}; \;\; Y_2^{\mathrm{f}} = G_2^{\mathrm{f}} + \epsilon_2^{\mathrm{f}};$$

where

$$\mathrm{Var}(\epsilon_1^{\mathrm{m}}) = \mathrm{Var}(\epsilon_1^{\mathrm{f}}) = V_E^1, \;\; \mathrm{Var}(\epsilon_2^{\mathrm{m}}) = \mathrm{Var}(\epsilon_2^{\mathrm{f}}) = V_E^2,$$

and

$$\mathrm{Cov}(\epsilon_i^{\mathrm{m}}, G_i^{\mathrm{m}}) = \mathrm{Cov}(\epsilon_i^{\mathrm{f}}, G_i^{\mathrm{f}}) = 0 \text{ for } i \in \{1, 2\}.$$

S3.1.1 Same-trait assortative mating, or cross-trait assortative mating that is symmetric with respect to sex

We first consider the case where the strength of assortative mating between two traits, as measured by their correlation coefficient across mating pairs, is equal in the female-male and male-female directions. Notice that this scenario covers same-trait assortative mating. In the case of cross-trait assortative mating, it could occur if assortative mating arises by mechanisms other than direct female (or male) mating preferences.

We assume that there is a constant correlation ρ among mating pairs for their phenotypic values of traits 1 and 2. In equilibrium, this will translate to a constant correlation ρ_G between their breeding values as well (e.g., [3]). To calculate ρ_G, we first note that, because assortative mating is based on phenotypic values and not breeding values per se, if we know the phenotypes of a pair of mates, we obtain no further information about the similarity of their breeding values; that is,

$$\mathrm{Cov}\left(G_1^{\mathrm{m}}, G_2^{\mathrm{f}} \mid \left\{Y_1^{\mathrm{m}}, Y_2^{\mathrm{f}}\right\}\right) = \mathrm{Cov}\left(G_2^{\mathrm{m}}, G_1^{\mathrm{f}} \mid \left\{Y_2^{\mathrm{m}}, Y_1^{\mathrm{f}}\right\}\right) = 0. \tag{S.40}$$

For the same reason, if we know the phenotypic values of two mates, then the trait-2 value of the male does not offer any information on the female's trait-1 breeding value beyond that already offered by the female's trait-1 phenotype, and vice versa; that is,

$$\begin{aligned} &\mathbb{E}\left[G_1^{\mathrm{m}} \mid \left\{Y_1^{\mathrm{m}}, Y_2^{\mathrm{f}}\right\}\right] = \mathbb{E}\left[G_1^{\mathrm{m}} \mid Y_1^{\mathrm{m}}\right]; \;\; \mathbb{E}\left[G_2^{\mathrm{f}} \mid \left\{Y_1^{\mathrm{m}}, Y_2^{\mathrm{f}}\right\}\right] = \mathbb{E}\left[G_2^{\mathrm{f}} \mid Y_2^{\mathrm{f}}\right]; \\ &\mathbb{E}\left[G_2^{\mathrm{m}} \mid \left\{Y_2^{\mathrm{m}}, Y_1^{\mathrm{f}}\right\}\right] = \mathbb{E}\left[G_2^{\mathrm{m}} \mid Y_2^{\mathrm{m}}\right]; \;\; \mathbb{E}\left[G_1^{\mathrm{f}} \mid \left\{Y_2^{\mathrm{m}}, Y_1^{\mathrm{f}}\right\}\right] = \mathbb{E}\left[G_1^{\mathrm{f}} \mid Y_1^{\mathrm{f}}\right]. \end{aligned} \tag{S.41}$$

If Y_1 and G_1, and similarly Y_2 and G_2, are bivariate normal, then

$$\mathbb{E}\left[G_1 \mid Y_1\right] = \mathbb{E}\left[G_1\right] + h_1^2 \left(Y_1 - \mathbb{E}\left[Y_1\right]\right) \quad \text{and} \quad \mathbb{E}\left[G_2 \mid Y_2\right] = \mathbb{E}\left[G_2\right] + h_2^2 \left(Y_2 - \mathbb{E}\left[Y_2\right]\right) \tag{S.42}$$

where h_1^2 and h_2^2 are the heritabilities of traits 1 and 2, respectively.

From the law of total covariance,

$$
\begin{aligned}
\mathrm{Cov}\left(G_1^{\mathrm{m}}, G_2^{\mathrm{f}}\right) &= \mathrm{Cov}_{\left\{Y_1^{\mathrm{m}}, Y_2^{\mathrm{f}}\right\}}\left(\mathbb{E}\left[G_1^{\mathrm{m}} \mid \left\{Y_1^{\mathrm{m}}, Y_2^{\mathrm{f}}\right\}\right],\ \mathbb{E}\left[G_2^{\mathrm{f}} \mid \left\{Y_1^{\mathrm{m}}, Y_2^{\mathrm{f}}\right\}\right]\right) \\
&\quad + \mathbb{E}_{\left\{Y_1^{\mathrm{m}}, Y_2^{\mathrm{f}}\right\}}\left[\mathrm{Cov}\left(G_1^{\mathrm{m}}, G_2^{\mathrm{f}} \mid \left\{Y_1^{\mathrm{m}}, Y_2^{\mathrm{f}}\right\}\right)\right] \\
&= \mathrm{Cov}_{\left\{Y_1^{\mathrm{m}}, Y_2^{\mathrm{f}}\right\}}\left(\mathbb{E}\left[G_1^{\mathrm{m}} \mid Y_1^{\mathrm{m}}\right],\ \mathbb{E}\left[G_2^{\mathrm{f}} \mid Y_2^{\mathrm{f}}\right]\right) && \text{[from Eqs. S.40 and S.41]} \\
&= \mathrm{Cov}\left(h_1^2 Y_1^{\mathrm{m}}, h_2^2 Y_2^{\mathrm{f}}\right) && \text{[from Eq. S.42]} \\
&= h_1^2 h_2^2 \mathrm{Cov}\left(Y_1^{\mathrm{m}}, Y_2^{\mathrm{f}}\right). && \text{(S.43)}
\end{aligned}
$$

Similarly, $\mathrm{Cov}\left(G_2^{\mathrm{m}}, G_1^{\mathrm{f}}\right) = h_1^2 h_2^2 \mathrm{Cov}\left(Y_2^{\mathrm{m}}, Y_1^{\mathrm{f}}\right)$.

Let V^1 and V^2 be the phenotypic variances of traits 1 and 2, and V_G^1 and V_G^2 their additive genetic variances, assumed to be the same across the sexes. Given the calculations above, the correlation among mates for their breeding values of traits 1 and 2, ρ_G, can be written

$$
\begin{aligned}
\rho_G &= \frac{\frac{1}{2}\left[\mathrm{Cov}\left(G_1^{\mathrm{m}}, G_2^{\mathrm{f}}\right) + \mathrm{Cov}\left(G_2^{\mathrm{m}}, G_1^{\mathrm{f}}\right)\right]}{\sqrt{V_G^1 V_G^2}} && \text{(S.44)} \\
&= \frac{\frac{h_1^2 h_2^2}{2}\left[\mathrm{Cov}\left(Y_1^{\mathrm{m}}, Y_2^{\mathrm{f}}\right) + \mathrm{Cov}\left(Y_2^{\mathrm{m}}, Y_1^{\mathrm{f}}\right)\right]}{\sqrt{h_1^2 V^1 h_2^2 V^2}} \\
&= h_1 h_2 \frac{\frac{1}{2}\left[\mathrm{Cov}\left(Y_1^{\mathrm{m}}, Y_2^{\mathrm{f}}\right) + \mathrm{Cov}\left(Y_2^{\mathrm{m}}, Y_1^{\mathrm{f}}\right)\right]}{\sqrt{V^1 V^2}} = h_1 h_2 \rho. && \text{(S.45)}
\end{aligned}
$$

When traits 1 and 2 are the same, we have $\rho_G = h^2\rho$, a standard result (e.g., [3, 4]).

Expanding the numerator of Eq. (S.44),

$$
\begin{aligned}
\frac{1}{2}\left[\mathrm{Cov}\left(G_1^{\mathrm{m}}, G_2^{\mathrm{f}}\right) + \mathrm{Cov}\left(G_2^{\mathrm{m}}, G_1^{\mathrm{f}}\right)\right] &= \frac{1}{2}\left[\mathrm{Cov}\left(G_1^{\mathrm{m,mat}} + G_1^{\mathrm{m,pat}}, G_2^{\mathrm{f,mat}} + G_2^{\mathrm{f,pat}}\right)\right. \\
&\qquad \left. + \mathrm{Cov}\left(G_2^{\mathrm{m,mat}} + G_2^{\mathrm{m,pat}}, G_1^{\mathrm{f,mat}} + G_1^{\mathrm{f,pat}}\right)\right] \\
= \frac{1}{2}\left[\mathrm{Cov}\left(G_1^{\mathrm{m,mat}}, G_2^{\mathrm{f,mat}}\right) + \mathrm{Cov}\left(G_1^{\mathrm{m,mat}}, G_2^{\mathrm{f,pat}}\right)\right] &+ \frac{1}{2}\left[\mathrm{Cov}\left(G_1^{\mathrm{m,pat}}, G_2^{\mathrm{f,mat}}\right) + \mathrm{Cov}\left(G_1^{\mathrm{m,pat}}, G_2^{\mathrm{f,pat}}\right)\right] \\
+ \frac{1}{2}\left[\mathrm{Cov}\left(G_2^{\mathrm{m,mat}}, G_1^{\mathrm{f,mat}}\right) + \mathrm{Cov}\left(G_2^{\mathrm{m,mat}}, G_1^{\mathrm{f,pat}}\right)\right] &+ \frac{1}{2}\left[\mathrm{Cov}\left(G_2^{\mathrm{m,pat}}, G_1^{\mathrm{f,mat}}\right) + \mathrm{Cov}\left(G_2^{\mathrm{m,pat}}, G_1^{\mathrm{f,pat}}\right)\right] \\
= \frac{1}{2}\left[\mathrm{Cov}\left(G_1^{\mathrm{m,mat}}, G_2^{\mathrm{f,mat}}\right) + \mathrm{Cov}\left(G_2^{\mathrm{m,mat}}, G_1^{\mathrm{f,mat}}\right)\right] &+ \frac{1}{2}\left[\mathrm{Cov}\left(G_1^{\mathrm{m,mat}}, G_2^{\mathrm{f,pat}}\right) + \mathrm{Cov}\left(G_2^{\mathrm{m,mat}}, G_1^{\mathrm{f,pat}}\right)\right] \\
+ \frac{1}{2}\left[\mathrm{Cov}\left(G_1^{\mathrm{m,pat}}, G_2^{\mathrm{f,mat}}\right) + \mathrm{Cov}\left(G_2^{\mathrm{m,pat}}, G_1^{\mathrm{f,mat}}\right)\right] &+ \frac{1}{2}\left[\mathrm{Cov}\left(G_1^{\mathrm{m,pat}}, G_2^{\mathrm{f,pat}}\right) + \mathrm{Cov}\left(G_2^{\mathrm{m,pat}}, G_1^{\mathrm{f,pat}}\right)\right].
\end{aligned}
\tag{S.46}
$$

But

$$\frac{1}{2}\left[\mathrm{Cov}\left(G_1^{\mathrm{m,mat}}, G_2^{\mathrm{f,mat}}\right) + \mathrm{Cov}\left(G_2^{\mathrm{m,mat}}, G_1^{\mathrm{f,mat}}\right)\right] = \frac{1}{2}\left[\mathrm{Cov}\left(\sum_{l\in L} g_l^{\mathrm{m,mat}}\alpha_l, \sum_{l'\in L} g_{l'}^{\mathrm{f,mat}}\beta_{l'}\right)\right.$$
$$\left. +\mathrm{Cov}\left(\sum_{l\in L} g_l^{\mathrm{m,mat}}\beta_l, \sum_{l'\in L} g_{l'}^{\mathrm{f,mat}}\alpha_{l'}\right)\right]$$
$$= \frac{1}{2}\left[\sum_{l\in L}\sum_{l'\in L}\mathrm{Cov}\left(g_l^{\mathrm{m,mat}}, g_{l'}^{\mathrm{f,mat}}\right)\alpha_l\beta_{l'} + \sum_{l\in L}\sum_{l'\in L}\mathrm{Cov}\left(g_l^{\mathrm{m,mat}}, g_{l'}^{\mathrm{f,mat}}\right)\alpha_{l'}\beta_l\right]$$
$$= \frac{1}{2}\left[\sum_{l\in L}\sum_{l'\in L}\mathrm{Cov}\left(g_l^{\mathrm{m,mat}}, g_{l'}^{\mathrm{f,mat}}\right)\alpha_l\beta_{l'} + \sum_{l\in L}\sum_{l'\in L}\mathrm{Cov}\left(g_{l'}^{\mathrm{m,mat}}, g_{l}^{\mathrm{f,mat}}\right)\alpha_l\beta_{l'}\right]$$
$$= \sum_{l\in L}\sum_{l'\in L}\frac{1}{2}\left[\mathrm{Cov}\left(g_l^{\mathrm{m,mat}}, g_{l'}^{\mathrm{f,mat}}\right) + \mathrm{Cov}\left(g_{l'}^{\mathrm{m,mat}}, g_{l}^{\mathrm{f,mat}}\right)\right]\alpha_l\beta_{l'}$$
$$= \sum_{l\in L}\sum_{l'\in L}\tilde{D}_{ll'}\alpha_l\beta_{l'},$$

since grandmaternal and grandpaternal alleles are transmitted to the offspring with equal probability, independently across maternal and paternal transmission. The three additional terms in Eq. (S.46) likewise each amount to $\sum_{l\in L}\sum_{l'\in L}\tilde{D}_{ll'}\alpha_l\beta_{l'}$, and so

$$\frac{1}{2}\left[\mathrm{Cov}\left(G_1^{\mathrm{m}}, G_2^{\mathrm{f}}\right) + \mathrm{Cov}\left(G_2^{\mathrm{m}}, G_1^{\mathrm{f}}\right)\right] = 4\sum_{l\in L}\sum_{l'\in L}\tilde{D}_{ll'}\alpha_l\beta_{l'}. \tag{S.47}$$

Noting that the trans-covariance at a given locus $\tilde{D}_{ll} = p_l(1-p_l)\tilde{r}_{ll}$, where $\tilde{r}_{ll}$ is the within-locus correlation (equal to the inbreeding coefficient at the locus), we can split Eq. (S.47) into within- and between-locus terms:

$$\frac{1}{2}\left[\mathrm{Cov}\left(G_1^{\mathrm{m}}, G_2^{\mathrm{f}}\right) + \mathrm{Cov}\left(G_2^{\mathrm{m}}, G_1^{\mathrm{f}}\right)\right] = 4\sum_{l\in L} p_l(1-p_l)\tilde{r}_{ll}\alpha_l\beta_l + 4\sum_{l\in L}\sum_{\substack{l'\in L\\ l'\neq l}}\tilde{D}_{ll'}\alpha_l\beta_{l'}. \tag{S.48}$$

In the denominator of Eq. (S.44),

$$V_G^1 = \mathrm{Var}\left(G_1^{\mathrm{m}}\right) = \mathrm{Var}\left(G_1^{\mathrm{m,mat}} + G_1^{\mathrm{m,pat}}\right) = \mathrm{Var}\left(G_1^{\mathrm{m,mat}}\right) + \mathrm{Var}\left(G_1^{\mathrm{m,pat}}\right) + 2\mathrm{Cov}\left(G_1^{\mathrm{m,mat}}, G_1^{\mathrm{m,pat}}\right), \tag{S.49}$$

Expanding the first term,

$$\mathrm{Var}\left(G_1^{\mathrm{m,mat}}\right) = \mathrm{Var}\left(\sum_{l\in L} g_l^{\mathrm{m,mat}}\alpha_l\right) = \sum_{l\in L}\mathrm{Var}\left(g_l^{\mathrm{m,mat}}\right)\alpha_l^2 + \sum_{l\in L}\sum_{\substack{l'\in L\\ l'\neq l}}\mathrm{Cov}\left(g_l^{\mathrm{m,mat}}, g_{l'}^{\mathrm{m,mat}}\right)\alpha_l\alpha_{l'}$$
$$= \sum_{l\in L} p_l(1-p_l)\alpha_l^2 + \sum_{l\in L}\sum_{\substack{l'\in L\\ l'\neq l}} D'_{ll'}\alpha_l\alpha_{l'}.$$

Similarly, the second term is

$$\mathrm{Var}\left(G_1^{\mathrm{m,pat}}\right) = \sum_{l\in L} p_l(1-p_l)\alpha_l^2 + \sum_{l\in L}\sum_{\substack{l'\in L\\ l'\neq l}} D'_{ll'}\alpha_l\alpha_{l'}.$$

The third, covariance term in Eq. (S.49) is

$$\begin{aligned}\operatorname{Cov}\left(G_1^{\mathrm{m,mat}}, G_1^{\mathrm{m,pat}}\right) &= \operatorname{Cov}\left(\sum_{l\in L} g_l^{\mathrm{m,mat}}\alpha_l, \sum_{l'\in L} g_{l'}^{\mathrm{m,pat}}\alpha_{l'}\right) = \sum_{l\in L}\sum_{l'\in L}\operatorname{Cov}\left(g_l^{\mathrm{m,mat}}, g_{l'}^{\mathrm{m,pat}}\right)\alpha_l\alpha_{l'} \\ &= \sum_{l\in L}\sum_{l'\in L}\frac{1}{2}\left[\operatorname{Cov}\left(g_l^{\mathrm{m,mat}}, g_{l'}^{\mathrm{m,pat}}\right) + \operatorname{Cov}\left(g_{l'}^{\mathrm{m,mat}}, g_l^{\mathrm{m,pat}}\right)\right]\alpha_l\alpha_{l'} \\ &= \sum_{l\in L}\sum_{l'\in L}\tilde{D}'_{ll'}\alpha_l\alpha_{l'} = \sum_{l\in L} p_l(1-p_l)\tilde{r}'_{ll}\alpha_l^2 + \sum_{l\in L}\sum_{\substack{l'\in L\\ l'\neq l}}\tilde{D}'_{ll'}\alpha_l\alpha_{l'}.\end{aligned}$$

Putting these together in Eq. (S.49),

$$V_G^1 = \operatorname{Var}\left(G_1^{\mathrm{m}}\right) = 2\sum_{l\in L} p_l(1-p_l)\left(1+\tilde{r}'_{ll}\right)\alpha_l^2 + 2\sum_{l\in L}\sum_{\substack{l'\in L\\ l'\neq l}}\left(D'_{ll'} + \tilde{D}'_{ll'}\right)\alpha_l\alpha_{l'}.$$

Similarly,

$$V_G^2 = \operatorname{Var}\left(G_2^{\mathrm{m}}\right) = 2\sum_{l\in L} p_l(1-p_l)\left(1+\tilde{r}'_{ll}\right)\beta_l^2 + 2\sum_{l\in L}\sum_{\substack{l'\in L\\ l'\neq l}}\left(D'_{ll'} + \tilde{D}'_{ll'}\right)\beta_l\beta_{l'}.$$

In equilibrium, $D'_{ll'} = \tilde{D}'_{ll'} = \tilde{D}_{ll'} = D^*_{ll'}$ for $l \neq l'$, and $\tilde{r}'_{ll} = \tilde{r}_{ll} = \tilde{r}^*_{ll}$, so

$$\frac{1}{2}\left[\operatorname{Cov}\left(G_1^{\mathrm{m}}, G_2^{\mathrm{f}}\right) + \operatorname{Cov}\left(G_2^{\mathrm{m}}, G_1^{\mathrm{f}}\right)\right] = 4\sum_{l\in L} p_l(1-p_l)r^*_{ll}\alpha_l\beta_l + 4\sum_{l\in L}\sum_{\substack{l'\in L\\ l'\neq l}} D^*_{ll'}\alpha_l\beta_{l'}, \tag{S.50}$$

$$V_G^1 = 2\sum_{l\in L} p_l(1-p_l)\left(1+\tilde{r}^*_{ll}\right)\alpha_l^2 + 4\sum_{l\in L}\sum_{\substack{l'\in L\\ l'\neq l}} D^*_{ll'}\alpha_l\alpha_{l'} + V_E^1 \approx V_g^1 + 4\sum_{l\in L}\sum_{\substack{l'\in L\\ l'\neq l}} D^*_{ll'}\alpha_l\alpha_{l'}, \tag{S.51}$$

$$V_G^2 = 2\sum_{l\in L} p_l(1-p_l)\left(1+\tilde{r}^*_{ll}\right)\beta_l^2 + 4\sum_{l\in L}\sum_{\substack{l'\in L\\ l'\neq l}} D^*_{ll'}\beta_l\beta_{l'} + V_E^2 \approx V_g^2 + 4\sum_{l\in L}\sum_{\substack{l'\in L\\ l'\neq l}} D^*_{ll'}\beta_l\beta_{l'}, \tag{S.52}$$

where V_g^1 and V_g^2 are the genic variances of traits 1 and 2, and the approximations come from the fact that, under assortative mating for a polygenic trait, the sum of the $\sim|L|^2$ cross-locus trans-LD terms $\tilde{D}^*_{ll'}$ dominates the sum of the $|L|$ within-locus trans-LD terms $\tilde{D}^*_{ll} = p_l(1-p_l)\tilde{r}^*_{ll}$ [5, Ch. 4]. Eq. (S.44) in equilibrium is therefore

$$\begin{aligned}\rho_G &= \frac{4\sum_{l\in L} p_l(1-p_l)\tilde{r}^*_{ll}\alpha_l\beta_l + 4\sum_{l\in L}\sum_{\substack{l'\in L\\ l'\neq l}} D^*_{ll'}\alpha_l\beta_{l'}}{\sqrt{V_G^1 V_G^2}} \\ &\approx \frac{4\sum_{l\in L}\sum_{\substack{l'\in L\\ l'\neq l}} D^*_{ll'}\alpha_l\beta_{l'}}{\sqrt{\left(V_g^1 + 4\sum_{l\in L}\sum_{\substack{l'\in L\\ l'\neq l}} D^*_{ll'}\alpha_l\alpha_{l'}\right)\left(V_g^2 + 4\sum_{l\in L}\sum_{\substack{l'\in L\\ l'\neq l}} D^*_{ll'}\beta_l\beta_{l'}\right)}}.\end{aligned} \tag{S.53}$$

We now consider some special cases.

Same-trait assortative mating with equal effect sizes. In the case of same-trait assortative mating, $\alpha_l = \beta_l$, so Eq. (S.53) simplifies to

$$\rho_G = \frac{4 \sum_{l \in L} \sum_{\substack{l' \in L \\ l' \neq l}} D^*_{ll'} \alpha_l \alpha_{l'}}{V_g + 4 \sum_{l \in L} \sum_{\substack{l' \in L \\ l' \neq l}} D^*_{ll'} \alpha_l \alpha_{l'}}, \tag{S.54}$$

from which

$$4 \sum_{l \in L} \sum_{\substack{l' \in L \\ l' \neq l}} D^*_{ll'} \alpha_l \alpha_{l'} \approx \frac{\rho_G}{1 - \rho_G} V_g \quad \left(= \frac{h^2 \rho}{1 - h^2 \rho} V_g \right). \tag{S.55}$$

Since, in equilibrium, $D_{ll'} = \tilde{D}_{ll'}$, this expression can also be written

$$2 \sum_{l \in L} \sum_{\substack{l' \in L \\ l' \neq l}} \left(D^*_{ll'} + \tilde{D}^*_{ll'} \right) \alpha_l \alpha_{l'} \approx \frac{\rho_G}{1 - \rho_G} V_g. \tag{S.56}$$

Because the additive genetic variance $V_G = V_g + 2 \sum_{l \in L} \sum_{\substack{l' \in L \\ l' \neq l}} (D^*_{ll'} + \tilde{D}^*_{ll'}) \alpha_l \alpha_{l'}$, Eq. (S.56) can also be written

$$V_G = V_g / (1 - \rho_G), \tag{S.57}$$

which is a classic result [Ch. 4]wright1921, crow1970.

If we make the further assumption that effect sizes are the same across loci ($\alpha_l = \alpha$ for all $l \in L$), then Eq. (S.56) becomes

$$2 \sum_{l \in L} \sum_{\substack{l' \in L \\ l' \neq l}} \left(D^*_{ll'} + \tilde{D}^*_{ll'} \right) \approx \frac{1}{\alpha^2} \frac{\rho_G}{1 - \rho_G} V_g. \tag{S.58}$$

In a population association study at locus l, assuming no indirect effects and no sources of genetic confounding other than assortative mating, the effect-size estimate is

$$\hat{\alpha}_l = \alpha_l + \frac{2}{V_l} \sum_{\substack{l' \in L \\ l' \neq l}} \left(D^*_{ll'} + \tilde{D}^*_{ll'} \right) \alpha_{l'},$$

so that the proportionate bias in the effect-size estimate at l is

$$\frac{\hat{\alpha}_l - \alpha_l}{\alpha_l} = \frac{2}{V_l} \sum_{\substack{l' \in L \\ l' \neq l}} \left(D^*_{ll'} + \tilde{D}^*_{ll'} \right) \frac{\alpha_{l'}}{\alpha_l} = \frac{2}{H_l} \sum_{\substack{l' \in L \\ l' \neq l}} \left(D^*_{ll'} + \tilde{D}^*_{ll'} \right), \tag{S.59}$$

since $\alpha_{l'} = \alpha_l$ by assumption and $V_l \approx H_l = 2p_l(1 - p_l)$ because assortative mating does not substantially increase within-locus homozygosity when the number of loci that affect the trait is large [5, Ch. 4]. The

average proportionate bias across loci is then

$$
\begin{aligned}
\frac{1}{|L|}\sum_{l\in L}\frac{\hat{\alpha}_l-\alpha_l}{\alpha_l} &= \frac{1}{|L|}\sum_{l\in L}\frac{2}{H_l}\sum_{\substack{l'\in L\\ l'\neq l}}\left(D^*_{ll'}+\tilde{D}^*_{ll'}\right)\\
&\approx \frac{2}{|L|\bar{H}}\sum_{l\in L}\sum_{\substack{l'\in L\\ l'\neq l}}\left(D^*_{ll'}+\tilde{D}^*_{ll'}\right)\\
&\approx \frac{1}{|L|\bar{H}\alpha^2}\frac{\rho_G}{1-\rho_G}V_g\\
&\approx \frac{1}{V_g}\frac{\rho_G}{1-\rho_G}V_g\\
&= \frac{\rho_G}{1-\rho_G},
\end{aligned}
\tag{S.60}
$$

where we have used Eq. (S.58) and have assumed that minor allele frequencies do not differ widely across loci. Since $\rho_G = h^2\rho$, where ρ is the phenotypic correlation among mates and $h^2 = V_G/V_P$ is the heritability of the trait, Eq. (S.60) can also be written

$$
\frac{1}{|L|}\sum_{l\in L}\frac{\hat{\alpha}_l-\alpha_l}{\alpha_l} = \frac{h^2\rho}{1-h^2\rho}. \tag{S.61}
$$

Sex-symmetric cross-trait assortative mating with distinct genetic bases and equal effect sizes. In the case of cross-trait assortative mating, if the sets of loci underlying the two traits, L_1 and L_2, are distinct, then $\alpha_l \neq 0 \Rightarrow \beta_l = 0$ and $\beta_l \neq 0 \Rightarrow \alpha_l = 0$. In this case, Eq. (S.53) becomes

$$
\rho_G = \frac{4\sum\limits_{l\in L_1}\sum\limits_{l'\in L_2} D^*_{ll'}\alpha_l\beta_{l'}}{\sqrt{V^1_G V^2_G}}, \tag{S.62}
$$

from which

$$
\rho_G\sqrt{V^1_G V^2_G} = 4\sum_{l\in L_1}\sum_{l'\in L_2} D^*_{ll'}\alpha_l\beta_{l'} = 2\sum_{l\in L_1}\sum_{l'\in L_2}\left(D^*_{ll'}+\tilde{D}^*_{ll'}\right)\alpha_l\beta_{l'}. \tag{S.63}
$$

Because assortative mating is cross-trait, the LDs that assortative mating induces across L_1 and L_2 will dominate the second-order LDs induced within L_1 and within L_2. Therefore, $V^1_G \approx V^1_g$ and $V^2_G \approx V^2_g$.

The effect-size estimate at a locus $l \in L_1$ in a population GWAS on trait 2 is

$$
\hat{\beta}_l \approx \frac{2}{V_l}\sum_{l'\in L_2}\left(D_{ll'}+\tilde{D}_{ll'}\right)\beta_{l'} \approx \frac{2}{H_l}\sum_{l'\in L_2}\left(D_{ll'}+\tilde{D}_{ll'}\right)\beta_{l'}, \tag{S.64}
$$

while the true effect size β_l is zero, since $l \notin L_2$. In equilibrium, the average effect-size estimate, and thus the average deviation of these estimates from the true values, is therefore

$$
\frac{1}{|L_1|}\sum_{l\in L_1}\hat{\beta}_l \approx \frac{1}{|L_1|}\sum_{l\in L_1}\frac{2}{H_l}\sum_{l'\in L_2}\left(D_{ll'}+\tilde{D}_{ll'}\right)\beta_{l'} \approx \frac{2}{|L_1|\bar{H}_1}\sum_{l\in L_1}\sum_{l'\in L_2}\left(D_{ll'}+\tilde{D}_{ll'}\right)\beta_{l'}, \tag{S.65}
$$

where we have assumed that minor allele frequencies are not very different across L_1 ($\bar{H}_1$ is the average heterozygosity in L_1).

If we further assume that effect sizes at causal loci are equal for each trait ($\alpha_l = \alpha$ for all $l \in L_1$ and $\beta_{l'} = \beta$ for all $l' \in L_2$), then Eq. (S.65) can be written

$$\begin{aligned}
\frac{1}{|L_1|}\sum_{l\in L_1}\hat{\beta}_l &\approx \frac{2}{|L_1|\bar{H}_1}\sum_{l\in L_1}\sum_{l'\in L_2}\left(D_{ll'}+\tilde{D}_{ll'}\right)\beta \\
&= \frac{\alpha}{|L_1|\bar{H}_1\alpha^2}\times 2\sum_{l\in L_1}\sum_{l'\in L_2}\left(D_{ll'}+\tilde{D}_{ll'}\right)\alpha\beta \\
&= \frac{\alpha}{V_g^1}\times \rho_G\sqrt{V_G^1V_G^2} \qquad \text{[from Eq. S.63]} \\
&\approx \rho_G\sqrt{\frac{V_G^1}{V_G^2}}\alpha = \sqrt{\frac{V_G^1}{V_P^1}\cdot\frac{V_G^2}{V_P^2}}\rho\sqrt{\frac{V_G^1}{V_G^2}}\alpha = \rho\frac{V_G^1}{\sqrt{V_P^1V_P^2}}\alpha,
\end{aligned} \tag{S.66}$$

recalling from Eq. (S.45) that $\rho_G = \sqrt{h_1h_2}\rho$.

In the further special case where both the genetic and the phenotypic variances of the two traits are equal, then so are the heritabilities of the two traits. In this case, Eq. (S.66) simplifies to

$$\frac{1}{|L_1|}\sum_{l\in L_1}\hat{\beta}_l \approx \frac{V_G}{V_P}\rho\alpha = h^2\rho\alpha, \tag{S.67}$$

where h^2 is the common heritability of the two traits.

Sex-symmetric cross-trait assortative mating for traits with different genetic architectures. Eq. (S.67) reveals an interesting role for genetic architecture in the bias that cross-trait assortative mating can generate in population association studies performed at non-causal loci. Suppose, as we did in deriving Eq. (S.67), that the two traits on which assortative mating is based have the same genetic and phenotypic variances, V_G and V, and therefore also the same heritabilities, h^2. We shall make the further assumption that the traits have the same genic variance, V_g. Assume further that the sets of loci underlying traits 1 and 2, L_1 and L_2, have similar mean heterozygosities $\approx \bar{H}$. Normalize the effect size sizes at loci causal for trait 2 to $\beta = 1$, so that the traits' common genic variance is $V_g = |L_2|\bar{H}$.

Suppose that we now perform a population GWAS for trait 2. At loci that are causal for trait 2 ($l \in L_2$), we will estimate effect sizes accurately: $\hat{\beta}_l \approx 1$ (there will be a small positive second-order bias, of order ρ^2, since the locus $l \in L_2$ comes into positive LD with loci $l' \in L_1$, which in turn have come into positive LD with loci $l'' \in L_2$).

At loci that are causal for trait 1 ($l \in L_1$), and which therefore have no effect on trait 2, we will estimate effect sizes on average as given by Eq. (S.67): $\hat{\beta}_l = h^2\rho\alpha$.

How does the number of loci underlying variation in trait 1, $|L_1|$, affect this biased estimate of their effect on trait 2? For the genic variance of trait 1 to be the same as that of trait 2, $V_g = |L_1|\bar{H}\alpha^2 = |L_2|\bar{H}\beta^2 = |L_2|\bar{H}$), and so we must have $\alpha^2 = |L_2|/|L_1|$. Substituting this into the average effect-size estimate at non-causal loci, $\hat{\beta}_l = h^2\rho\sqrt{|L_2|/|L_1|}$.

So, the average effect-size estimate at causal loci $l \in L_2$ is $\hat{\beta}_l \approx 1$, while the average effect-size estimate at non-causal loci $l \in L_1$ is $\hat{\beta}_l = h^2\rho\sqrt{|L_2|/|L_1|}$. How do these two quantities compare? If the number of loci underlying the two traits is the same, $L_1 = L_2$, and effect-size estimates at non-causal loci are smaller than those at causal loci by a factor of about $h^2\rho$. However, if there are more loci underlying trait 2 than underlying trait 1—i.e., if trait 1 has a more concentrated genetic architecture $L_1 < L_2$—then

the effect-size estimates at non-causal loci will be closer to those at causal loci. Indeed, if trait 1 has a sufficiently concentrated architecture relative to trait 2, specifically, if $L_1 < h^4\rho^2 L_2$, then the effect-size estimates at non-causal loci will, on average, be larger in magnitude than effect-size estimates at causal loci.

More generally, the calculations above suggest that, in a more realistic scenario where effect sizes vary across loci, the trait-2 GWAS distribution of magnitudes of effect-size estimates at trait-1 loci (non-causal) will overlap more with the distribution of magnitudes of effect-size estimates at trait-2 loci (causal) if the genetic architecture of trait 1 is more concentrated (Fig. 4). This will lead to a greater number of trait 1 loci being identified as statistically significantly associated with trait 2 in the trait-2 GWAS.

S3.1.2 Cross-trait assortative mating that is asymmetric with respect to sex

We now consider the case where the strength of assortative mating between two traits, as measured by their correlation coefficient across mating pairs, is not equal in the female-male and male-female directions. This is clearest in the case of an active mate preference exhibited by one sex for some phenotype exhibited by the other sex.

To study this case, we make several simplifying assumptions. First, we assume that the genetic bases of variation in the two traits are distinct: $\alpha_l \neq 0 \Leftrightarrow \beta_l = 0$. Second we assume that there is only one active direction of assortative mating: female trait 1 and male trait 2. That is, conditional on the mother's breeding value for trait 1 and the father's breeding value for trait 2, there is no correlation between the mother's breeding value for trait 2 and the father's breeding value for trait 1:

$$\mathrm{Cov}\left(G_2^{\mathrm{m}}, G_1^{\mathrm{f}} \mid \{G_1^{\mathrm{m}}, G_2^{\mathrm{f}}\}\right) = 0.$$

Suppose that there is a constant correlation ρ_G between mothers' breeding values for trait 1 and fathers' breeding values for trait 2:

$$\rho_G = \frac{\mathrm{Cov}\left(G_1^{\mathrm{m}}, G_2^{\mathrm{f}}\right)}{\sqrt{V_G^1 V_G^2}}. \tag{S.68}$$

To study the genetic consequences of this assortment, we need to know the average bi-directional correlation among mates for traits 1 and 2 (Eq. S.44). Since traits 1 and 2 will come into a positive genetic correlation via assortative mating of female trait 1 and male trait 2, there will also be a positive covariance between mothers' breeding values for trait 2 and fathers' breeding values for trait 1, which we can express using the law of total covariance:

$$\begin{aligned}\mathrm{Cov}\left(G_2^{\mathrm{m}}, G_1^{\mathrm{f}}\right) &= \mathrm{Cov}_{\{G_1^{\mathrm{m}}, G_2^{\mathrm{f}}\}}\left(\mathbb{E}\left[G_2^{\mathrm{m}} \mid \{G_1^{\mathrm{m}}, G_2^{\mathrm{f}}\}\right], \mathbb{E}\left[G_1^{\mathrm{f}} \mid \{G_1^{\mathrm{m}}, G_2^{\mathrm{f}}\}\right]\right) \\ &\quad + \mathbb{E}_{\{G_1^{\mathrm{m}}, G_2^{\mathrm{f}}\}}\left[\mathrm{Cov}\left(G_2^{\mathrm{m}}, G_1^{\mathrm{f}} \mid \{G_1^{\mathrm{m}}, G_2^{\mathrm{f}}\}\right)\right] \\ &= \mathrm{Cov}_{\{G_1^{\mathrm{m}}, G_2^{\mathrm{f}}\}}\left(\mathbb{E}\left[G_2^{\mathrm{m}} \mid G_1^{\mathrm{m}}\right], \mathbb{E}\left[G_1^{\mathrm{f}} \mid G_2^{\mathrm{f}}\right]\right). \end{aligned} \tag{S.69}$$

If G_1^{m} and G_2^{m} are bivariate normal (more generally, if $G_2^{\mathrm{m}} = a + bG_1^{\mathrm{m}} + \varepsilon$, with $\mathbb{E}[\varepsilon] = \mathbb{E}[\varepsilon G_1^{\mathrm{m}}] = 0$), then

$$\begin{aligned}\mathbb{E}\left[G_2^{\mathrm{m}} \mid G_1^{\mathrm{m}}\right] &= \mathbb{E}[G_2^{\mathrm{m}}] + \rho_{\mathrm{m1,m2}}\sqrt{V_G^2/V_G^1}\,(G_1^{\mathrm{m}} - \mathbb{E}[G_1^{\mathrm{m}}]) \\ &= \mathbb{E}[G_2^{\mathrm{m}}] + \rho_{\mathrm{m1,m2}}\,(G_1^{\mathrm{m}} - \mathbb{E}[G_1^{\mathrm{m}}]),\end{aligned}$$

where $\rho_{\mathrm{m1,m2}} = \mathrm{Corr}\left(G_1^{\mathrm{m}}, G_2^{\mathrm{m}}\right)$ is the genetic correlation between traits 1 and 2 in mothers, and where we have assumed that the two traits have equal variance. Similarly, if G_1^{f} and G_2^{f} are bivariate normal, then

$$\mathbb{E}\left[G_1^{\mathrm{f}} \mid G_2^{\mathrm{f}}\right] = \mathbb{E}\left[G_1^{\mathrm{f}}\right] + \rho_{\mathrm{f1,f2}}\left(G_2^{\mathrm{f}} - \mathbb{E}\left[G_2^{\mathrm{f}}\right]\right).$$

Substituting these expressions into Eq. (S.69),

$$\mathrm{Cov}\left(G_2^{\mathrm{m}}, G_1^{\mathrm{f}}\right) = \rho_{\mathrm{m1,m2}}\,\rho_{\mathrm{f1,f2}}\,\mathrm{Cov}\left(G_1^{\mathrm{m}}, G_2^{\mathrm{f}}\right). \tag{S.70}$$

But, in our case, $\rho_{\mathrm{m1,m2}} = \rho_{\mathrm{f1,f2}}$, the common value of which we shall call ρ_{12}, and so the average bi-directional correlation is

$$\frac{\frac{1}{2}\left[\mathrm{Cov}\left(G_1^{\mathrm{m}}, G_2^{\mathrm{f}}\right) + \mathrm{Cov}\left(G_2^{\mathrm{m}}, G_1^{\mathrm{f}}\right)\right]}{\sqrt{V_G^1 V_G^2}} = \frac{\frac{1}{2}\left(1+\rho_{12}^2\right)\mathrm{Cov}\left(G_1^{\mathrm{m}}, G_2^{\mathrm{f}}\right)}{\sqrt{V_G^1 V_G^2}} = \frac{\rho_G}{2}\left(1+\rho_{12}^2\right). \tag{S.71}$$

Given this value, the calculations of the effect of assortative mating on the weighted sums of cis- and trans-covariances, and thus on the additive genetic variance, proceed as for the case of symmetric assortative mating above.

Assuming the genetic bases of the two traits to be distinct, we may substitute the average bi-directional correlation, $\rho_G\left(1+\rho_{12}^2\right)/2$, into Eq. (S.63) to find

$$\rho_G\left(1+\rho_{12}^2\right) = \frac{4\sum_{l\in L_1}\sum_{l'\in L_2}\left(D_{ll'} + \tilde{D}_{ll'}\right)\alpha_l\beta_{l'}}{\sqrt{V_G^1 V_G^2}}. \tag{S.72}$$

But

$$\rho_{12} = \frac{2\sum_{l\in L_1}\sum_{l'\in L_2}\left(D_{ll'} + \tilde{D}_{ll'}\right)\alpha_l\beta_{l'}}{\sqrt{V_G^1 V_G^2}},$$

and so Eq. (S.72) can be written as the quadratic equation $\rho_G(1+\rho_{12}^2) = 2\rho_{12}$, the relevant solution to which is $\rho_{12} = \left(1 - \sqrt{1-\rho_G^2}\right)/\rho_G$. If ρ_G is small, we use the first-order Taylor approximation $\sqrt{1-\rho_G^2} \approx 1 - \rho_G^2/2$ to find

$$\frac{\rho_G}{2} \approx \rho_{12} = \frac{2\sum_{l\in L_1}\sum_{l'\in L_2}\left(D_{ll'} + \tilde{D}_{ll'}\right)\alpha_l\beta_{l'}}{\sqrt{V_G^1 V_G^2}} \approx \frac{2\sum_{l\in L_1}\sum_{l'\in L_2}\left(D_{ll'} + \tilde{D}_{ll'}\right)\alpha_l\beta_{l'}}{\sqrt{V_g^1 V_g^2}}. \tag{S.73}$$

In the particular scenario we have simulated in Fig. 2, $V_g^1 = V_g^2$, $\alpha_l = 1$ for all $l \in L_1$, and $\beta_l = 1$ for all $l \in L_2$, so Eq. (S.73) further simplifies to

$$4\sum_{l\in L_1}\sum_{l'\in L_2}\left(D_{ll'} + \tilde{D}_{ll'}\right) = \rho V_g^1 \tag{S.74}$$

In a population association study for trait 2 performed at a locus $l \in L_1$ (so that $\beta_l = 0$),

$$\hat{\beta}_l = \beta_l + \frac{2}{V_l}\sum_{l'\in L_2}\left(D_{ll'} + \tilde{D}_{ll'}\right)\beta_{l'} = \frac{2}{V_l}\sum_{l'\in L_2}\left(D_{ll'} + \tilde{D}_{ll'}\right). \tag{S.75}$$

Across loci in L_1, the average estimate is

$$\overline{\hat{\beta}_l} = \frac{1}{|L_1|} \sum_{l \in L_1} \frac{2}{V_l} \sum_{l' \in L_2} \left(D_{ll'} + \tilde{D}_{ll'} \right). \tag{S.76}$$

In our simulations, $p_l \approx 1/2$ for all l so that $V_l \approx 2p_l(1-p_l) = 1/2$, and $|L_1| = |L_2| = 500$, so $V_g^1 = V_g^2 = 250$. Under this configuration,

$$\begin{aligned} \overline{\hat{\beta}_l} &= \frac{1}{|L_1|} \sum_{l \in L_1} \frac{2}{V_l} \sum_{l' \in L_2} \left(D_{ll'} + \tilde{D}_{ll'} \right) = \frac{1}{500} \sum_{l \in L_1} \frac{2}{1/2} \sum_{l' \in L_2} \left(D_{ll'} + \tilde{D}_{ll'} \right) \\ &= \frac{4}{500} \sum_{l \in L_1} \sum_{l' \in L_2} \left(D_{ll'} + \tilde{D}_{ll'} \right) = \frac{\rho_G V_g^1}{500} = \rho_G/2. \end{aligned}$$

The trait we simulated is genetic, with heritability 1, and so $\rho_G = \rho$, the phenotypic correlation among mates. We chose a strength of assortative mating of $\rho = 0.2$, and so, in equilibrium, the average effect-size estimate at non-causal loci should be approximately 0.1, which is indeed the case in Fig. 2.

Sex-asymmetric cross-trait assortative mating for traits with different genetic architectures. For the case where the numbers of loci underlying traits 1 and 2 differ, and noting that the 'effective' correlation among mates in the sex-asymmetric case is approximately half that in the sex-symmetric case (Eq. S.73), we can perform a similar back-of-the-envelope calculation as in the sex-symmetric cross-trait assortative mating case above to find that, when effect sizes are constant across trait-1 loci and constant across trait-2 loci (though differing across traits 1 and 2), the effect-size estimates at trait-1 (non-causal) loci in a trait-2 population GWAS is, on average, a fraction $\frac{h^2\rho}{2}\sqrt{|L_2|/|L_1|}$ of the estimates at trait-2 (causal) loci.

Thus, more generally, when the number of loci underlying trait 1 is small relative to the number of loci underlying trait 2, the distribution of magnitudes of effect-size estimates at trait-1 loci in a trait-2 GWAS can overlap substantially with the distribution of magnitudes of effect-size estimates at trait-2 loci (Fig. 4), causing variants at these non-causal trait-1 loci to show up as significant in the trait-2 GWAS.

S3.2 Population structure

In the model we have considered, with results displayed in Fig. 5, there are initially two isolated populations of equal size. The frequency of the focal variant at locus l is $p_l^{(1)}$ in population 1 and $p_l^{(2)}$ in population 2, so that its overall frequency is $p_l = \left(p_l^{(1)} + p_l^{(2)} \right)/2$. A population GWAS at locus λ returns an effect-size estimate

$$\hat{\alpha}_\lambda^{\text{pop}} = \frac{2}{V_\lambda} \sum_{l \in L} (D_{\lambda l} + \tilde{D}_{\lambda l}) \alpha_l,$$

where $D_{\lambda l}$ and $\tilde{D}_{\lambda l}$ are calculated across both populations and are generally nonzero because of allele frequency differences between the two populations at loci λ and l [6]. In our case,

$$V_\lambda = 2p_\lambda(1-p_\lambda)(1+F_\lambda),$$

and

$$D_{\lambda l} = \tilde{D}_{\lambda l} = \frac{1}{4} \left(p_\lambda^{(1)} - p_\lambda^{(2)} \right) \left(p_l^{(1)} - p_l^{(2)} \right),$$

so

$$\hat{\alpha}_\lambda^{\mathrm{pop}} = \frac{p_\lambda^{(1)} - p_\lambda^{(2)}}{2p_\lambda(1-p_\lambda)(1+F_\lambda)} \sum_{l \in L} \left(p_l^{(1)} - p_l^{(2)}\right) \alpha_l.$$

Squaring this and multiplying by $2p_\lambda(1-p_\lambda)$,

$$2p_\lambda(1-p_\lambda)(\hat{\alpha}_\lambda^{\mathrm{pop}})^2 = \frac{\left(p_\lambda^{(1)} - p_\lambda^{(2)}\right)^2}{2p_\lambda(1-p_\lambda)(1+F_\lambda)^2} \left[\sum_{l \in L} \left(p_l^{(1)} - p_l^{(2)}\right)^2 \alpha_l^2 + \sum_{l \neq l'} \left(p_l^{(1)} - p_l^{(2)}\right)\left(p_{l'}^{(1)} - p_{l'}^{(2)}\right) \alpha_l \alpha_{l'}\right]. \tag{S.77}$$

Neutral allele frequency divergence. If allele frequency divergence between the two populations is neutral, frequency changes at different loci are independent of one another and of effect sizes, so the second term in square brackets above is zero in expectation. In addition, because Hardy-Weinberg equilibrium obtains within each population, non-zero expected values of F_λ derive only from allele frequency differences between the populations, so that $F_\lambda = F_{ST,\lambda}$ in expectation. Therefore,

$$\begin{aligned}
\mathbb{E}\left[2p_\lambda(1-p_\lambda)(\hat{\alpha}_\lambda^{\mathrm{pop}})^2\right] &= \frac{1}{(1+F_{ST})^2}\mathbb{E}\left[\frac{\left(p_\lambda^{(1)} - p_\lambda^{(2)}\right)^2}{2p_\lambda(1-p_\lambda)}\right] |L|\, \mathbb{E}\left[\left(p_l^{(1)} - p_l^{(2)}\right)^2\right] \mathbb{E}\left[\alpha_l^2\right] \\
&= \frac{1}{(1+F_{ST})^2}\mathbb{E}\left[2F_{ST,\lambda}\right] |L|\, \mathbb{E}\left[2F_{ST,l}H_l\right] \mathbb{E}\left[\alpha_l^2\right] \\
&\approx \frac{4|L|}{(1+F_{ST})^2} \left(\mathbb{E}\left[F_{ST,l}\right]\right)^2 \mathbb{E}\left[H_l\right] \mathbb{E}\left[\alpha_l^2\right] \\
&= 4|L| \left(\frac{F_{ST}}{1+F_{ST}}\right)^2 \mathbb{E}\left[H_l\right] \mathbb{E}\left[\alpha_l^2\right],
\end{aligned}$$

where $H_l = 2p_l(1-p_l)$. If the ancestral allele frequency at l was p_l^{a}, then $\mathbb{E}[H_l | p_l^{\mathrm{a}}] = 2p_l^{\mathrm{a}}(1-p_l^{\mathrm{a}})(1-F_{ST,l})$, and so $\mathbb{E}[H_l]$ is calculated using the law of iterated expectations by averaging this quantity over the ancestral distribution of allele frequencies: $\mathbb{E}[H_l] \approx \mathbb{E}[H_l^{\mathrm{a}}](1-F_{ST})$, where $H_l^{\mathrm{a}} = 2p_l^{\mathrm{a}}(1-p_l^{\mathrm{a}})$. So

$$\mathbb{E}\left[2p_\lambda(1-p_\lambda)(\hat{\alpha}_\lambda^{\mathrm{pop}})^2\right] \approx 4|L| \left(\frac{F_{ST}}{1+F_{ST}}\right)^2 (1-F_{ST})\mathbb{E}\left[H_l^{\mathrm{a}}\right] \mathbb{E}\left[\alpha_l^2\right]. \tag{S.78}$$

Selection and phenotype-biased migration. Above, in calculating the mean heterozygosity-weighted value of $(\hat{\alpha}_\lambda)^2$ under neutral frequency divergence between populations, we assumed that in Eq. (S.77) the second term in the square brackets was zero, i.e., that the effect-size-signed population allele frequency difference was uncorrelated across loci. Howevever, when selection or phenotype-biased migration acts, this will no longer be true. For example, if higher genetic values of the trait were favoured in population 1 relative to population 2, then selection will on average have driven a mean shift such that $\mathbb{E}\left[\left(p_l^{(1)} - p_l^{(2)}\right)\alpha_l\right] > 0$. This in turn will drive systematic positive covariances between terms $\left(p_l^{(1)} - p_l^{(2)}\right)\alpha_l$ and $\left(p_{l'}^{(1)} - p_{l'}^{(2)}\right)\alpha_{l'}$, and as these covariances are summed over all pairs of loci in Eq. (S.77), the resulting inflation of the average squared effect-size estimate (and other genome-wide summaries) could be quantitatively substantial.

More general population stratification. Given a sample of N individuals, the sample cis-LD between two markers λ and l can be written generally as

$$D_{\lambda l} = \frac{1}{N-1} \sum_{i=1}^{N} \left(\Delta g_{i,\lambda}^{\mathrm{m}} \Delta g_{i,l}^{\mathrm{m}} + \Delta g_{i,\lambda}^{\mathrm{p}} \Delta g_{i,l}^{\mathrm{p}} \right), \tag{S.79}$$

where $\Delta g_{i,k}^{\mathrm{m}}$ and $\Delta g_{i,k}^{\mathrm{p}}$ are the deviations of individual i's maternal and paternal focal allele count at locus k from their mean frequencies. The trans-LD between λ and l is

$$\tilde{D}_{\lambda l} = \frac{1}{N-1} \sum_{i=1}^{N} \left(\Delta g_{i,\lambda}^{\mathrm{m}} \Delta g_{i,l}^{\mathrm{p}} + \Delta g_{i,l}^{\mathrm{m}} \Delta g_{i,\lambda}^{\mathrm{p}} \right). \tag{S.80}$$

These cis- and trans-LD terms are equal only if

$$D_{\lambda l} - \tilde{D}_{\lambda l} = \frac{1}{N-1} \sum_{i=1}^{N} \left(\Delta g_{i,\lambda}^{\mathrm{m}} - \Delta g_{i,\lambda}^{\mathrm{p}} \right) \left(\Delta g_{i,l}^{\mathrm{m}} - \Delta g_{i,l}^{\mathrm{p}} \right) = 0, \tag{S.81}$$

i.e., if the maternal and paternal alleles at the one locus are exchangeable with respect to deviations of the allelic state at the other locus.

We might often be concerned with stratification along some specific axis of variation in our sample. Call this axis v, with every individual having a value along v, with mean zero across individuals (for example, in our two population case above, the vector v could be 1 for population 1 and -1 for population 2). The covariance of the maternal allele at locus l with the vector v is proportional to $a_l^{\mathrm{m}} \cdot v = \sum_i a_{i,l}^{\mathrm{m}} v_i$. So the contribution of LD along this axis to the difference in cis- and trans-LD is

$$D_{\lambda l}^{(v)} - \tilde{D}_{\lambda l}^{(v)} = \left(\left(\Delta g_{\lambda}^{\mathrm{m}} - \Delta g_{\lambda}^{\mathrm{p}} \right) \cdot v \right) \left(\left(\Delta g_{l}^{\mathrm{m}} - \Delta g_{l}^{\mathrm{p}} \right) \cdot v \right), \tag{S.82}$$

which is zero only if the maternal and paternal genotypes at the two loci are exchangeable with respect to each other along the axis v.

S3.3 Admixture

Suppose that two previously isolated populations admix in proportions A and $1-A$, with subsequent random mating in the admixed population. Following the notation in the Section S3.2 above, before admixture, the frequency of the focal variant at locus l was $p_l^{(1)}$ in population 1 and $p_l^{(2)}$ in population 2, so that its overall frequency in the admixed population is $p_l = A p_l^{(1)} + (1-A) p_l^{(2)}$.

When the two populations admix, trans-LD between all pairs of loci disappears in expectation, owing to random mating in the admixed population: $\tilde{D}_{\lambda l}^{t} = 0$ for any pairs of loci λ and l and for any number of generations t after admixture. However, cis-associations between alleles that were more prevalent in one ancestral population than in the other will be retained as cis-LD in the admixed population until these associations are eroded by recombination. The initial degree of cis-LD between loci λ and l in the admixed population is

$$D_{\lambda l}^{0} = A(1-A) \left(p_{\lambda}^{(1)} - p_{\lambda}^{(2)} \right) \left(p_{l}^{(1)} - p_{l}^{(2)} \right).$$

When t generations have elapsed since admixture, this cis-LD will have been eroded by recombination to

$$D_{\lambda l}^{t} = D_{\lambda l}^{0} (1 - c_{\lambda l})^{t} = A(1-A) \left(p_{\lambda}^{(1)} - p_{\lambda}^{(2)} \right) \left(p_{l}^{(1)} - p_{l}^{(2)} \right) (1 - c_{\lambda l})^{t},$$

where $c_{\lambda l}$ is the sex-averaged recombination rate between λ and l. Therefore, t generations after admixture, a population association study at λ returns an effect-size estimate

$$\hat{\alpha}_{\lambda}^{\text{pop},t} = \frac{2}{V_\lambda} \sum_{l \in L} D_{\lambda l}^{t} \alpha_l = A(1-A) \frac{p_\lambda^{(1)} - p_\lambda^{(2)}}{p_\lambda (1-p_\lambda)} \sum_{l \in L} \left(p_l^{(1)} - p_l^{(2)} \right) (1 - c_{\lambda l})^t \alpha_l,$$

while a sibling-based association study at λ returns

$$\hat{\alpha}_{\lambda}^{\text{sib},t} = \frac{2}{H_\lambda} \sum_{l \in L} (1 - 2c_{\lambda l}) D_{\lambda l}^{t} \alpha_l = A(1-A) \frac{p_\lambda^{(1)} - p_\lambda^{(2)}}{p_\lambda (1-p_\lambda)} \sum_{l \in L} \left(p_l^{(1)} - p_l^{(2)} \right) (1 - c_{\lambda l})^t (1 - 2c_{\lambda l}) \alpha_l,$$

where we have substituted $V_\lambda = H_\lambda = 2p_\lambda(1-p_\lambda)$ owing to random mating in the admixed population.

Squaring the population estimate and multiplying by $2p_\lambda(1-p_\lambda)$,

$$2p_\lambda(1-p_\lambda)(\hat{\alpha}_{\lambda}^{\text{pop},t})^2 = 2A^2(1-A)^2 \frac{\left(p_\lambda^{(1)} - p_\lambda^{(2)} \right)^2}{p_\lambda (1-p_\lambda)} \left[\sum_{l \in L} \left(p_l^{(1)} - p_l^{(2)} \right)^2 (1 - c_{\lambda l})^{2t} \alpha_l^2 \right.$$
$$\left. + \sum_{l \neq l'} \left(p_l^{(1)} - p_l^{(2)} \right) \left(p_{l'}^{(1)} - p_{l'}^{(2)} \right) (1 - c_{\lambda l})^t (1 - c_{\lambda l'})^t \alpha_l \alpha_{l'} \right], \quad \text{(S.83)}$$

while the heterozygosity-weighted squared sibling effect size is

$$2p_\lambda(1-p_\lambda)(\hat{\alpha}_{\lambda}^{\text{sib},t})^2 = 2A^2(1-A)^2 \frac{\left(p_\lambda^{(1)} - p_\lambda^{(2)} \right)^2}{p_\lambda (1-p_\lambda)} \left[\sum_{l \in L} \left(p_l^{(1)} - p_l^{(2)} \right)^2 (1 - c_{\lambda l})^{2t} (1 - 2c_{\lambda l})^2 \alpha_l^2 \right.$$
$$\left. + \sum_{l \neq l'} \left(p_l^{(1)} - p_l^{(2)} \right) \left(p_{l'}^{(1)} - p_{l'}^{(2)} \right) (1 - c_{\lambda l})^t (1 - c_{\lambda l'})^t (1 - 2c_{\lambda l})(1 - 2c_{\lambda l'}) \alpha_l \alpha_{l'} \right]. \quad \text{(S.84)}$$

Neutral allele frequency divergence. If allele frequency divergence between the two populations was neutral, then frequency changes at different loci are independent of one another, of effect sizes, and of recombination rates (assuming the loci are sufficiently far apart), so the second terms in square brackets in Eqs. (S.83) above is zero in expectation, so that

$$\begin{aligned}
\mathbb{E}\left[2p_\lambda(1-p_\lambda)(\hat{\alpha}_{\lambda}^{\text{pop},t})^2 \right] &= 4A^2(1-A)^2 \mathbb{E}\left[\frac{\left(p_\lambda^{(1)} - p_\lambda^{(2)} \right)^2}{2p_\lambda(1-p_\lambda)} \right] |L| \, \mathbb{E}\left[\left(p_l^{(1)} - p_l^{(2)} \right)^2 \right] \overline{(1-c)^{2t}} \, \mathbb{E}\left[\alpha_l^2 \right] \\
&= 4A^2(1-A)^2 \overline{(1-c)^{2t}} \mathbb{E}\left[2F_{ST,\lambda} \right] |L| \, \mathbb{E}\left[2F_{ST,l} H_l \right] \mathbb{E}\left[\alpha_l^2 \right] \\
&\approx 16A^2(1-A)^2 \overline{(1-c)^{2t}} \, |L| F_{ST}^2 \mathbb{E}\left[H_l \right] \mathbb{E}\left[\alpha_l^2 \right],
\end{aligned}$$

where $\overline{(1-c)^{2t}}$ is the average value of $(1-c_{ll'})^{2t}$ taken across all pairs of loci l, l'.

Similarly, under drift in the ancestral populations, the average squared sibling-based effect-size estimate can be simplified to

$$\mathbb{E}\left[2p_\lambda(1-p_\lambda)(\hat{\alpha}_{\lambda}^{\text{sib},t})^2 \right] \approx 16A^2(1-A)^2 \overline{(1-c)^{2t}(1-2c)^2} \, |L| F_{ST}^2 \mathbb{E}\left[H_l \right] \mathbb{E}\left[\alpha_l^2 \right],$$

where $\overline{(1-c)^{2t}(1-2c)^2}$ is the average value of $(1-c_{ll'})^{2t}(1-2c_{ll'})$ taken across all pairs of loci l, l'.

Selection and phenotype-biased migration. As in the case of population structure, selection and phenotype-biased migration in the ancestral populations can drive systematic positive covariances between the terms $\left(p_l^{(1)} - p_l^{(2)}\right) \alpha_l$ and $\left(p_{l'}^{(1)} - p_{l'}^{(2)}\right) \alpha_{l'}$ in Eqs. (S.83) and (S.84) above, so that the second terms in square brackets in these equations do not cancel in expectation as they did under neutral divergence between the ancestral populations. Again, as these covariances are summed over all pairs of loci in Eqs. (S.83) and (S.84), the resulting inflation of the average squared effect-size estimate and other genome-wide summaries could be substantial.

S3.4 Stabilizing selection

We consider the model of Bulmer [7,8], in which a very large number of loci contribute variation to a trait under stabilizing selection. We assume that the distribution of trait values is centered on the optimal value Y^*, and that the relative fitness of an individual with trait value Y is $\exp\left(-(Y - Y^*)^2/2V_S\right)$, where V_S, the width or 'variance' of this gaussian selection function, governs the strength of stabilizing selection, with larger V_S values implying weaker selection.

Under this model, selection acts to reduce the phenotypic variation each generation. If the trait value is normally distributed with variance V_P, then selection reduces the within-generation phenotypic variance by an amount

$$\Delta V_P = \frac{-V_P^2}{V_S + V_P}. \tag{S.85}$$

If the trait is heritable, then this reduction in trait variance is partially reflected in a reduction in genetic variance for the trait, which takes the form of negative cis- and trans-LD between trait-increasing alleles. The reduction in trait variance (S.85) will then partially carry over to the offspring generation in the form of cis-LD; how much of it will carry over depends on the recombination process and the heritability of the trait.

Because selection generates both cis- and trans-LD within each generation, but only cis-LD is (partially) transmitted to the next generation, the degree to which stabilizing selection will confound a GWAS depends on whether selection has or has not yet acted in the generation from which the GWAS sample is drawn. Because the degree of LD generated by stabilizing selection is usually discussed in the theoretical literature in terms of its value among zygotes—i.e., before the action of selection—we shall initially focus on the case where the GWAS is performed before selection has acted in the sampled individuals' generation. We shall then turn to the case where selection has acted fully in the sampled individuals' generation. If stabilizing selection on the trait in question extends across individuals' lifetimes, such that it has only partially acted in the sampled individuals' generation, then its effects on the GWAS will lie between the pre-selection and post-selection extremes that we consider.

S3.4.1 GWASs performed before selection has acted in the sampled individuals' generation

Owing to the large number of loci in this model, the buildup of LD among them occurs on a faster timescale than the change in allele frequencies at individual loci. Assuming the loci to have equal effect sizes, Bulmer [8] showed that the overall reduction in the phenotypic variance due to stabilizing selection, d, measured before the action of stabilizing selection, rapidly approaches a quasi-equilibrium value that approximately satisfies

$$d^* = \frac{1}{2} h^{*4} \Delta V_P^* / \bar{c}_h, \tag{S.86}$$

where ΔV_P^* is the within-generation reduction in phenotypic variance in this equilibrium, h^{*2} is the equilibrium heritability of the trait, and $\bar{c}_h$ is the harmonic mean of the recombination rates amongst all pairs of loci. On this rapid timescale, the reduction in variance is due to cis-LD among the loci underlying the trait; in fact,

$$d = 2\alpha^2 \sum_{l \in L} \sum_{l' \in L} D_{ll'}, \tag{S.87}$$

where α is the common per-locus effect size and $D_{ll'}$ is defined with respect to the trait-increasing alleles at l and l'. The individual linkage disequilibria $D_{ll'}$, in expectation, are proportional to the inverse recombination rates $1/c_{ll'}$. Writing

$$2\alpha^2 \sum_{l \in L} \sum_{l' \in L} D_{ll'}^* = d^* = \frac{1}{2} h^{*4} \Delta V_P^* / \bar{c}_h = \frac{1}{2} h^{*4} \Delta V_P^* \frac{\sum_l \sum_{l' \neq l} 1/c_{ll'}}{\binom{|L|}{2}}, \tag{S.88}$$

where $\binom{|L|}{2} = |L|\big(|L|-1\big)/2$ is the number of pairs of distinct loci in L, it is apparent that

$$\mathbb{E}\left[D_{ll'}^*\right] = \frac{1}{4\alpha^2} h^{*4} \Delta V_P^* \frac{1/c_{ll'}}{\binom{|L|}{2}}. \tag{S.89}$$

Henceforth we deal only with equilibrium quantities and therefore drop the star superscript for neatness. The phenotypic variance V_P can be written $V_P = V_G + V_E = V_g + d + V_E$, where V_G is the additive genetic variance, V_g is the genic variance, and V_E is the variance due to the environment. Eqs. (S.85) and (S.86), together with the definition of heritability $h^2 = V_G/V_P$, define a quadratic equation in d:

$$(1 + 2\bar{c}_h)d^2 + 2[(V_S + V_g + V_E)\bar{c}_h + V_g]d + V_g^2 = 0. \tag{S.90}$$

Eq. (S.90) matches Eq. (10) in [8], with Bulmer's parameters c replaced by $1/2V_S$ and H by $\bar{c}_h$. For ease of reference in what follows, we write Eq. (S.90) in the standard form $ad^2 + bd + c = 0$. The roots are

$$d_{+,-} = \frac{-b \pm \sqrt{b^2 - 4ac}}{2a} = \frac{-[(V_S + V_g + V_E)\bar{c}_h + V_g] \pm \sqrt{[(V_S + V_g + V_E)\bar{c}_h + V_g]^2 - (1 + 2H)V_g^2}}{1 + 2\bar{c}_h}. \tag{S.91}$$

To see which of these roots is the relevant one, we first note that the roots are both real, since the requirement for this is

$$\begin{aligned} [(V_S + V_g + V_E)\bar{c}_h + V_g]^2 \geq (1 + 2\bar{c}_h)V_g^2 \quad &\Leftrightarrow \quad (V_S + V_g + V_E)\bar{c}_h + V_g \geq \sqrt{1 + 2\bar{c}_h}V_g \\ &\Leftrightarrow \quad V_S + V_E \geq \frac{\sqrt{1 + 2\bar{c}_h} - 1 - \bar{c}_h}{\bar{c}_h} V_g, \end{aligned}$$

and $\sqrt{1 + 2\bar{c}_h} < \sqrt{1 + 2\bar{c}_h + \bar{c}_h^2} = 1 + \bar{c}_h$ for $\bar{c}_h > 0$, while $V_S + V_E > 0$. Furthermore, since $b > 0$ and

This is a 'quasi-equilibrium' value because, when the number of loci underlying variation in the trait is finite, stabilizing selection induces underdominant selection at each locus, so that the frequency of the rarer allele is expected to decline. This reduces heterozygosity at individual loci, reducing variance in the trait. However, this reduction occurs on a slower timescale than the initial reduction due to the generation of linkage disequilibria.

$4ac > 0$, both roots are in fact negative, with $d_- < d_+ < 0$. Now note that

$$\begin{aligned} 2d_- < d_+ + d_- = -\frac{b}{a} &= -\frac{2[(V_S+V_g+V_E)\bar{c}_h + V_g]}{1+2\bar{c}_h} \\ &< -\frac{2[(V_g+V_g+V_E)\bar{c}_h + V_g]}{1+2\bar{c}_h} \qquad \text{(since } V_g < V_S\text{)} \\ &< -\frac{2[(V_g+V_g)\bar{c}_h + V_g]}{1+2\bar{c}_h} \qquad \text{(since } V_E > 0\text{)} \\ &= -2V_g, \end{aligned}$$

i.e., $V_g + d_- < 0$. But then if the relevant root were $d = d_-$, $0 \le V_G = V_g + d_- < 0$, a contradiction. So the relevant root is in fact

$$d = d_+ = \frac{-[(V_S+V_g+V_E)\bar{c}_h + V_g] + \sqrt{[(V_S+V_g+V_E)\bar{c}_h + V_g]^2 - (1+2\bar{c}_h)V_g^2}}{1+2\bar{c}_h}, \tag{S.92}$$

from which

$$-\frac{d}{V_g} = \frac{1 - \bar{c}_h\left(\sqrt{1+2\left(1+\frac{1}{\bar{c}_h}\right)X + X^2} - (1+X)\right)}{1+2\bar{c}_h}, \tag{S.93}$$

where $X = \frac{V_S+V_E}{V_g}$. Since, in the absence of selection, $V_G = V_g$, Eq. (S.93) gives the proportionate reduction in the additive genetic variance due to selection.

Note that

$$\begin{aligned} 1-\bar{c}_h\left(\sqrt{1+2\left(1+\frac{1}{\bar{c}_h}\right)X+X^2}-(1+X)\right) &< 1-\bar{c}_h\left(\sqrt{1+\frac{1}{\bar{c}_h}+2\left(1+\frac{1}{\bar{c}_h}\right)X+X^2}-(1+X)\right) \\ &= 1-\bar{c}_h\left(\sqrt{\left(1+\frac{1}{\bar{c}_h}+X\right)^2}-(1+X)\right) \end{aligned}$$

From Eq. (S.87), $d = 2\alpha^2\sum_l\sum_{l'\neq l} D_{ll'}$, and, since $V_g = \sum_l 2p_l(1-p_l)\alpha^2 = \alpha^2\bar{H}|L|$, with $|L|$ the number of loci and $\bar{H}$ the average heterozygosity across them, we have

$$\frac{d}{V_g} = \frac{2\sum_l\sum_{l'\neq l} D_{ll'}}{\bar{H}L}. \tag{S.94}$$

In a population association study performed at locus l, the effect-size estimate is

$$\hat{\alpha}_l^{\text{pop}} = \alpha_l + \frac{2}{2p_l(1-p_l)}\sum_{l'\neq l} D_{ll'}\alpha_{l'} = \alpha\left(1 + \frac{2}{2p_l(1-p_l)}\sum_{l'\neq l} D_{ll'}\right), \tag{S.95}$$

so that the proportionate error is

$$\frac{2}{2p_l(1-p_l)}\sum_{l'\neq l} D_{ll'}. \tag{S.96}$$

The mean proportionate error across loci is therefore

$$\frac{1}{|L|}\sum_{l\in L}\left(\frac{2}{2p_l(1-p_l)}\sum_{l'\neq l}D_{ll'}\right)\approx\frac{2\sum_l\sum_{l'\neq l}D_{ll'}}{\bar{H}|L|}=\frac{d}{V_g}, \tag{S.97}$$

from Eq. (S.94), and assuming that the heterozygosities do not vary much across loci. That is, the average proportionate bias to effect-size estimation that stabilizing selection induces is approximately equal to the proportionate reduction in the additive genetic variance, which is given in general form by Eq. (S.93).

In a within-family association study performed at locus l, the effect-size estimate is

$$\hat{\alpha}_l^{\text{fam}}=\alpha_l+\frac{2}{2p_l(1-p_l)}\sum_{l'\neq l}(1-2c_{ll'})D_{ll'}\alpha_{l'}=\alpha\left(1+\frac{2}{2p_l(1-p_l)}\sum_{l'\neq l}(1-2c_{ll'})D_{ll'}\right), \tag{S.98}$$

so that the proportionate error is

$$\frac{2}{2p_l(1-p_l)}\sum_{l'\neq l}(1-2c_{ll'})D_{ll'}. \tag{S.99}$$

The mean proportionate error across loci is therefore

$$\begin{aligned}\frac{1}{|L|}\sum_{l\in L}\left(\frac{2}{2p_l(1-p_l)}\sum_{l'\neq l}(1-2c_{ll'})D_{ll'}\right)&\approx\frac{2\sum_l\sum_{l'\neq l}(1-2c_{ll'})D_{ll'}}{\bar{H}|L|}\\&\approx\frac{2\sum_l\sum_{l'\neq l}(1-2c_{ll'})\frac{d\bar{c}_h}{2\alpha^2\binom{|L|}{2}c_{ll'}}}{\bar{H}|L|}\\&=\frac{d\bar{c}_h}{\alpha^2\bar{H}|L|\binom{|L|}{2}}\sum_l\sum_{l'}\left(\frac{1}{c_{ll'}}-2\right)\\&=\frac{d\bar{c}_h}{V_g\binom{|L|}{2}}\left(\frac{\binom{|L|}{2}}{\bar{c}_h}-2\binom{|L|}{2}\right)\\&=\frac{d}{V_g}\left(1-2\bar{c}_h\right),\end{aligned} \tag{S.100}$$

where we have used Eq. (S.89) in the second line. Therefore, the mean error in the within-family GWAS is smaller in magnitude than that in a population GWAS by a factor $1-2\bar{c}_h$.

S3.4.2 GWASs performed after selection has acted in the sampled individuals' generation

The calculations above assume that the GWASs are performed before selection has yet acted in the generation from which the GWAS sample is drawn. The genetic confounds induced by stabilizing selection are therefore due only to the cis-LD transmitted to this generation by the previous generation.

Incrementing the cis-LD transmitted to it by the previous generation, selection within a generation generates cis- and trans-LD in equal measure. Consider a pair of loci l and l', between which the equilibrium degree of cis-LD measured in zygotes (pre-selection) is $D_{ll'}^{\text{pre}}$. Selection within the generation adds an amount $x_{ll'}$ to this cis-LD and generates an equal amount $x_{ll'}$ of trans-LD between the loci. The amount

of cis-LD transmitted to the next generation is therefore $(D_{ll'}^{\text{pre}}+x_{ll'})(1-c_{ll'})+x_{ll'}c_{ll'} = (1-c_{ll'})D_{ll'}+x_{ll'}$. In equilibrium, this must equal $D_{ll'}^{\text{pre}}$, and so $x_{ll'} = c_{ll'}D_{ll'}$.

Notice that, because the cis- and trans-LD generated within each generation are equal in expectation, these within-generation incremental LDs will not affect the estimates produced by a within-family GWAS, which depend only on the difference between cis- and trans-LD (and therefore depend only on the cis-LD transmitted from the previous generation, as calculated above). However, they will affect a population-based GWAS, which depends on the sum of the cis- and trans-LDs. The effect-size estimate in a population GWAS performed at locus l is

$$\begin{aligned}\hat{\alpha}_l^{\text{pop}} &= \alpha\left(1+\frac{2}{2p_l(1-p_l)}\sum_{l'\neq l}\left(D_{ll'}+\tilde{D}_{ll'}\right)\right)\\ &= \alpha\left(1+\frac{2}{2p_l(1-p_l)}\sum_{l'\neq l}\left([D_{ll'}^{\text{pre}}+x_{ll'}]+x_{ll'}\right)\right)\\ &= \alpha\left(1+\frac{2}{2p_l(1-p_l)}\sum_{l'\neq l}(1+2c_{ll'})D_{ll'}^{\text{pre}}\right). \end{aligned} \tag{S.101}$$

But, from Eq. (S.89), $\mathbb{E}[D_{ll'}^{\text{pre}}] \propto 1/c_{ll'}$, and so

$$\sum_{l'\neq l} c_{ll'}D_{ll'}^{\text{pre}} = \bar{c}_h \sum_{l'\neq l} D_{ll'}^{\text{pre}},$$

from which it follows that

$$\hat{\alpha}_l^{\text{pop}} = \alpha\left(1+\frac{2}{2p_l(1-p_l)}\sum_{l'\neq l}(1+2c_{ll'})D_{ll'}^{\text{pre}}\right) = \alpha\left(1+\frac{2(1+2\bar{c}_h)}{2p_l(1-p_l)}\sum_{l'\neq l}D_{ll'}^{\text{pre}}\right). \tag{S.102}$$

The proportionate attentuation bias in the population GWAS performed after selection has acted is therefore

$$\frac{2(1+2\bar{c}_h)}{2p_l(1-p_l)}\sum_{l'\neq l}D_{ll'}^{\text{pre}}. \tag{S.103}$$

From Eq. (S.97), the mean proportionate error is therefore

$$\frac{(1+2\bar{c}_h)d}{V_g}; \tag{S.104}$$

that is, $1+2\bar{c}_h$ times greater than the proportionate attenuation bias in the population GWAS performed before selection has acted.

S3.4.3 Numerical values for humans

If ∼1,000 loci underlie variation in the trait (and all contribute approximately the same variation), $\bar{c}_h \approx 0.4640$ in humans (see Methods), and so the average bias that stabilizing selection induces in within-family GWASs will be about $1-2\bar{c}_h \approx 7.2\%$ that in pre-selection population GWASs and $(1-2\bar{c}_h)(1+2\bar{c}_h) \approx 3.7\%$ that in post-selection population GWASs. If ∼10,000 loci underlie variation in the trait, $\bar{c}_h \approx 0.4346$, and

so the bias in within-family GWASs will be about 13% that in pre-selection population GWASs and 7% that in post-selection population GWASs.

The calculations above give the average proportionate bias to GWAS estimates in terms of the basic parameters of the model, V_g, V_E, V_S, and $\bar{c}_h$. Often, however, not all of these parameters will be measurable. For example, human height appears to be under stabilizing selection [9], is highly heritable, and this heritability is believed to be underlain largely by *direct* genetic effects [10]. However, it is difficult to directly measure the genic variance in height V_g because not all causal loci will be assayed in association studies—and, moreover, even if they were, effect-size estimation at these causal loci would be biased by the genetic confounds that we have studied in this paper. However, the phenotypic variance in height V_P can obviously be measured, and the heritability of height h^2 can also be measured using classical methods rather than effect-size estimation in association studies. The strength of stabilizing selection on height can also be measured [9]. From V_P and h^2, the additive genetic variance V_G can be estimated ($V_G = h^2 V_P$).

This example suggests that, in many applications, it might be useful to be able to estimate the equilibrium value of d using V_G (or V_P), V_E, V_S, and $\bar{c}_h$, even though V_G (and V_P), in the model we have considered, is a state variable influenced by the state variable of primary interest, d. This is straightforward: returing to our use of a star superscript to denote equilibrium values, if we treat V_G and V_P as their equilibrium values V_G^* and V_P^*, Eq. (S.86) can be estimated directly, and also simplifies to

$$d^* = -\frac{1}{2\bar{c}_h} \cdot \frac{V_G^{*2}}{V_S + V_G^* + V_E} = -\frac{1}{2\bar{c}_h} \cdot \frac{V_G^{*2}}{V_S + V_P^*} = -\frac{1}{2\bar{c}_h} \cdot \frac{h^{*4} V_P^{*2}}{V_S + V_P^*}. \tag{S.105}$$

The proportionate bias in a pre-selection population GWAS, given by Eq. (S.97), can similarly be estimated from h^2, V_P, V_S, and $\bar{c}_h$, by first observing that

$$V_g = V_G^* - d^* = V_G^* + \frac{1}{2\bar{c}_h} \frac{V_G^{*2}}{V_S + V_P^*} = V_G^* \left(1 + \frac{1}{2\bar{c}_h} \cdot \frac{V_G^*}{V_S + V_P^*}\right),$$

so that Eq. (S.97) can be written

$$\frac{d^*}{V_g} = \frac{-\frac{1}{2\bar{c}_h} \cdot \frac{V_G^{*2}}{V_S + V_P^*}}{V_G^* \left(1 + \frac{1}{2\bar{c}_h} \cdot \frac{V_G^*}{V_S + V_P^*}\right)} = \frac{-\frac{1}{2\bar{c}_h} \cdot \frac{V_G^*}{V_S + V_P^*}}{1 + \frac{1}{2\bar{c}_h} \cdot \frac{V_G^*}{V_S + V_P^*}} = \frac{-\frac{1}{2\bar{c}_h} \cdot \frac{h^{*2} V_P^*}{V_S + V_P^*}}{1 + \frac{1}{2\bar{c}_h} \cdot \frac{h^{*2} V^*}{V_S + V_P^*}} = -\frac{1}{2\bar{c}_h \left(\frac{1 + V_S/V_P^*}{h^{*2}}\right) + 1}, \tag{S.106}$$

which reveals that the proportionate bias depends only on $\bar{c}_h$, h^{*2} and the scaled inverse strength of selection, V_S/V_P^*.

Similarly, the proportionate bias in a post-selection population GWAS, given above by Eq. (S.104), can also be written

$$\frac{d^*}{V_g}(1 + 2\bar{c}_h) = -\frac{1 + 2\bar{c}_h}{2\bar{c}_h \left(\frac{1 + V_S/V_P^*}{h^{*2}}\right) + 1}, \tag{S.107}$$

From Eq. (S.100), the proportionate bias in a within-family GWAS is approximately

$$\frac{d^*}{V_g}(1 - 2\bar{c}_h) = -\frac{1 - 2\bar{c}_h}{2\bar{c}_h \left(\frac{1 + V_S/V_P^*}{h^{*2}}\right) + 1}. \tag{S.108}$$

S3.4.4 Stabilizing selection attenuates estimates of the strength of assortative mating based on cross-chromosome PGS correlations

Recently, the strength of assortative mating has been estimated based on measurement of the correlation of polygenic scores across distinct sets of chromosomes (e.g., [11, 12]). Were assortative mating acting in isolation, such correlations would be due entirely to the positive cis- and trans-LDs among same-effect alleles created by assortative mating. Since stabilizing selection, acting in isolation, generates negative cis-LDs among same-effect alleles, it will attenuate the positive cis-LDs generated by assortative mating, and therefore reduce the correlation in PGSs among distinct sets of chromosomes, leading to underestimates of the strength of assortative mating if this effect is not taken into account.

To quantify this attenuation, we first calculate the strength of (positive) cross-chromosome LDs expected under assortative mating alone; then we calculate the strength of (negative) cross-chromosome LDs expected under stabilizing selection alone; then, assuming these LDs to be generated independently of one another—so that the LDs generated under the joint action of assortative mating and stabilizing selection are the sums of the LDs expected under these forces alone—we calculate how much stabilizing selection attenuates the correlation in PGSs across distinct sets of chromosomes.

Cross-chromosome correlations in PGSs. The number of autosomes in the haploid set is n (= 22 in humans). Label the set of loci on chromosome k that contribute variation to our trait of interest L_k; the overall set of loci underlying variation in the trait is $L = \{L_1, L_2, \ldots, L_k\}$. We divide the chromosomes into distinct sets K_1 and K_2 (e.g., K_1 could be the set of odd numbered chromosomes and K_2 the even). Let $L^{(1)}$ and $L^{(2)}$ be the sets of causal loci on the chromosomes in K_1 and K_2 respectively (i.e., $L^{(i)} = \cup_{k \in K_i} L_k$).

Suppose that we have accurately estimated effect sizes at all loci $l \in L$. For each individual, we then calculate a polygenic score for K_1 and for K_2:

$$P_1 = \sum_{l \in L^{(1)}} g_l \alpha_l; \quad P_2 = \sum_{l' \in L^{(2)}} g_{l'} \alpha_{l'}.$$

We are interested in the correlation in the population between P_1 and P_2, and in particular, how this correlation is affected by assortative mating and stabilizing selection for the focal trait. The correlation can be written

$$\mathrm{Corr}(P_1, P_2) = \frac{\mathrm{Cov}(P_1, P_2)}{\mathrm{Var}(P_1)\mathrm{Var}(P_2)},$$

with

$$\begin{aligned}
\mathrm{Cov}(P_1, P_2) &= \mathrm{Cov}\left(\sum_{l \in L^{(1)}} g_l \alpha_l, \sum_{l' \in L^{(2)}} g_{l'} \alpha_{l'}\right) \\
&= \sum_{l \in L^{(1)}} \sum_{l' \in L^{(2)}} \mathrm{Cov}\left(g_l, g_{l'}\right) \alpha_l \alpha_{l'} \\
&= 2 \sum_{l \in L^{(1)}} \sum_{l' \in L^{(2)}} \left(D_{ll'} + \tilde{D}_{ll'}\right) \alpha_l \alpha_{l'}. \qquad \text{(S.109)}
\end{aligned}$$

Since, to make progress in the case of stabilizing selection, we will assume effect sizes to be equal across loci, we make that assumption now, so that

$$\mathrm{Cov}(P_1, P_2) = 2\alpha^2 \sum_{l \in L^{(1)}} \sum_{l' \in L^{(2)}} \left(D_{ll'} + \tilde{D}_{ll'}\right). \qquad \text{(S.110)}$$

Since every pair of loci (l, l') across $L^{(1)}$ and $L^{(2)}$ are by definition unlinked, under many processes (including assortative mating and stabilizing selection), the values of $D_{ll'}$ and $\tilde{D}_{ll'}$ will not differ much in expectation across locus pairs, in equilibrium. Therefore, we may approximate $D_{ll'} = D^*$ and $\tilde{D}_{ll'} = \tilde{D}^*$ for all $l \in L^{(1)}$ and $l' \in L^{(2}$, so that Eq. (S.110) simplifies further:

$$\mathrm{Cov}(P_1, P_2) = 2\left|L^{(1)}\right|\left|L^{(2)}\right|\left(D^* + \tilde{D}^*\right)\alpha^2. \tag{S.111}$$

Assortative mating alone. Under assortative mating with equal effect sizes across loci, in equilibrium, LDs are approximately equal across locus pairs, regardless of the recombination rate between them; moreover, cis- and trans-LDs are equal (see above). Therefore, to calculate D^* $(= \tilde{D}^*)$, we simply apportion the total LD given by Eq. (S.55) among individual locus pairs:

$$\frac{h^2\rho}{1-h^2\rho}V_g \approx 4\sum_{l\in L}\sum_{\substack{l'\in L\\ l'\neq l}} D^*_{ll'}\alpha_l\alpha_{l'} = 4|L|(|L|-1)\alpha^2 D^*$$

$$\Rightarrow \quad D^* \approx \frac{\frac{h^2\rho}{1-h^2\rho}V_g}{4|L|(|L|-1)\alpha^2} = \frac{\frac{h^2\rho}{1-h^2\rho}|L|\bar{H}\alpha^2}{4|L|(|L|-1)\alpha^2} = \frac{\frac{h^2\rho}{1-h^2\rho}\bar{H}}{4(|L|-1)} \approx \frac{1}{4}\cdot\frac{h^2\rho}{1-h^2\rho}\cdot\frac{\bar{H}}{|L|}, \tag{S.112}$$

when $|L|$ is large. Similarly,

$$\tilde{D}^* \approx \frac{1}{4}\cdot\frac{h^2\rho}{1-h^2\rho}\cdot\frac{\bar{H}}{|L|}, \tag{S.113}$$

so that the overall contribution of assortative mating to the covariance in Eq. (S.111) is proportional to

$$D^* + \tilde{D}^* \approx \frac{1}{2}\cdot\frac{h^2\rho}{1-h^2\rho}\cdot\frac{\bar{H}}{|L|}. \tag{S.114}$$

Stabilizing selection alone, pre-selection in the focal generation. Under stabilizing selection, the total amount of negative cis-LD present before selection has acted in a given generation is given by Eq. (S.106):

$$2\alpha^2\sum_{l\in L}\sum_{\substack{l'\in l\\ l'\neq l}} D_{ll'} = d = -\frac{V_g}{2\bar{c}_h\left(\frac{1+V_S/V_P}{h^2}\right)+1}, \tag{S.115}$$

where we have dropped the equilibrium '$*$' markers. This expression does not easily decompose into terms from individual locus pairs. However, if we assume that stabilizing selection is relatively weak ($V_S/V_P^* \gg 1$) and that the recombination process is such that the harmonic mean recombination rate $\bar{c}_h \sim 1/2$ (as is the case in humans), Eq. (S.115) can be approximated by

$$2\alpha^2\sum_{l\in L}\sum_{\substack{l'\in l\\ l'\neq l}} D_{ll'} = d \approx -\frac{V_g}{2\bar{c}_h\left(\frac{1+V_S/V_P}{h^2}\right)} = -\frac{1}{2}\cdot\frac{h^2V_g}{1+V_S/V_P}\cdot\frac{1}{\bar{c}_h} = -\frac{1}{2}\cdot\frac{h^2V_g}{1+V_S/V_P}\cdot\frac{2\sum_{l,l'}1/c_{ll'}}{|L|(|L|-1)},$$

from which we infer that, in expectation,

$$2\alpha^2 D_{ll'} \approx -\frac{h^2V_g}{1+V_S/V_P}\cdot\frac{1/c_{ll'}}{|L|(|L|-1)}.$$

Therefore, for unlinked l and l' ($c_{ll'} = 1/2$), in expectation,

$$
\begin{aligned}
D_{ll'} &\approx -\frac{1}{\alpha^2|L|(|L|-1)} \cdot \frac{h^2 V_g}{1+V_S/V_P} = -\frac{\bar{H}}{\alpha^2 \bar{H}|L|(|L|-1)} \cdot \frac{h^2 V_g}{1+V_S/V_P} = -\frac{\bar{H}}{(|L|-1)V_g} \cdot \frac{h^2 V_g}{1+V_S/V_P} \\
&= -\frac{\bar{H}}{|L|-1} \cdot \frac{h^2}{1+V_S/V_P} \approx -\frac{\bar{H}}{|L|} \cdot \frac{h^2}{1+V_S/V_P}.
\end{aligned}
\tag{S.116}
$$

under random mating, before selection has acted in the focal generation, $\tilde{D}_{ll'} = 0$ in expectation. Therefore, under stabilizing selection alone, and before selection has acted in the focal generation, the contribution of an unlinked locus pair to the covariance in Eq. (S.111) is

$$
D^* + \tilde{D}^* = D^* \approx -\frac{\bar{H}}{|L|} \cdot \frac{h^2}{1+V_S/V_P}. \tag{S.117}
$$

Stabilizing selection alone, post-selection in the focal generation. As noted above, for any pair of loci l and l', the amount of cis-LD and the amount of trans-LD generated by stabilizing selection within the focal generation are equal, and equal to $c_{ll'}$ times the degree of cis-LD transmitted from the previous generation, in expectation. Therefore, across pairs of unlinked loci, the sum of the cis- and trans-LD generated by selection within the focal generation is equal to the amount of cis-LD that was transmitted from the previous generation, so that the total amount of cis- and trans-LD after selection has acted is twice the amount that was transmitted from the previous generation. Therefore, we simply need to double Eq. (S.117) to find the total cis- and trans-LD across unlinked locus pairs after selection has acted in the focal generation:

$$
D^* + \tilde{D}^* \approx -\frac{2\bar{H}}{|L|} \cdot \frac{h^2}{1+V_S/V_P}. \tag{S.118}
$$

How much does stabilizing selection attenuate the signal of assortative mating? Comparing Eqs. (S.114) and (S.117), we find that the proportionate attenuation of assortative mating's effect (in isolation) by the action of stabilizing selection across generations, but before stabilizing selection has acted in the current generation, is

$$
\frac{-\frac{\bar{H}}{|L|} \cdot \frac{h^2}{1+V_S/V_P}}{\frac{1}{2} \cdot \frac{h^2\rho}{1-h^2\rho} \cdot \frac{\bar{H}}{|L|}} = \frac{-2}{1+V_S/V_P} \cdot \frac{1-h^2\rho}{\rho}. \tag{S.119}
$$

For example, in the case of human height ($h^2 \sim 0.8$), the signal of assortative mating (strength $\rho \sim 0.25$) is attenuated by stabilizing selection (strength $V_S/V_P \sim 30$) by a proportionate amount of approximately 20%. That is, one might measure by other means (e.g., the phenotypic correlation among mates, together with an estimate of the heritability of height) that the strength of assortative mating is $\rho = 0.25$, but estimating this strength from cross-chromosome PGS correlations without accounting or correcting for stabilizing selection on height would yield $\hat{\rho} \approx 0.2$, 20% smaller than the true value.

From Eqs. (S.114) and (S.118), the proportionate attenuation of assortative mating's effect (in isolation) by the action of stabilizing selection across generations, after stabilizing selection has acted in the current generation too, is

$$
\frac{-\frac{2\bar{H}}{|L|} \cdot \frac{h^2}{1+V_S/V_P}}{\frac{1}{2} \cdot \frac{h^2\rho}{1-h^2\rho} \cdot \frac{\bar{H}}{|L|}} = \frac{-4}{1+V_S/V_P} \cdot \frac{1-h^2\rho}{\rho}. \tag{S.120}
$$

For human height, the signal of assortative mating is attenuated by stabilizing selection by a proportionate amount of approximately 40%; i.e, though the strength of assortative mating is $\rho = 0.25$, estimating this strength from cross-chromosome PGS correlations without correcting for stabilizing selection would yield $\hat{\rho} \approx 0.15$.

S4 Relationship to LD-score regression

In the Main Text, we focused on the influence of various forms of confounding on the average heterozygosity-weighted squared effect-size estimate at genotyped locus λ, $2p_\lambda(1-p_\lambda)\hat{\alpha}_\lambda^2$. We chose this as a metric for the degree of confounding because (i) it is the metric that determines the significance (in terms of p-values) of the locus in a GWAS, (ii) it is an estimate of the locus's contribution to the genic variance of the trait, and (iii) it is proportional to the usual GWAS χ^2-statistic. In this section, we relate this metric—and our analyses of how confounding affects it—to metrics produced by LD-score regression. We initially focus on the within-trait LD-score regression, which can produce estimates of the degree of confounding in the GWAS and the heritability of the trait. We then turn to cross-trait LD-score regression, which can produce estimates of genic correlations between traits.

Note that the original LD-score regression calculations [13,14] involved normalized values of genotypes and variant effect sizes, whereas we use unnormalized values for comparison with our calculations elsewhere in this paper.

Note too that several other papers considered the effect of various forms of confounding on the output of LD-score regression (e.g., [15,16]). Our analysis here is included for completeness.

S4.1 Same-trait genetic variance

Consider a trait affected by genetic variation at a set of bi-allelic loci L. The frequency of the trait-increasing variant at locus $l \in L$ is p_l, and its expected effect on the trait is to increase its value by α_l. Locus l therefore contributes an amount $v_l = 2p_l(1-p_l)\alpha_l^2$ to the genic variance of the trait, $V_g = \sum_{l\in L} v_l$.

A population-based association study is performed at a bi-allelic locus λ, which may or may not be in L (we'll consider both cases separately below). The frequency of the focal variant at λ is p_λ. Assuming that genotype frequencies are approximately in Hardy-Weinberg equilibrium, the estimate of the focal variant's effect size is

$$\hat{\alpha}_\lambda \approx \frac{\sum_{l\in L}\left(D_{\lambda l} + \tilde{D}_{\lambda l}\right)\alpha_l}{p_\lambda(1-p_\lambda)}. \tag{S.121}$$

The χ^2-statistic at λ is then proportional to

$$\begin{aligned}
2p_\lambda(1-p_\lambda)\hat{\alpha}_\lambda^2 &= \frac{2\left(\sum_{l\in L}(D_{\lambda l} + \tilde{D}_{\lambda l})\alpha_l\right)^2}{p_\lambda(1-p_\lambda)} \\
&= \frac{2\sum_{l\in L}(D_{\lambda l} + \tilde{D}_{\lambda l})^2\alpha_l^2 + 2\sum_{l\in L}\sum_{l'\neq l}(D_{\lambda l} + \tilde{D}_{\lambda l})(D_{\lambda l'} + \tilde{D}_{\lambda l'})\alpha_l\alpha_{l'}}{p_\lambda(1-p_\lambda)} \\
&= \sum_{l\in L}(r_{\lambda l} + \tilde{r}_{\lambda l})^2 v_l + \sum_{\substack{l,l'\in L \\ l\neq l'}}(r_{\lambda l} + \tilde{r}_{\lambda l})\sqrt{v_l}\,\mathrm{sgn}\,(\alpha_l)(r_{\lambda l'} + \tilde{r}_{\lambda l'})\sqrt{v_{l'}}\,\mathrm{sgn}\,(\alpha_{l'}),
\end{aligned} \tag{S.122}$$

where $r_{ll'} = D_{ll'}/\sqrt{p_l(1-p_l)p_{l'}(1-p_{l'})}$ and $\tilde{r}_{ll'} = \tilde{D}_{ll'}/\sqrt{p_l(1-p_l)p_{l'}(1-p_{l'})}$ are the cis- and trans-correlation coefficients of allelic state between loci l and l', and $\mathrm{sgn}\,(x) = +1$ if $x > 0$ and $\mathrm{sgn}\,(x) = -1$ if

$x < 0$ (we specify the signs of the effects of the focal alleles, instead of assuming them to be positive as we have done till now, for consistency with the next section on genetic correlations between traits).

The focal locus λ does not itself affect the trait.

If $\lambda \notin L$, the LD between the focal variants at λ and a locus $l \in L$ does not depend on the strength or direction of the effect of the focal variant at l. Therefore, the expectation of the first term on the right-hand side of Eq. (S.122) can be written

$$\mathbb{E}\left[\sum_{l\in L}(r_{\lambda l}+\tilde{r}_{\lambda l})^2 v_l\right] = |L|\mathbb{E}\left[(r_{\lambda l}+\tilde{r}_{\lambda l})^2\right]\mathbb{E}[v_l] = \left(\frac{1}{|L|}\sum_{l\in L}(r_{\lambda l}+\tilde{r}_{\lambda l})^2\right)\sum_{l\in L} v_l = S_\lambda V_g/|L|, \qquad \text{(S.123)}$$

where S_λ is the LD score of locus λ (it is usually denoted l_λ, but we have used l to index causal loci).

The expectation of the second term on the right-hand side of Eq. (S.122) can be written

$$\mathbb{E}\left[\sum_{\substack{l,l'\in L\\ l\neq l'}}(r_{\lambda l}+\tilde{r}_{\lambda l})\sqrt{v_l}\,\mathrm{sgn}\,(\alpha_l)(r_{\lambda l'}+\tilde{r}_{\lambda l'})\sqrt{v_{l'}}\,\mathrm{sgn}\,(\alpha_{l'})\right]$$
$$= \sum_{\substack{l,l'\in L\\ l\neq l'}}\mathrm{Cov}\Big((r_{\lambda l}+\tilde{r}_{\lambda l})\sqrt{v_l}\,\mathrm{sgn}\,(\alpha_l), (r_{\lambda l'}+\tilde{r}_{\lambda l'})\sqrt{v_{l'}}\,\mathrm{sgn}\,(\alpha_{l'})\Big), \qquad \text{(S.124)}$$

since, when $\lambda \notin L$, $\mathbb{E}\left[(r_{\lambda l}+\tilde{r}_{\lambda l})\sqrt{v_l}\,\mathrm{sgn}\,(\alpha_l)\right] = 0$ for $l \in L$.

Therefore, for tag loci λ ($\lambda \notin L$), the expectation of Eq. (S.122) can be written

$$\mathbb{E}\left[2p_\lambda(1-p_\lambda)\hat{\alpha}_\lambda^2\right] = S_\lambda V_g/|L| + \sum_{\substack{l,l'\in L\\ l\neq l'}}\mathrm{Cov}\Big((r_{\lambda l}+\tilde{r}_{\lambda l})\sqrt{v_l}\,\mathrm{sgn}\,(\alpha_l), (r_{\lambda l'}+\tilde{r}_{\lambda l'})\sqrt{v_{l'}}\,\mathrm{sgn}\,(\alpha_{l'})\Big). \qquad \text{(S.125)}$$

If the second, covariance term in Eq. (S.125) is zero, then the regression of $2p_\lambda(1-p_\lambda)\hat{\alpha}_\lambda^2$ on the LD-scores S_λ (here augmented to include trans-LD) returns a slope proportional to V_g. This is the basis of the estimation of heritability in LD-score regression. However, if the covariance term is systematically positive or negative, inflating or deflating $\mathbb{E}\left[2p_\lambda(1-p_\lambda)\hat{\alpha}_\lambda^2\right]$ relative to its LD-score expectation, then LD-score regression will yield a systematically biased estimate of V_g. When should the covariance term be zero, and when should it be systematically positive or negative?

First, note that 'neutral' population structure, in which linkage disequilibria are generated because of the drift of alleles up or down in frequency in different populations/regions, will not cause the covariance term to be systematically positive or negative. If allele frequencies at λ, l, and l' all change across regions/populations, this generates (cis- and trans-) LD between λ and l and between λ and l', but, because drift at l and l' is independent, these LDs are linearly indepdendent of one another, so the covariance term in Eq. (S.125) is zero. Therefore, 'neutral' population structure will not lead LD-score regression to systematically under- or overestimate the heritability [14].

Under same-trait assortative mating, however, the covariance term in Eq. (S.125) is positive. This is because the trait-increasing alleles at l and l' will tend to be in positive LD under same-trait assortative mating, and so, if the focal allele at λ is in positive (resp. negative) LD with the trait increasing allele at l, it will also tend to be in positive (resp. negative) LD with the trait-increasing allele at l'. Same-trait assortative mating will therefore lead LD-score regression to overestimate the heritability of the trait (see

also ref. [16]). The same will hold under other forces that generate systematic positive LD among variants with the same directional effect on the trait, such as trait-biased migration.

Under stabilizing selection, on the other hand, the covariance term in Eq. (S.125) is negative: since the trait-increasing alleles at l and l' will tend to be in negative LD under stabilizing (and other forms of) selection, if the focal allele at λ is in positive (resp. negative) LD with the trait increasing allele at l, it will tend to be in negative (resp. positive) LD with the trait-increasing allele at l'. Stabilizing selection will therefore lead LD-score regression to underestimate the heritability of the trait.

To get a sense of the magnitude of these inflations/deflations of $\mathbb{E}\left[2p_\lambda(1-p_\lambda)\hat{\alpha}_\lambda^2\right]$, assume that $\big((r_{\lambda l}+\tilde{r}_{\lambda l})\sqrt{v_l}\,\mathrm{sgn}\,(\alpha_l),\,(r_{\lambda l'}+\tilde{r}_{\lambda l'})\sqrt{v_{l'}}\,\mathrm{sgn}\,(\alpha_{l'})\big)$ is bivariate normal with correlation coefficient ρ. Then we can write the covariance term in Eq. (S.125) as

$$\begin{aligned}\sum_{\substack{l,l'\in L\\ l\neq l'}}\mathrm{Cov}\big((r_{\lambda l}+\tilde{r}_{\lambda l})\sqrt{v_l}\,\mathrm{sgn}\,(\alpha_l),(r_{\lambda l'}+\tilde{r}_{\lambda l'})\sqrt{v_{l'}}\,\mathrm{sgn}\,(\alpha_{l'})\big) &= \sum_{\substack{l,l'\in L\\ l\neq l'}}\rho\mathrm{Var}\big((r_{\lambda l}+\tilde{r}_{\lambda l})\sqrt{v_l}\,\mathrm{sgn}\,(\alpha_l)\big)\\ &= \rho\sum_{\substack{l,l'\in L\\ l\neq l'}}\mathbb{E}\big[\big((r_{\lambda l}+\tilde{r}_{\lambda l})\sqrt{v_l}\,\mathrm{sgn}\,(\alpha_l)\big)^2\big]\\ &= \rho\sum_{\substack{l,l'\in L\\ l\neq l'}}\mathbb{E}\big[(r_{\lambda l}+\tilde{r}_{\lambda l})^2 v_l\big]\\ &= \rho\sum_{\substack{l,l'\in L\\ l\neq l'}}\mathbb{E}\big[(r_{\lambda l}+\tilde{r}_{\lambda l})^2\big]\mathbb{E}\big[v_l\big]\\ &= \rho S_\lambda V_g/|L|, \qquad\qquad \text{(S.126)}\end{aligned}$$

from Eq. (S.123). Therefore, Eq. (S.125) simiplifies to

$$\mathbb{E}\left[2p_\lambda(1-p_\lambda)\hat{\alpha}_\lambda^2\right] = (1+\rho)S_\lambda V_g/|L|, \qquad \text{(S.127)}$$

and so the slope of the LD score, and therefore the LD-score regression estimate of V_g, is inflated by a factor $1+\rho$.

The focal locus λ does affect the trait.

If $\lambda\in L$, so that $\alpha_\lambda\neq 0$, then, from Eq. (S.122), the χ^2-statistic at λ is proportional to

$$2p_\lambda(1-p_\lambda)\hat{\alpha}_\lambda^2 = v_\lambda + \sum_{\substack{l\in L\\ l\neq\lambda}}(r_{\lambda l}+\tilde{r}_{\lambda l})^2 v_l + \sum_{\substack{l,l'\in L\\ l\neq l'}}(r_{\lambda l}+\tilde{r}_{\lambda l})\sqrt{v_l}\,\mathrm{sgn}\,(\alpha_l)(r_{\lambda l'}+\tilde{r}_{\lambda l'})\sqrt{v_{l'}}\,\mathrm{sgn}\,(\alpha_{l'}). \qquad \text{(S.128)}$$

Now, the terms $(r_{\lambda l}+\tilde{r}_{\lambda l})^2$ and v_l in Eq. (S.128) are no longer independent, since, under forces like assortative mating, the strength of effect of the variants at $l\in L$ will mediate the strength of their LD with the causal variants at λ.

S4.2 Cross-trait genetic correlation

For two traits 1 and 2, given a set of causal loci L at which the reference allele has effect size α_l on trait 1 and β_l on trait 2 (with at least one of these effect sizes being non-zero for each $l\in L$), we can define

the genic covariance of the traits over the set of loci L as:

$$C_g = \sum_{l \in L} 2p_l(1-p_l)\alpha_l \beta_l = \sum_{l \in L} c_l, \tag{S.129}$$

where $c_l = 2p_l(1-p_l)\alpha_l\beta_l$ is the contribution of locus l to C_g. Further define

$$U_g = \sum_{l \in L} 2p_l(1-p_l)\alpha_l^2 = \sum_{l \in L} u_l \quad \text{and} \quad V_g = \sum_{l \in L} 2p_l(1-p_l)\beta_l^2 = \sum_{l \in L} v_l \tag{S.130}$$

to be the genic variances of traits 1 and 2 over L, with u_l and v_l the contributions of locus l.

Given effect size estimates $\hat{\alpha}_\lambda$ and $\hat{\beta}_\lambda$ at locus λ, we consider the quantity $2p_\lambda(1-p_\lambda)\hat{\alpha}_\lambda\hat{\beta}_\lambda$, which might be taken to be a naive estimate of locus λ's contribution to C_g. Since

$$\hat{\alpha}_\lambda \approx \frac{\sum_{l \in L}\left(D_{\lambda l} + \tilde{D}_{\lambda l}\right)\alpha_l}{p_\lambda(1-p_\lambda)} \quad \text{and} \quad \hat{\beta}_\lambda \approx \frac{\sum_{l \in L}\left(D_{\lambda l} + \tilde{D}_{\lambda l}\right)\beta_l}{p_\lambda(1-p_\lambda)}, \tag{S.131}$$

we have

$$\begin{aligned}
2p_\lambda(1-p_\lambda)\hat{\alpha}_\lambda\hat{\beta}_\lambda &= \sum_{l \in L} \frac{2\alpha_l\beta_l\left(D_{\lambda l} + \tilde{D}_{\lambda l}\right)^2}{p_\lambda(1-p_\lambda)} + \sum_{\substack{l,l' \in L \\ l' \neq l}} \frac{2\alpha_l\beta_{l'}\left(D_{\lambda l} + \tilde{D}_{\lambda l}\right)\left(D_{\lambda l'} + \tilde{D}_{\lambda l'}\right)}{p_\lambda(1-p_\lambda)} \\
&= \sum_{l \in L} \frac{2p_l(1-p_l)\alpha_l\beta_l\left(D_{\lambda l} + \tilde{D}_{\lambda l}\right)^2}{p_\lambda(1-p_\lambda)p_l(1-p_l)} + \sum_{\substack{l,l' \in L \\ l' \neq l}} \frac{\sqrt{2p_l(1-p_l)}\alpha_l\left(D_{\lambda l} + \tilde{D}_{\lambda l}\right)\sqrt{2p_{l'}(1-p_{l'})}\beta_{l'}\left(D_{\lambda l'} + \tilde{D}_{\lambda l'}\right)}{\sqrt{p_\lambda(1-p_\lambda)p_l(1-p_l)}\sqrt{p_\lambda(1-p_\lambda)p_{l'}(1-p_{l'})}} \\
&= \sum_{l \in L}(r_{\lambda l} + \tilde{r}_{\lambda l})^2 c_l + \sum_{\substack{l,l' \in L \\ l' \neq l}} (r_{\lambda l} + \tilde{r}_{\lambda l})\sqrt{u_l}\,\mathrm{sgn}\,(\alpha_l)(r_{\lambda l'} + \tilde{r}_{\lambda l'})\sqrt{v_{l'}}\,\mathrm{sgn}\,(\beta_{l'}),
\end{aligned} \tag{S.132}$$

where $\mathrm{sgn}\,(x) = +1$ if $x > 0$ and $\mathrm{sgn}\,(x) = -1$ if $x < 0$. The signs of the effects α_l and β_l are not specified because we cannot assume them to have concordant directions of effect on the two traits.

The focal locus λ does not causally affect the traits.

If $\lambda \notin L$, the expectation of the first term on the right-hand side of Eq. (S.132) can be written

$$\mathbb{E}\left[\sum_{l \in L}(r_{\lambda l} + \tilde{r}_{\lambda l})^2 c_l\right] = |L|\mathbb{E}\left[(r_{\lambda l} + \tilde{r}_{\lambda l})^2\right]\mathbb{E}[c_l] = \left(\frac{1}{|L|}\sum_{l \in L}(r_{\lambda l} + \tilde{r}_{\lambda l})^2\right)\sum_{l \in L} c_l = S_\lambda C_g/|L|, \tag{S.133}$$

where S_λ is the LD score of locus λ. Just as in Eq. (S.124), the expectation of the second term in Eq. (S.132) can be written

$$\begin{aligned}
&\mathbb{E}\left[\sum_{\substack{l,l' \in L \\ l \neq l'}} (r_{\lambda l} + \tilde{r}_{\lambda l})\sqrt{u_l}\,\mathrm{sgn}\,(\alpha_l)(r_{\lambda l'} + \tilde{r}_{\lambda l'})\sqrt{v_{l'}}\,\mathrm{sgn}\,(\beta_{l'})\right] \\
&\qquad = \sum_{\substack{l,l' \in L \\ l \neq l'}} \mathrm{Cov}\Big((r_{\lambda l} + \tilde{r}_{\lambda l})\sqrt{u_l}\,\mathrm{sgn}\,(\alpha_l), (r_{\lambda l'} + \tilde{r}_{\lambda l'})\sqrt{v_{l'}}\,\mathrm{sgn}\,(\beta_{l'})\Big),
\end{aligned} \tag{S.134}$$

Therefore, for tag loci λ ($\lambda \notin L$), the expectation of Eq. (S.132) can be written

$$\mathbb{E}\left[2p_\lambda(1-p_\lambda)\hat{\alpha}_\lambda\hat{\beta}_\lambda\right] = S_\lambda C_g/|L| + \sum_{\substack{l,l'\in L\\ l\neq l'}} \mathrm{Cov}\Big((r_{\lambda l}+\tilde{r}_{\lambda l})\sqrt{u_l}\,\mathrm{sgn}\,(\alpha_l), (r_{\lambda l'}+\tilde{r}_{\lambda l'})\sqrt{v_{l'}}\,\mathrm{sgn}\,(\beta_{l'})\Big). \quad \text{(S.135)}$$

If the second, covariance term on the right-hand size of Eq. (S.135) is zero, then the regression of $2p_\lambda(1-p_\lambda)\hat{\alpha}_\lambda\hat{\beta}_\lambda$ on the LD scores S_λ will yield a slope proportional to C_g—this is the basis of LD-score regression estimation of the genetic correlation of traits. However, if the covariance term in Eq. (S.135) is systematically positive or negative, then LD-score regression will produce a biased estimate of the genic covariance C_g.

Again, neutral population structure will not cause the covariance term in Eq. (S.135) to be systematically negative or positive, and therefore will not lead to biases in LD-score regression estimation of genetic correlations [13].

However, consider positive cross-trait assortative mating between traits 1 and 2. This will tend to generate positive LD between variants that increase trait 1 ($\alpha_l > 0$) and variants that increase trait 2 ($\beta_{l'} > 0$). Therefore, if the focal variant at the tag locus λ is in positive (resp. negative) LD with a trait-1-increasing variant at $l \in L$, it will tend also to be in positive (resp. negative) LD with the trait-2-increasing variant at $l' \in L$. The result is a positive covariance between $(r_{\lambda l}+\tilde{r}_{\lambda l})\sqrt{u_l}\,\mathrm{sgn}\,(\alpha_l)$ and $(r_{\lambda l'}+\tilde{r}_{\lambda l'})\sqrt{v_{l'}}\,\mathrm{sgn}\,(\beta_{l'})$; i.e., a positive covariance term in Eq. (S.135).

Therefore, if the underlying genic correlation between traits 1 and 2 is positive, cross-trait assortative mating will tend to inflate LD-score regression's estimate of this genic correlation. On the other hand, if the underlying genic correlation is negative, positive cross-trait assortative mating will tend to attenuate LD-score regression's estimate of the genic correlation.

Again, to get a sense of the magnitude of these biases, we assume that $\big((r_{\lambda l}+\tilde{r}_{\lambda l})\sqrt{u_l}\,\mathrm{sgn}\,(\alpha_l), (r_{\lambda l'}+\tilde{r}_{\lambda l'})\sqrt{v_{l'}}\,\mathrm{sgn}\,(\beta_{l'})\big)$ is bivariate normal with mean $(0,0)$, variance (σ^2,σ^2), and correlation ρ. Then, performing a similar calculation to that in Eq. (S.126), we find that Eq. (S.135) simplifies to

$$\mathbb{E}\left[2p_\lambda(1-p_\lambda)\hat{\alpha}_\lambda\hat{\beta}_\lambda\right] = S_\lambda C_g/|L|\left(1+\rho\frac{V_g}{C_g}\right), \quad \text{(S.136)}$$

where V_g is the genic variance of traits 1 and 2, here assumed to be equal.

The focal locus λ does causally affect the traits.

If $\lambda \in L$, then $\alpha_\lambda \neq 0$ or $\beta_\lambda \neq 0$ (or both). Eq. (S.132) then becomes

$$2p_\lambda(1-p_\lambda)\hat{\alpha}_\lambda\hat{\beta}_\lambda = c_\lambda + \sum_{\substack{l\in L\\ l\neq\lambda}}(r_{\lambda l}+\tilde{r}_{\lambda l})^2 c_l + \sum_{\substack{l,l'\in L\\ l'\neq l}}(r_{\lambda l}+\tilde{r}_{\lambda l})\sqrt{u_l}\,\mathrm{sgn}\,(\alpha_l)(r_{\lambda l'}+\tilde{r}_{\lambda l'})\sqrt{v_{l'}}\,\mathrm{sgn}\,(\beta_{l'}), \quad \text{(S.137)}$$

Now, $(r_{\lambda l}+\tilde{r}_{\lambda l})^2$ and c_l can no longer be taken to be independent. Suppose, for example, that $\alpha_\lambda > 0$, and that $c_l \neq 0$ for some $l \neq \lambda$. Then, under cross-trait assortative mating for traits 1 and 2, the value of β_l mediates both the size of c_l and the degree of LD between the focal variants at λ and l.

References

1. Okbay A, Wu Y, Wang N, Jayashankar H, Bennett M, Nehzati SM, et al. Polygenic prediction of educational attainment within and between families from genome-wide association analyses in 3 million individuals. Nature Genetics. 2022;54(4):437–449.

2. Crow JF, Felsenstein J. The effect of assortative mating on the genetic composition of a population. Eugenics Quarterly. 1968;15(2):85–97.

3. Felsenstein J. Continuous-genotype models and assortative mating. Theoretical Population Biology. 1981;19(3):341–357.

4. Wright S. Systems of mating. III. Assortative mating based on somatic resemblance. Genetics. 1921;6(2):144–161.

5. Crow JF, Kimura M. An Introduction in Population Genetics Theory. New York: Harper and Row; 1970.

6. Nei M, Li WH. Linkage disequilibrium in subdivided populations. Genetics. 1973;75(1):213–219.

7. Bulmer MG. The effect of selection on genetic variability. American Naturalist. 1971;105(943):201–211.

8. Bulmer MG. Linkage disequilibrium and genetic variability. Genetics Research. 1974;23(3):281–289.

9. Sanjak JS, Sidorenko J, Robinson MR, Thornton KR, Visscher PM. Evidence of directional and stabilizing selection in contemporary humans. Proceedings of the National Academy of Sciences. 2018;115(1):151–156.

10. Lee JJ, Wedow R, Okbay A, Kong E, Maghzian O, Zacher M, et al. Gene discovery and polygenic prediction from a genome-wide association study of educational attainment in 1.1 million individuals. Nature Genetics. 2018;50(8):1112–1121.

11. Yengo L, Robinson MR, Keller MC, Kemper KE, Yang Y, Trzaskowski M, et al. Imprint of assortative mating on the human genome. Nature Human Behaviour. 2018;2(12):948–954.

12. Yamamoto K, Sonehara K, Namba S, Konuma T, Masuko H, Miyawaki S, et al. Genetic footprints of assortative mating in the Japanese population. Nature Human Behaviour. 2023;7(1):65–73.

13. Bulik-Sullivan BK, Finucane HK, Anttila V, Gusev A, Day FR, Loh PR, et al. An atlas of genetic correlations across human diseases and traits. Nature Genetics. 2015;47(11):1236–1241.

14. Bulik-Sullivan BK, Loh PR, Finucane HK, Ripke S, Yang J, Patterson N, et al. LD Score regression distinguishes confounding from polygenicity in genome-wide association studies. Nature Genetics. 2015;47(3):291–295.

15. Border R, Athanasiadis G, Buil A, Schork A, Cai N, Young A, et al. Cross-trait assortative mating is widespread and inflates genetic correlation estimates. Science. 2022;378(6621):754–761.

16. Border R, O'Rourke S, de Candia T, Goddard ME, Visscher PM, Yengo L, et al. Assortative mating biases marker-based heritability estimators. Nature Communications. 2022;13(1):1–10.
