# Supplementary material for: Interpreting population- and family-based genome-wide association studies in the presence of confounding
Source: PLoS Biol. 2024 Apr 11;22(4):e3002511. doi: 10.1371/journal.pbio.3002511 (PMC11008796; doi:10.1371/journal.pbio.3002511)
Supplement: S1 Fig — Simulations are the same as in Fig 2; the code can be found at https://doi.org/10.5281/zenodo.10520811. (PDF) [file pbio.3002511.s002.pdf]

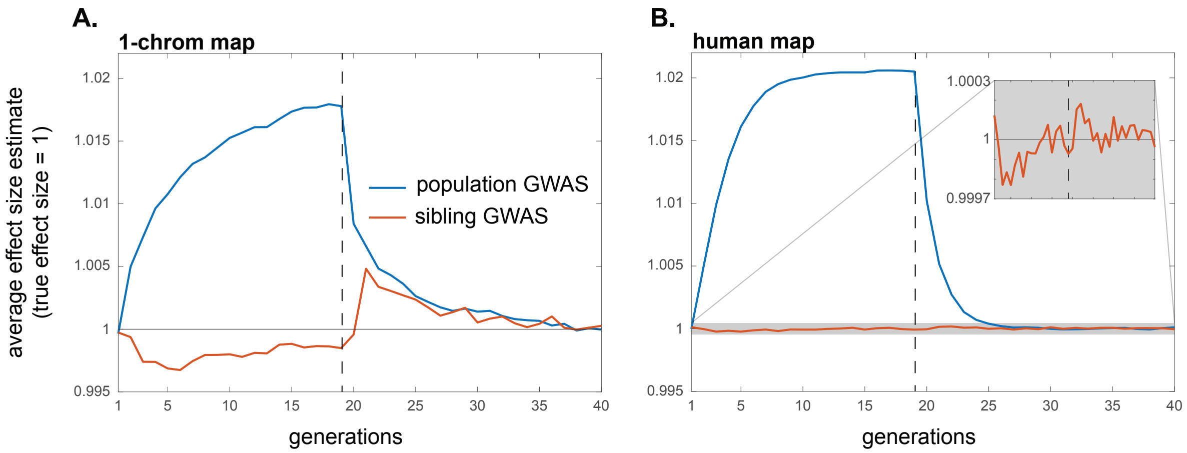

Fig S1: Cross-trait assortative mating influences effect-size estimates at loci that affect the study trait, although this influence is second-order relative to that on effect size estimates at loci that do not affect the study trait but do affect the other trait involved in assortative mating (cf. Fig 2; note the scales of the y-axes). Simulations are the same as in Fig 2; the code can be found at `https://doi.org/10.5281/zenodo.10520811`.
