# Supplementary material for: Interpreting population- and family-based genome-wide association studies in the presence of confounding
Source: PLoS Biol. 2024 Apr 11;22(4):e3002511. doi: 10.1371/journal.pbio.3002511 (PMC11008796; doi:10.1371/journal.pbio.3002511)
Supplement: Attachment — Submitted filename: Veller_Responses.pdf [file pbio.3002511.s004.pdf]

Dear Dr. Roberts

Thank you for your consideration of our manuscript, "Interpreting population- and family-based association studies in the presence of confounding". We are grateful to the reviewers for their thoughtful comments, which have substantially improved the manuscript. Our responses are in blue font below. Line numbers refer to the revised version of the manuscript unless noted otherwise.

We have made two substantive revisions to the manuscript in addition to those in response to the reviewers' comments. The first is to have included, in the section on the effects of stabilizing selection on GWAS estimates, the case where selection has already acted in the generation from which the GWAS sample is taken. This approximately doubles the biases in population-GWAS effect-size estimates induced by stabilizing selection, relative to the case that we treated in the previous submission, where selection has not yet acted in the generation from which the GWAS sample is taken (see Section 3.4, Fig. 6B, and Appendices A3.4.2–4 in the revised manuscript).

The second is to have removed the section on GxE interactions. As Dr. Young notes in his review, these do not really constitute "confounds". In work elsewhere, we have studied the issues that GxE interactions can raise for causal interpretations of within-family polygenic score associations, and we feel that the single-locus GxE results from our previous submission are better suited to that manuscript. Removing the GxE section has also allowed us to cut down the length of the manuscript, in response to Dr. Young's concern that it was too long.

Sincerely,
Carl Veller and Graham Coop

Reviewer #1:
[identifies himself as Alexander Young]
IMPORTANT: See also the comments on the attached PDF!

The authors present a manuscript that evaluates the influence of a variety of factors on both standard and family-based GWAS methods. The manuscript represents an important contribution to the literature on understanding what different GWAS designs are estimating, a topic of considerable importance since so much in human genetics relies upon the output of these studies. The results that I found most enlightening were those detailing how different population genetic processes can lead to forms of within-chromosome linkage disequilibrium (LD) that influence family-based and standard GWAS designs differently. Also important is the authors' warning about the interpretation of within-family analyses of polygenic predictors (PGS) derived from standard GWAS owing to the influence of confounding in the original GWAS summary statistics.

While I found the manuscript made many valuable points, there are some issues with the theoretical results on family-based GWAS and on the definitions of 'bias' and 'confounding' the authors use. I outline the major issues here, with detailed comments given in an annotated PDF.

We are extremely grateful to Dr. Young for his detailed comments, both here and in the pdf.

The first issue is the authors' derivation of results for family-based GWAS using transmitted and non-transmitted alleles. The authors define this method as proceeding by performing two separate univariate regressions on transmitted and untransmitted parental alleles, with direct effects estimated from the difference of these regression coefficients (A1.4). This is not the methodology used in family-based GWAS studies (e.g. Young et al. 2022) or in studies of indirect genetic effects (e.g. Kong et al. 2018). The method used in Young et al. 2022 is a regression of offspring phenotype jointly onto offspring genotype (or PGS) and combined parental genotype (or PGS). This is equivalent to a joint regression onto genotypes computed from the transmitted and non-transmitted parental alleles, with the direct effect computed by subtracting the coefficient on the non-transmitted genotype from the coefficient on the transmitted genotype, the method used by Kong et al. 2018. The equivalence between these two models is shown in Section 7.2 of the Supplementary Information of Okbay et al. 2022. While this is equivalent to performing separate regressions on transmitted and non-transmitted genotypes under random-mating, which means transmitted and non-transmitted genotypes (or PGS) are uncorrelated, this is not true in general, including for many of scenarios considered in the manuscript. I'm not sure if this makes much difference in some scenarios (e.g. analysis of a single variant affecting a polygenic phenotype affected by assortative mating) but could make a substantial difference under others (e.g. strong population structure, or a PGS that is strongly correlated between parents). However, most of the results in the main text use the sibling design, for which the author's derivation is correct as far as I can tell.

We are grateful to Dr. Young for pointing this out. We have now reworked our calculations for the case where the phenotype is regressed jointly on genotypes constructed from the transmitted and untransmitted alleles (Appendix pp. 44-49; Main Text Eqs. 5, 6, 8, 9). The conclusions are essentially unchanged: The trio-based estimate of the direct effect differs from our previous calculation by a factor (1-F)/(1+F) and is now equal to the sibling-based estimate in expectation (Eq. 6). The estimate of the indirect effect still absorbs genetic (direct and indirect) and environmental confounding (Eqs. 8, 9) and, as Dr. Young points out, equals our previous calculation in the case where F=0 (Eq. 9).

The second issue is how the authors define bias or confounding. This is a tricky issue in the case of GWAS designs since they are estimating marginal effects, which are inherently defined in terms of linkage disequilibrium with other variants. The authors define the true effect to be that of the local region around the focal SNP, i.e. the region with recombination fraction approximately 0. But this is not what GWAS is estimating even under random-mating with direct genetic effects only: it is estimating a linear combination of direct genetic effects of causal variants on the same chromosome, with weight in proportion to the LD between the focal variant and the causal variant. It would be better to define the true target parameter as such (as would be obtained from standard GWAS under random-mating with no indirect genetic effects) and then to examine bias in relation to that parameter.

We agree with Dr. Young that the effect-size estimate at a given genotyped locus is intended to capture effects of causal loci with which the genotyped locus is in LD. However, we disagree that researchers have in mind the entire chromosome of the genotyped locus, or that this is a sensible definition of what the association study at the genotyped locus is meant to capture, for several reasons. First, under random mating, the expected strength of LD between the genotyped locus and a causal locus rapidly drops off as their recombination fraction grows (e.g., McVean 2002)—to define "local" in terms of LD under random mating is therefore effectively to restrict the region to a small recombination distance around the genotyped locus. Therefore, in practice, our "local" region is designed to span the extent of physical LD under random mating, not just a region with r=0, and so captures the GWAS result obtained under random mating. Second, because the recombination fraction between a genotyped locus and distant loci on the same chromosome can approach 1/2—i.e., the recombination fraction between

the genotyped locus and loci on other chromosomes—we see no principled reason to restrict attention to loci on the same chromosome as the genotyped locus and not the whole genome.

We have now added more clarification to our definition of "local" in lines 138-148, making it somewhat more concrete. More generally, we agree that all definitions here, including our definition of "local", are necessarily arbitrary, and we have included a discussion of this point in lines 138-148.

A related issue is the authors examine the impact of various population genetic processes on a quantity they term the "heterozygosity weighted average squared effect size estimate". An issue with this quantity is that GWAS gives a biased estimate of the true value of this even under random-mating and direct genetic effects only (e.g. Figure 3). It therefore makes it hard to assess the impact of various population genetic processes on the bias introduced into this term, and it makes me doubt whether it is even a sensible thing to estimate with marginal GWAS effect estimates, whether from standard or family-based GWAS. It would both be clearer and increase the impact of this paper on the field if the authors could measure bias in some quantity or method that is generally used and (at least thought to be) understood, such as SNP heritability as estimated by LDSC. LDSC attempts to 'undo' the fact that marginal GWAS effect estimates are the outcome of LD with variants that are physically close to the focal variant when estimating SNP heritability. Results demonstrating how various population genetic processes impact LDSC estimates of SNP heritability (which should be approximately unbiased under a model of random mating and direct genetic effects only) from standard and family-based GWAS would be far more interesting and interpretable than results on 'heterozygosity weighted average squared effect size estimate'. (I appreciate there is a complicating factor that the true heritability changes with these different population genetic processes, but that is something I'm sure the authors could find a sensible way of dealing with and communicating. True SNP heritability could, for example, be computed from a PGS computed from the true direct effects of the causal SNPs.)

We would strongly prefer to keep our Main Text analysis in terms of the heterozygosity-weighted squared effect-size estimate. This metric plays a central role in GWAS: it is proportional to the chi-squared statistic, and therefore governs the statistical significance of the association detected at the locus. In addition, it plays a key role in downstream analyses such as LD-score regression. We agree that other metrics are of practical use, and have now added a section to our Appendix discussing how our analysis of confounding carries over to LD-score regression (Appendix pp. 75-80). We note that the mathematics of the LD score regression with confounding adds even more confusing terms, and so we are not sure that the average reader would benefit from grappling with it directly.

I also think the authors are stretching the definition of 'confounding' when they claim GxE and GxG interactions lead to confounding in family-based GWAS estimates. Under interaction models, all marginal effects are some sort of average effect, with the average taken over some distribution of the interacting factors. Such marginal effects are not typically thought of as 'confounded' by interactions in statistical modeling. The authors point that family-based and standard GWAS are estimating different average effects under certain interaction models is valid, but I don't think this can be construed as 'confounding' in family-based GWAS.

We agree with Dr. Young, and it was not our intention to include GxE and GxG interactions as confounding per se. However, we agree that the presentation of the interactions results alongside the main confounding results could lead to that confusion. For this reason, we have removed the section from the manuscript, and an expanded version now forms the basis of a related paper.

The authors should also include a description of the simulations in the manuscript. I followed the link to the git repository, and did not find it immediately enlightening.

The simulations used to produce each Results figure are described in substantial detail in the Methods section (pp. 29-31), and we have now added a README file to the github directory describing which script in the directory generates the data for which figure in the text.

Finally, the main text is rather long. While I thought the authors generally did a good job of making a technical topic more comprehensible, the writing could be tightened up significantly.

We have attempted to cut words where possible. Removing the GxE section has also helped with shortening the manuscript.

Alexander Young.

Reviewer #2:

This is a fantastic study by Veller & Coop that lays out how population structure impacts estimates of SNP effects and other derived quantities obtained across various experimental designs (population and within-family designs).

We are grateful to the reviewer for their positive assessment of our work.

I only have minor comments.

My first comment relates to the word "bias", which seems overly used in this manuscript (x47 times). I will illustrate my point by simply talking about heritability. The heritability of a trait has a different meaning (and expectation) depending on whether the population undergoes random mating or not. Therefore, assuming that the random mating heritability is the true heritability and the other one biased does not seem to be the appropriate language. Even more so, given that a randomly mating population is a mere abstraction that would probably never exist in nature. The paper has a lot of this fallacious dichotomy. This is a minor comment so the only action that I request here is for the authors to emphasize that the notion of bias is subjective and will depend on what the application is.

We have now included a note early in the Results section (lines 159-164) discussing the meaning of “bias” and “confounding” in GWASs.

My second comment regards the effectiveness of PCA to correct stratification. The authors echo what was reported before that PCA adjustment may not fully remove confounding. However, this is only half true as the power of PCA depends on sample size. Interestingly, Price (2006) clearly made that point and gave a rule of thumb to appreciate when PCA starts detecting (sub)structures. However, that argument tends to get lost in the literature. My suggestion is to emphasize that the power of PC depends on sample size or, equivalently, to add that PCA is inefficient in (meta-analyses of) small samples.

This is a good point, and we have now noted this sensitivity of PCA to sample size, citing Patterson, Price, and Reich (2006).

"Transmitted genotype" - Only alleles are transmitted. Please fix.

We agree with the reviewer that this terminology is not technically correct. However, we feel that it is a useful shorthand for the more cumbersome "genotypes constructed from transmitted alleles", and is unlikely to be misunderstood. We have now flagged our use of this terminology in lines 200-202.

Reviewer #3:
[identifies himself as Gibran Hemani]

Veller and Coop provide a very detailed and careful theoretical analysis of subtle biasing structures in different GWAS designs, which I personally found to be valuable and educational. The authors use their analysis to propose caution over family-based methods for genetic association analysis, however a lack of contextualising with empirically known parameters of biasing mechanisms made this feel difficult to easily accept or action.

We are grateful to Dr. Hemani for his positive assessment of our paper.

Main comments

Mechanistic processes. I did struggle with explanations such as "Genetic confounding is caused by long-range LD between loci that affect the trait or traits under study" which are used throughout - it equates an unobservable state of the data with an observable state of the data, however it would be better to equate a mechanistic process (e.g. stabilising selection, cross-trait assortative mating etc) with an observable state of the data (long range LD). Perhaps avoid the term genetic confounding. It appears that there are cross-trait mechanisms that introduce long-range LD, and within-trait mechanisms that drive issues at the causal locus. So start with the mechanisms, list what types of LD they can generate, and summarise the extent of bias likely arising from those mechanisms.

Our usage of "genetic confounding" in this paper, referring to the effect of distant causal loci with which a study locus is in LD on effect-size estimation at the study locus, is standard in the GWAS literature (e.g., Nordborg & Vilhjalmsson 2013). Moreover, it is consistent with standard usage of the word "confound" in regression theory, since these distant loci cause omitted variable bias in the marginal regression at the focal locus (we have now noted this in lines 161-163).

Empirical support. Many papers have focused on showing bias in population-based estimates. However few examine bias in family-based estimates, though increasing numbers of family-based estimates are appearing. Can you pinpoint specific examples of family-based estimates which are particularly liable to be incorrect due to some of these biasing mechanisms?

The aim of our paper is to show theoretically that, contrary to common assumption, genetic confounding does affect family-based GWASs. As we have indicated (e.g., Abstract and lines 250-255 in the previous version of the manuscript), the biases caused by genetic confounding in family GWASs are likely often to be small (in humans). Whether this will always be so is a matter for future empirical work.

Sensitivity analysis. A useful outcome from this would be to allow researchers to examine the sensitivity of an estimate by being able to plug in parameters into some of the equations to explore

how much e.g. assortative mating would be required to give rise to the observed results from their study.

We agree with Dr. Hemani, and have now added a worked numerical example of the amplification of effect-size estimates under same-trait assortative mating for height (lines 418-420).

Polygenicity. The simulations used quite low polygenicity (100 or 1000 causal variants). The more socially driven traits that are liable to many of the biasing mechanisms are likely much more polygenic. How sensitive are some of the conclusions to greater polygenicity.

We are limited by computational power in the number of loci that we can include in our SLiM simulations. Apart from the analysis in Fig. 4, which was intended to highlight a particular difference between analyses of traits underlain by fewer vs. more loci, we are confident that our simulation results would be robust to the inclusion of more loci, since they are concordant with our analytical results which in fact hold most precisely in the limit of infinitely many loci.

Tagging SNPs vs other forms of LD. I think it's quite well understood that sentinel variants detected by genome wide scans are not necessarily the causal variants and that they may slightly underestimate the true causal effect of the locus. The other forms of LD bias are much less well known in my opinion. In the exposition it would be anchoring to have a clear distinction between these two ideas at the outset.

We agree, and have now made this distinction more concrete in lines 138-148.

Minor comments

line 31 - it's more accurate to describe assortative mating as a collider/selection bias rather than confounding.

The primary genetic consequence of assortative mating is long-range linkage disequilibria, and therefore genetic confounding. We do not see it as collider bias, which arises when the treatment (here, genotype at focal locus) and outcome (here, phenotype) both influence a third "collider" variable, and the collider is controlled for in the analysis.

line 52 - could say that indirect/dynastic effects are particularly difficult to control using population-level covariates

Agreed—done.

line 52 - 'there is no bright line separating dynastic, environmental and genetic confounding' - The mechanism of dynastic effects is quite specific, and forms a special case of both environmental confounding. I think it would serve the reader better to clarify these distinctions rather than imply that they can't be clarified.

We have removed this sentence.

line 118 - perhaps italicise "other parent"

Done.

Trans-LD - can you provide a more clear mechanistic explanation about how this manifests? It seems like cross-trait assortative mating is the main driver? In any case I would suggest renaming some of these terms.

Trans-LD is generated by several of the processes that we study, e.g., same- and cross-trait assortative mating (lines 394-398, 400-412, Figs. 2 & 3, Appendix A3.1), neutral population structure (line 510, Fig. 5, Appendix A3.2), and stabilizing selection (lines 675-677, Appendix A3.4.2). The terminology "cis-LD" and "trans-LD" has been used before (e.g., Greenspoon & Otto 2009) and, we believe, is more direct and less cumbersome than alternatives like "gametic phase disequilibrium" for cis-LD and "nongametic disequilibrium" for trans-LD.

Line 249 - is it just sampling error that would drive differences in coupling and repulsion heterozygote frequencies or some other mechanism? If sampling error, does it contribute towards asymptotic bias? If there's a mechanism that drives differences then it would be more direct to re-factor this equation in terms of parameters for that mechanistic process.

Differences in the frequencies of coupling and repulsion double-heterozygotes reflect differences in the degrees of cis- and trans-LD (line 269), and differences in the degrees of cis- and trans-LD can be generated by several of the processes that we study, e.g., same- and cross-trait assortative mating (lines 400-412, Figs. 2 & 3), admixture (lines 596-603, Fig. 5, Appendix A3.3), and stabilizing selection (lines 675-677, Appendices A3.4.1 & A3.4.2).

Line 333 - Determining if a slope is non-zero depends on hypothesis testing which has a power component. There hasn't been any discussion of the magnitude of the biasing terms or their standard errors, i.e. for what sample size of sibling pairs, and for what level of cross trait assortative mating, polygenicity etc would be required in order for a sibling pair PGS slope to be determined to be non-zero?

We agree, but our aim in this section is to show that, even if a well-powered study does find a non-zero within-family slope on the PGS, this does not necessarily indicate an underlying causal relationship.

Line 394 - the h^2 rho term is a bit unclear when there are two traits in cross-trait AM, each potentially having different h^2

We have clarified this sentence, which involves an assumption for simplicity that the two traits have equal heritability, and referred to the Appendix where the general analysis does not make this assumption.

Figure 3 - I think squared effects are being used to proxy h2-like metrics, however there is an issue with bias in h2 due to changes in phenotypic variance due to AM. Kemper et al 2021 discuss how there isn't an obvious 'true' value for h2

We agree, and have now noted this point (and cited the Kemper et al paper) in lines 449-451.
