# Supplementary material for: Interpreting population- and family-based genome-wide association studies in the presence of confounding
Source: PLoS Biol. 2024 Apr 11;22(4):e3002511. doi: 10.1371/journal.pbio.3002511 (PMC11008796; doi:10.1371/journal.pbio.3002511)
Supplement: Attachment — Submitted filename: Veller_Responses.pdf [file pbio.3002511.s005.pdf]

REVIEWER'S COMMENTS:

Reviewer #1:
[identifies himself as Alexander Young]

The authors have done a good job at justifying their choice of the heterozygosity weighted squared effect size as their metric of potential bias. While I personally would still be interested to see simulation results applying LDSC to the different scenarios examined in this manuscript, that is perhaps beyond the scope of what is already a comprehensive manuscript and which now includes a theoretical treatment of potential LDSC biases within the authors' theoretical framework.

We are grateful to Dr. Young for his extremely conscientious and helpful reviews of the two versions of our manuscript.

The authors have corrected their model for trio-based GWAS estimation. However, I was looking at their equations for the non-transmitted coefficient, and I'm not sure that it is totally correct. Looking at Equation 9, the 'genetic confounding direct' component appears to imply that this coefficient can pick up some of the direct effect of the focal variant. If I set \lambda=l, so that c_{\lambda,l}=0, this equation implies a contribution from a term equal to (\tilde{D}'_{l,l}+\tilde{D}_{l,l})\alpha_l^d, i.e. the sum of trans-LD in the offspring and parent generations at locus l times the direct genetic effect of the locus. I do not see how the non-transmitted coefficient can pick up part of the direct effect of the focal variant in a joint regression of phenotype onto genotype and parental genotype, since the direct effect of the focal locus will be fully captured by the offspring genotype at that locus, even when there is trans-LD within the locus. Can the authors comment on this?

(There is a simpler route to deriving the trio-GWAS regression coefficients that avoids having to deal with the complexities of the full joint regression calculations. One can reformulate the regression in terms of the offspring deviation genotype — g_l-(g_l^m+g_l^f)/2 — and the sum of the parents' genotypes. Since these terms have zero covariance even under non-random mating, one can obtain the coefficients for the univariate regressions of phenotype onto these two terms and then take the sum and rearrange in order to obtain the results for the joint regression of phenotype onto offspring and parental genotype. I take this approach in some lectures I gave available here: https://www.youtube.com/watch?v=Imc_gJyhahQ&ab_channel=SocialScienceGeneticsAssociationConsortium)

We have carried out the alternative version of the calculation suggested by Dr. Young, and obtain the same result as our Eq. (A.27), a simplified form of which is Eq. (9) in the Main Text. Both versions of the calculation are quite involved, and, since they return the same answer, we prefer to keep our original version.

Dr. Young is correct that direct genetic effects at the study locus and at loci in its immediate vicinity should not affect the coefficient on the untransmitted genotype at the study locus. This is consistent with our calculation, since, by our definition of loci that are "local" to the study locus, for such loci l, the assumption of $F = 0$ at the study locus λ implies that the trans-LD terms $\widetilde{D}_{\lambda l}$ and $\widetilde{D}'_{\lambda l}$ are zero. Using this implication explicitly in Eq. (9) has now allowed us to simplify it even further, and we are grateful to Dr. Young for having prompted this further simplification.

Also, on lines 1456-1458, the authors invoke the Frisch-Waugh-Lovell theorem to justify performing a regression on the offspring deviation genotype in order to derive the 'direct genetic effect' estimate. But they state that they only need to residualise the offspring genotype for the midparent genotype, whereas the FWL theorem also requires that the phenotype be residualised with respect to the midparent genotype.

The two versions of the FWL theorem are equivalent because the fitted values that are removed from the residualized offspring phenotypes are uncorrelated with the residualized offspring genotypes (see, e.g., Angrist & Pischke, *Mostly Harmless Econometrics*, pg. 35–36).

A couple of minor things:

line 1553: should this be a '+' not an '='?

Correct—fixed (and have added a clarification that $\alpha_l^i = (\alpha_l^{i,f} + \alpha_l^{i,m})/2$ in that equation).

Line 2350: 'teh' -> 'the'

Fixed.
